# Structural basis for independent pore function of Vpb4 from Bacillus thuringiensis

**DOI:** 10.1038/s41467-026-74567-y

**Published:** 2026-07-01

**Authors:** Raymond Wirawan, W. David Jamieson, Hannah M. Baird, Colin Berry, Christopher J. Lupton, Hari Venugopal, Luis E. Valentin-Alvarado, Hannah L. Best, D. Dafydd Jones, Lainey J. Williamson, Husam Sabah Auhim, Oliver K. Castell, Michelle A. Dunstone, Bradley A. Spicer

**Affiliations:** 1 Biomedicine Discovery Institute, Monash University, Clayton, Victoria Australia; 2 School of Pharmacy and Pharmaceutical Sciences, Cardiff University, Cardiff, UK; 3 School of Biosciences, Cardiff University, Cardiff, UK; 4 Ramaciotti Centre for Cryo-Electron Microscopy, Monash University, Clayton, Victoria Australia; 5 Department of Biology, College of Science, University of Baghdad, Baghdad, Iraq

**Keywords:** Cryoelectron microscopy, Phylogeny, Permeation and transport, Bacterial toxins, Electron microscopy

## Abstract

The Bacterial_Exotoxin_B family constitutes translocating pore-forming proteins that function as the binding (B) component in the binary AB Toxin mechanism. While the two-component system of the family is consistent among well-characterised members, the single-component Vpb4 subclass from entomopathogenic *Bacillus thuringiensis* challenges this dogma. Here, through single-particle cryo-electron microscopy, we elucidate the inserted pore structure of a Vpb4 member, Vpb4Aa2, at 2.2 Å resolution. The structure reveals distinguishing features from other family members: missing molecular bottleneck and neutrally charged β-barrel. Accordingly, preliminary electrophysiology studies show greater ion flux by Vpb4Aa2 compared to archetypal family member, PA. Through our findings, structure-guided database search allows identification of putative Vpb4-like proteins, which suggest a broader class of single-component proteins within the larger family. This provides mechanistic understanding of the differences between independent pores and translocating pores, with implications in the agricultural industry for screening and identification of future pest control candidates.

## Abstract (web summary)

Bacterial Exotoxin B proteins are typically binary pore-forming toxins. Here, authors characterise Vpb4, a homologue from entomopathogenic Bacillus thuringiensis, and reveal the structural basis enabling its independent activity.

## Introduction

The bacterium *Bacillus thuringiensis* (Bt) is well known to produce invertebrate-active proteinaceous toxins during sporulation, when these proteins are deposited as natural crystals associated with the spores. In addition, many strains of Bt also produce distinct toxins during their vegetative state. These vegetative proteins are structurally categorised into multiple classes, including the Vip (formerly Vip3), Vpa (formerly Vip2), and Vpb (formerly Vip1 and Vip4) classes^1^.

The Vpb proteins are members of the Bacterial_Exotoxin_B family (Interpro: IPR003896), being structurally related to the well-known Protective Antigen (PA) of *Bacillus anthracis*, C2-II toxin of *Clostridium botulinum*, CdtB of *Clostridioides difficile*, Iota-B of *Clostridium perfringens* and other bacteria from the taxonomic phylum *Bacillota*^2,3^. Functional characterisations reveal a recurring pattern of a two-component AB toxin delivery system, where the pore forming family members function as the binding (B) component for receptor recognition and toxin delivery^3,4^. Upon binding, the family members oligomerise to form pores that act as a translocase, facilitating the entry of the secondary active (A) component with diverse enzymatic functions. Examples of such enzymes include ADP ribosyltransferases (Iota-A, CdtA)^5,6^, zinc metalloproteinases (LF)^7^, or adenylate cyclases (EF)^8^.

The Bacterial_Exotoxin_B family members are generally expressed in their pro-form, where removal of the pro-domain is critical for oligomerisation and function^3^. Upon oligomerisation, the complex is then able to perforate the membrane. Several triggers for pore formation have been reported, such as acidification within the endosomal environment (PA)^9^, receptor binding (CdtB)^10,11^ or calcium depletion (CdtB)^12^. Once the pore is formed, the archetypal family members function by translocating their enzymatic partner component into the target cytoplasm using one of two hypothesised mechanisms: (1) the extended-chain Brownian ratchet^9,13^, or (2) the allosteric helix-compression mechanism^14^. This translocation event is thought to occur upon endocytosis of the complex and is driven by the proton gradient between the endosome and the cytosol^15^. Two structural features are conserved among the family members to assist in translocation: the hydrophobic bottleneck ɸ-clamp, and the negatively charged clamps along the β-barrel^9,16–22^. The charged clamps create an electrostatic filter within the pore serving as a charge-state ratchet. Together with the ɸ-clamp, the charged clamps prevent the return of the unfolded A component as it undergoes Brownian diffusion through the pore and deprotonation along the pH gradient. Indeed, mutations in these regions have been characterised to hinder pore maturation and translocation^13,15,23–29^.

While the two-component system of the family is consistent among its well-characterised members, the recently discovered Vpb4 protein subclass challenges this two-component dogma. Unlike archetypal family members, the Vpb4 subclass consists of proteins with no genes for enzymatic components (ADP ribosyltransferase, zinc metalloproteinase, or adenylate cyclase) encoded in the immediate vicinity of the *vpb4* gene^2^. The earliest documented example of toxicity exerted by this subclass is the Vpb4Da2 protein, which was discovered to be active as a single-component pore-forming protein against the important crop pest insect Western Corn Rootworm (WCR, *Diabrotica vergifera vergifera*)^30–32^. The toxicity has been shown through administration of purified protein in an artificial diet^31^ and when expressed in genetically modified plant tissue^32^. Recently, another family member, Vpb4Fa1, was described to have activity in artificial diet assay against the corn pest *Monolepta hieroglyphica* (the two-spotted leaf beetle)^33^. To summarise, two members of the Vpb4 subclass of proteins are functional in killing whole organisms without needing to translocate a secondary component.

There is limited structural information to explain how these Vpb4 proteins function as isolated pores. A current structure reveals that a member of the Vpb4 subclass, pro-Vpb4Da2, adopts a 6-domain topology with N-terminal domains (pro-domain and domains 1-3) structurally conserved with other Bacterial_Exotoxin_B family members^31^. The receptor-binding C-terminal domains (domains 4-6) feature a much higher structural divergence regarding the number of domains or the fold they adopt. Given the roles of these more divergent domains in target specificity, the difference does not explain the ability of the protein to function in a single-component fashion. Thus, there is a knowledge gap in understanding how some of the Vpb4 proteins oligomerise and function as members of the Bacterial_Exotoxin_B family without needing to deliver a secondary component.

In this study, we elucidate the structure of Vpb4Aa2, another member of the Vpb4 subclass, in its pore form. While adopting a similar topology to other Bacterial_Exotoxin_B pores, we highlight in Vpb4Aa2 the absence of structural features important for translocation. Through electrophysiology studies, we show that Vpb4Aa2 pores are capable of causing higher ion flux compared to translocating family member, PA. This renders the Vpb4Aa2 pores distinct from PA, instead displaying characteristics of typical cytolytic pores. These results suggest a molecular mechanism that is unique within the family, consistent with the capability of some members of the Vpb4 subclass to act alone without needing to translocate a secondary factor. Through FoldSeek heuristic search and phylogenetic analysis, we highlight a clade of uncharacterised proteins with the distinguishing features of the Vpb4 subclass.

## Results

### Heptameric pore structure of Vpb4Aa2

Well-studied Bacterial_Exotoxin_B family members normally function by translocating a secondary component for cytotoxicity. Here, we aimed to address how Vpb4 proteins might act as independent pore-forming toxins. While we were unable to express Vpb4Da2 and Vpb4Fa1, we were able to express and purify the close homologue, Vpb4Aa2. Vpb4Aa2 is a Vpb4 member with an overall identity of 53% and 55.6% to Vpb4Da2 and Vpb4Fa1, respectively. Genomically, the *vpb4Aa2* gene is located flanked by two transposases and without a secondary component encoded in its vicinity (Supplementary Fig. 1a).

**Fig. 1:**
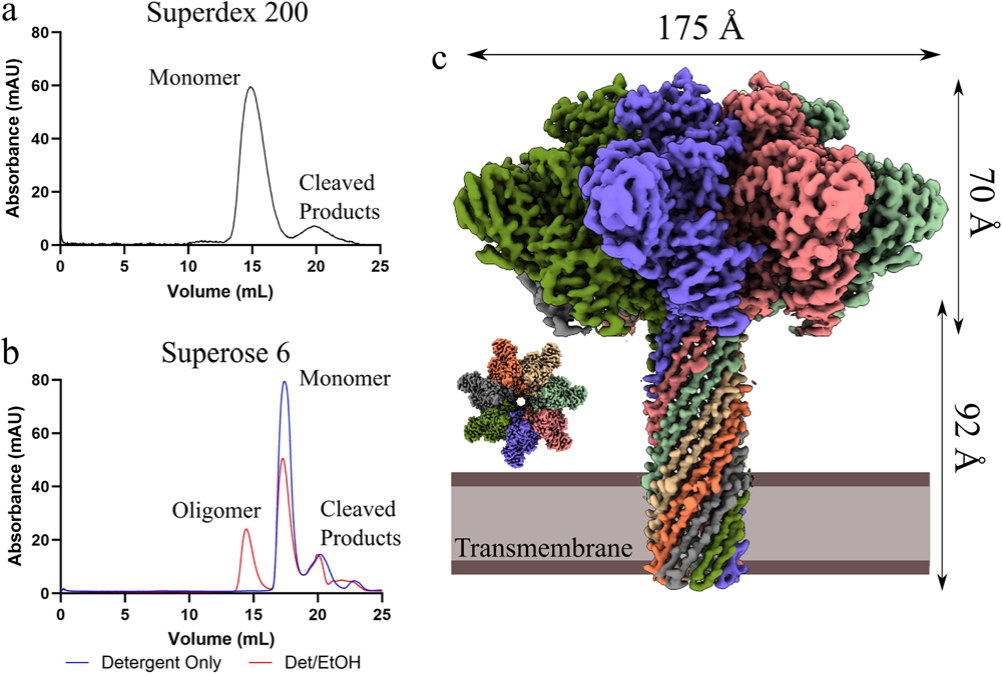
Purification and general analysis of Vpb4Aa2 pores. **a** Size exclusion chromatography profile of Vpb4Aa2 resolved on a Superdex 200 10/300 column. **b** Size exclusion chromatography profile of Vpb4Aa2 with detergent incubation (blue) and detergent/EtOH incubation (red) on Superose 6 10/300 column. **c** Surface map, front and top view of the Vpb4Aa2 pore reconstruction coloured by subunits on top of a membrane with the dimensions.

To isolate the complex of Vpb4Aa2, we produced trypsin-activated Vpb4Aa2 in the presence of LMNG/CHS detergents. Size exclusion chromatography of this preparation revealed that the protein remained monomeric (Fig. 1a) and successful activation was confirmed by a mass shift of ~20 kDa in response to the cleavage of the N-terminal pro-domain at lysine K211 (Supplementary Fig. 1). However, upon concentrating the protein to ~7.5 mg/mL for vitrification, the protein was observed under cryo-EM imaging to have assembled into its oligomeric pore state. We hypothesise that the highly concentrated protein state or the vitrification process facilitated Vpb4Aa2 to oligomerise into pores. Indeed, further analysis by size exclusion and mass photometry (Fig. 1b, Supplementary Fig. 2) revealed that oligomerisation of Vpb4Aa2 could be achieved through ethanol treatment (as previously observed in Iota-B^21^), suggesting that the trypsin-activated material may be metastable and prone to spontaneous pore formation.

We then performed single-particle cryo-EM analysis of the Vpb4Aa2 oligomers, revealing a heptameric pore structure at 2.2 Å with an imposed C7 symmetry in our reconstruction (Fig. 1c). The structure shows higher localised resolution features within the core region of the pore, while the C-terminal extensions are comparatively poorer in resolution (Supplementary Fig. 3). To gain a better understanding of the pore assembly interaction, an atomic model of the Vpb4Aa2 heptamer was determined, encompassing the well-resolved Domains 1-5 of the protein ( ~ 81% of the full-length residues: EMD-71647, PDB: 9PHF, Supplementary Table 1), while Domain 6 of the protein was too poorly resolved for model building. The dimensions of the Vpb4Aa2 pore are 155 Å and 175 Å height and width, respectively, with a 92 Å β-barrel and a 36 Å hydrophobic transmembrane region representing the proposed membrane-spanning portion of β-barrel (Fig. 1c, Supplementary Fig. 3d-f).

### Structure of the conserved pore-forming regions in Vpb4Aa2 and the rest of the family

**Fig. 2:**
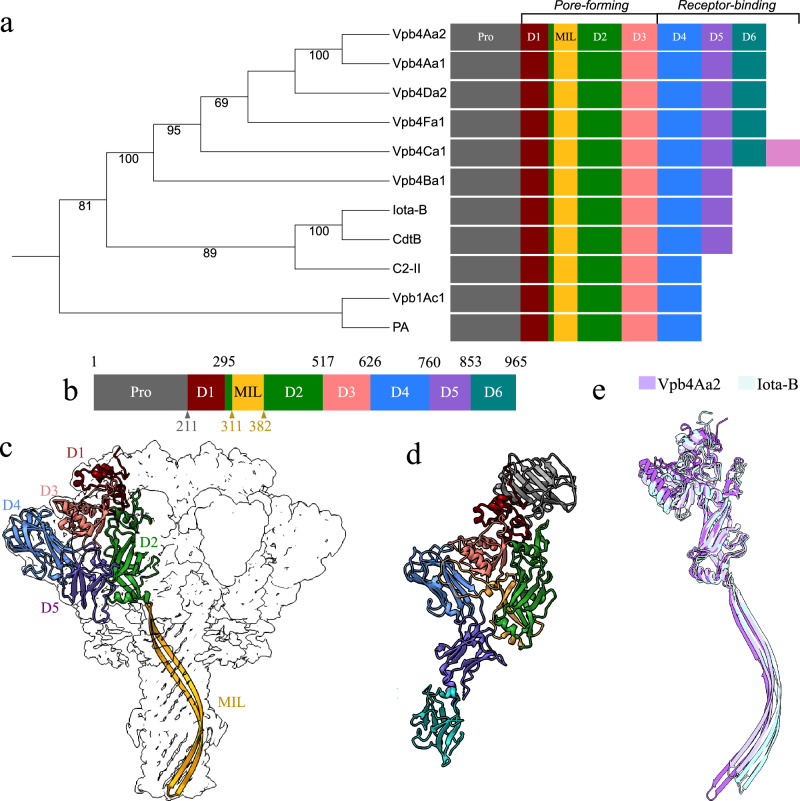
Domain comparison of Vpb4Aa2 to other Vpb4 members and characterised Bacterial_Exotoxin_B family members in pore form. **a** Comparison of the number of domains based on sequences of the proteins. Accession codes are listed in Supplementary Table 5. **b** Domain schematics of Vpb4Aa2 with domain borders and trypsinisation site (grey arrow). The membrane insertion loop (MIL) represents the loop that forms the β-barrel, while the pro-region (Pro) represents the N-terminal region removed during activation. **c** The model of Vpb4Aa2 pore subunit outlined by the density map and coloured by domain. **d** Crystal structure of Vpb4Da2 (PDB ID: 7MJR). The MIL of Vpb4Da2 was predicted based on sequence alignment to be N279–N350. **e** Domains 1-3 superposition of pore-subunits of Vpb4Aa2, PA (6UZB), Iota-B (6KLW), and CdtB (6O2N).

The Bacterial_Exotoxin_B family members follow an identical domain architecture consisting of a pro-domain cap, N-terminal pore-forming domains, and C-terminal receptor binding domain/s (RBD)^4^. This domain composition is also seen in the Vpb4 subclass members, where variation in the RBDs was observed (Fig. 2a–b). Due to a lack of structural data on pro-Vpb4Aa2, we analysed the structural rearrangement of the Vpb4Aa2 pore in comparison to the related protein pro-Vpb4Da2 (53% overall sequence identity, same domain composition). The majority of pore-forming domains (PFDs, Domains 1-3) remain predominantly unchanged when comparing the monomeric and pore forms of Vpb4 (Fig. 2c–d). The calcium-binding region at Domain 1 is conserved amongst the two Vpb4 subclasses, and other related toxins, PA, CdtB, and Iota-B (Supplementary Fig. 4). Two major rearrangements on the PFD were observed upon pore formation: the loss of the pro-domain and the unravelling of the membrane-insertion loop (MIL) to form the β-barrel.

We then analysed the structure of the Vpb4Aa2 pore with other pore structures of the Bacterial_Exotoxin_B family (PA, Iota-B, and CdtB) (Fig. 2e). The overall architecture and fold of the PFDs of Vpb4Aa2 remain consistent amongst the Bacterial_Exotoxin_B family (average RMSD = 1.188 Å). Given the similarity, we hypothesise that the Vpb4 subclass undergoes a shared mechanism of pore formation with the rest of the family members. Indeed, interface analysis using PDBePISA^34,35^ revealed that these domains are responsible for stabilising the pore complex, with domain 2 as the major contributor to these subunit interactions (Supplementary Table 2). The average subunit-subunit buried surface area was estimated to be 3465.3 Å^2^ with a ΔG value of −8.5 kcal/mol, consistent with other family members (Supplementary Table 3).

### Analysis of the Vpb4Aa2 pore lumen

To date, the Bacterial_Exotoxin_B family members (PA, Iota-B, and CdtB) share common structural features along the pore lumen to allow translocation of a secondary proteinaceous toxin. These include the clamp-like features at the cap of the funnel region (α-clamp or Ca-edges), the entrance of the β-barrel (bottleneck ɸ-clamp), and along the β-barrel (negatively charged clamps)^16–22^. Given that two members of the Vpb4 subclass function as independent cytolytic pores, we analysed the pore lumen interface of Vpb4Aa2 for key differences in these structural features that may explain this ability.

In terms of secondary toxin binding at the funnel region, the Vpb4Aa2 pore was observed to retain the dual-calcium-binding region at Domain 1 deemed important for binding toxins for translocation amongst the family members (Supplementary Fig. 4)^17,19,21^. In comparison to PA, the residues of the α-clamp involved in LF binding are not conserved in Vpb4Aa2 (Supplementary Fig. 5), except for F273 (equivalent to F236 of PA). In comparison to CdtA/CdtB and Iota-A/Iota-B binary toxin systems, the NSS/NSQ residues in the loop previously characterised to clamp the enzymatic partners were not conserved in Vpb4Aa2 (Supplementary Fig. 5). Thus, although Vpb4Aa2 conserves the Domain 1 calcium binding regions, the capability of the protein to dock any secondary toxins of the current characterised systems (PA, CdtB, and Iota-B) remains a question due to the lack of α-clamp specific residues and the NSS/NSQ loop.

**Fig. 3:**
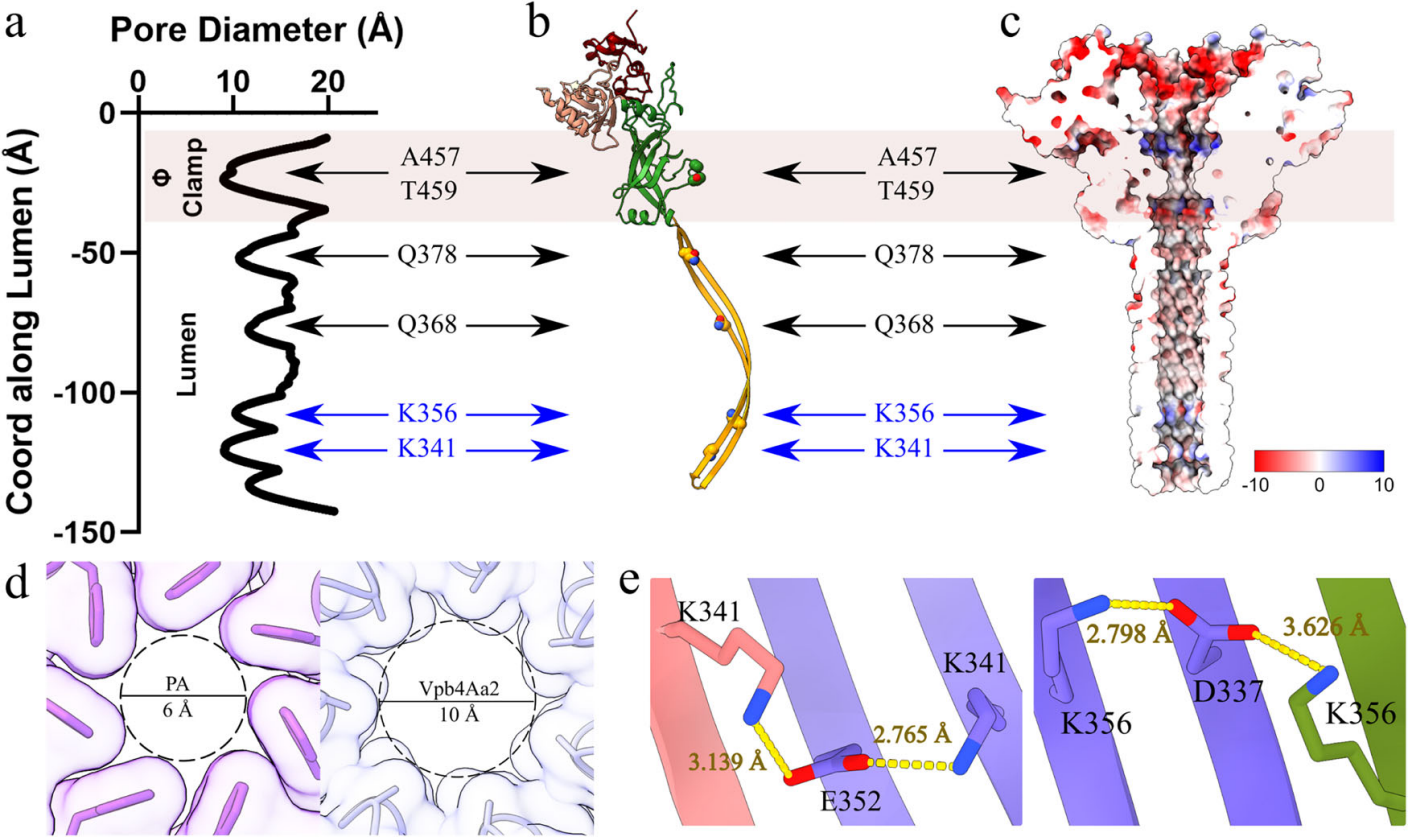
Constriction analysis of the Vpb4Aa2 pore lumen. **a** Hole2 analysis of the Vpb4Aa2 pore lumen marked by residues. Location of ɸ-clamp is highlighted by the red shade. **b** Constriction site on the Vpb4Aa2 model. **c** Cross-section of Vpb4Aa2 pore in its electrostatic potential surface representation. Colour key is provided for the colouring of the surface. **d** ɸ-clamp of PA (6UZB) and Vpb4Aa2 and their diameter. **e** Charge complementary pairings on the Vpb4Aa2 β-barrel.

The diameter analysis along the Vpb4Aa2 pore lumen revealed six clamp-like regions with luminal diameters between 9 and 21 Å (Fig. 3a–c). The first and arguably the most important clamp is a 10 Å gate at the entrance that corresponds with the bottleneck ɸ-clamp of the Bacterial_Exotoxin_B family (residues A457 and T459). Interestingly, the ɸ-clamp in Vpb4Aa2 is formed by a ring of alanine residues (A457), rather than the canonical 6 Å ring of phenylalanine residues as seen in PA, CdtB, and Iota-B (Fig. 3d, Supplementary Fig. 6a–d). Accordingly, this substitution results in a 4 Å widening of the ɸ-clamp. While there is a slight narrowing (9.2 Å-wide) constituted by threonine residues (T459) below the ɸ-clamp region in Vpb4Aa2 (Supplementary Fig. 6a), the overall lumen analysis suggests that Vpb4Aa2 forms a pore with wider structural bottlenecks compared to the rest of the structurally characterised family members. Furthermore, the structurally conserved aspartate (D425 in PA) residue critical for ɸ-clamp formation and pore maturation is replaced by asparagine (N455 in Vpb4Aa2)^24^. Overall, Vpb4Aa2 has a ɸ-clamp landscape that is widely distinct from the rest of the family members.

Along the β-barrel, the next two constrictions in the Vpb4Aa2 pore, composed of glutamine residues (Q378 and Q368), tighten the lumen to ~11.5 Å in diameter. The last two constrictions at the transmembrane region, composed of lysine residues (K356 and K341), tighten the lumen to ~11 Å and ~9 Å respectively. The Vpb4Aa2 pore does not contain the canonical negatively charged clamps that have been observed in the pore lumen of PA, Iota-B, and CdtB (Fig. 3c, Supplementary Fig. 7). While acidic residues do exist facing the lumen (D337 and E352), they are positioned adjacent to the basic lysine residues (K356 and K341) (Fig. 3e), where these residues were shown to be within distance to form salt bridges. These acidic and basic residues provide complementary charge pairs unique to Vpb4Aa2, akin to the presence of charge complementary pairings observed in other lytic β-barrel proteins, aerolysin and alpha-hemolysin (Supplementary Fig. 7).

When comparing Vpb4Aa2 with the other members of the Vpb4 subclass, features of the pore lumen are highly conserved. The ɸ-clamp landscape and the pore-forming loop were shown to be consistent in a sequence-level analysis (Supplementary Fig. 6, 8a–b), with the exception of Vpb4Ba1 as an outlier. Indeed, comparison to the two characterised active family members, Vpb4Da2 and Vpb4Fa1, revealed increased identity in the functional PFD (domain 1-3), sharing 68% and 77% sequence identity, respectively (83.3% and 86.7% similarity). In comparison, the receptor binding domains (domain 4-6) of Vpb4Aa2 only share 42.9% and 35.7% identity to Vpb4Da2 and Vpb4Fa1, respectively. The identity is particularly reflected in the residues at the pore lumen of Vpb4Aa2 (coloured based on sequence conservation between the three proteins; Supplementary Fig. 8c), suggesting a shared mechanism among the Vpb4 subclass.

### C-terminal receptor binding domains of Vpb4Aa2

The archetypal Exotoxin B protein PA has 4 structural domains that are seen across members of this family (such as Vpb1Ac1 and C2-II). Other members of the family show variation in the number of additional domains at the C-terminus such as Iota-B and CdtB having one extra domain, and Vpb4Aa2 and Vpb4Da2 having a further structural domain 6 (Fig. 2a). Given that a 6-domain toxin has not been characterised in its pore state, we next sought to understand the role of these RBDs and their rearrangement upon pore formation of Vpb4Aa2.

**Fig. 4:**
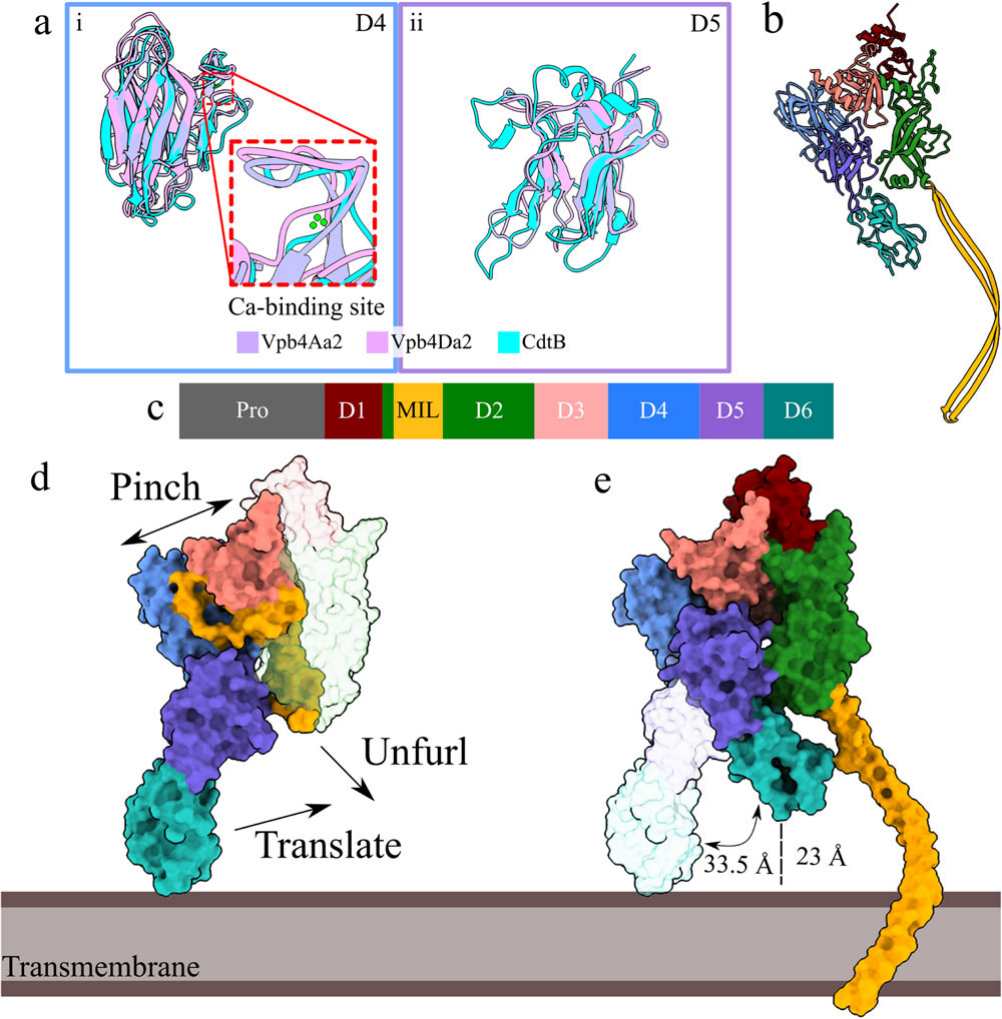
Receptor-binding domain relocation of full-length Vpb4Aa2 upon pore formation. **a** Superposition of (i) domain 4 and (ii) domain 5 of Vpb4Aa2 against Vpb4Da2 (7MJR), and CdtB (7VNN). Inset revealed location of calcium on Domain 4 outlined in red. Outer box represents the domain (D4 outlined in cornflour blue and D5 outlined in slate blue). **b** The model of full-length Vpb4Aa2 pore subunit. **c** Domain topology schematics of Vpb4Aa2. **d** Three major movements highlighted on pro-Vpb4Da2 (as subclass representative) before pore formation. **e** Full-length Vpb4Aa2 pore subunit showing translation distance and approximate distance between domain 6 and the membrane.

Our initial model consisted of domain 4 and 5 of the RBD. The fold of domain 4 in Vpb4Aa2 is consistent with Vpb4Da2, CdtB, and Iota-B; but not PA (Fig. 4a). This includes the presence of calcium ions bound and the coordinating residues (Supplementary Fig. 3a, Supplementary Fig. 9a). As calcium depletion of D4 has been reported as a trigger for pore-formation on CdtB^12^, a C1 reconstruction of the pore model was initiated to confirm whether calcium was present on each subunit. The reconstruction at a global 2.3 Å resolution reveals density for calcium ions on each subunit, suggesting a different mechanism of pore-formation by Vpb4Aa2 (EMD-68793, Supplementary Fig. 9b).

The fold of domain 5 is also consistent to Vpb4Da2 and CdtB (Fig. 4a) with an RMSD of 0.97 and 1.08 Å respectively. Fold search using DALI revealed similarity to fungal immunomodulatory protein family members (FIP-Fve^36^ and FIP-Nha^37^), sharing a carbohydrate-binding module (CBM)-34 fold (average RMSD = 0.96 Å)^38^. Domain 6 of the RBD, unfortunately, was still too poorly resolved for modelling. Instead, we made an additional model where AlphaFold was used to predict the domain fold and molecular docking was performed to highlight the possible rearrangement occurring during pore formation (Fig. 4B, PDB: 22ZO, Supplementary Fig. 9c).

We next characterised the movement of the C-terminal domains of the Vpb4Aa2 pore with respect to the structure of pro-Vpb4Da2, which represents a related family member in its pro-form. Here, domain 5 and 6 of the RBDs were observed to undergo a twisting motion, tightening towards the β-barrel (Supplementary Movie 1). This resulted in a ~ 33.5 Å translation of the C-terminal receptor binding domains compared to the pro-form (Fig. 4c–e). We hypothesise that this translation occurs in conjunction with the unravelling of the MIL, which is initially wedged between Domains 3 and 4. Upon this domain rearrangement, the most distal residues of Domain 6 were observed to be ~23 Å above the predicted membrane-inserting region, suggesting the approximate height of receptor recognition for target tissue. Taken together, our model suggests that the movement of the receptor binding domains may be interconnected with pore formation.

### Activity of the Vpb4Aa2 protein against cultured cells

Insecticidal activity has been demonstrated for Vpb4Da2 against Western corn rootworm and Vpb4Fa1 against the two-spotted leaf beetle^31,33^. Since none of the collaborating laboratories has access to these insects (for quarantine reasons), a preliminary screen of Vpb4Aa2 against cultured cells from Western corn rootworm (and others) was undertaken (Supplementary Fig. 10). However, no significant effects of Vpb4Aa2 were seen with these insect cells. To further probe for activity, we assessed the capacity of the protein to form pores in artificial membranes.

### Pore-forming ability of Vpb4Aa2 in TIRF Ca^2+^ flux system

**Fig. 5:**
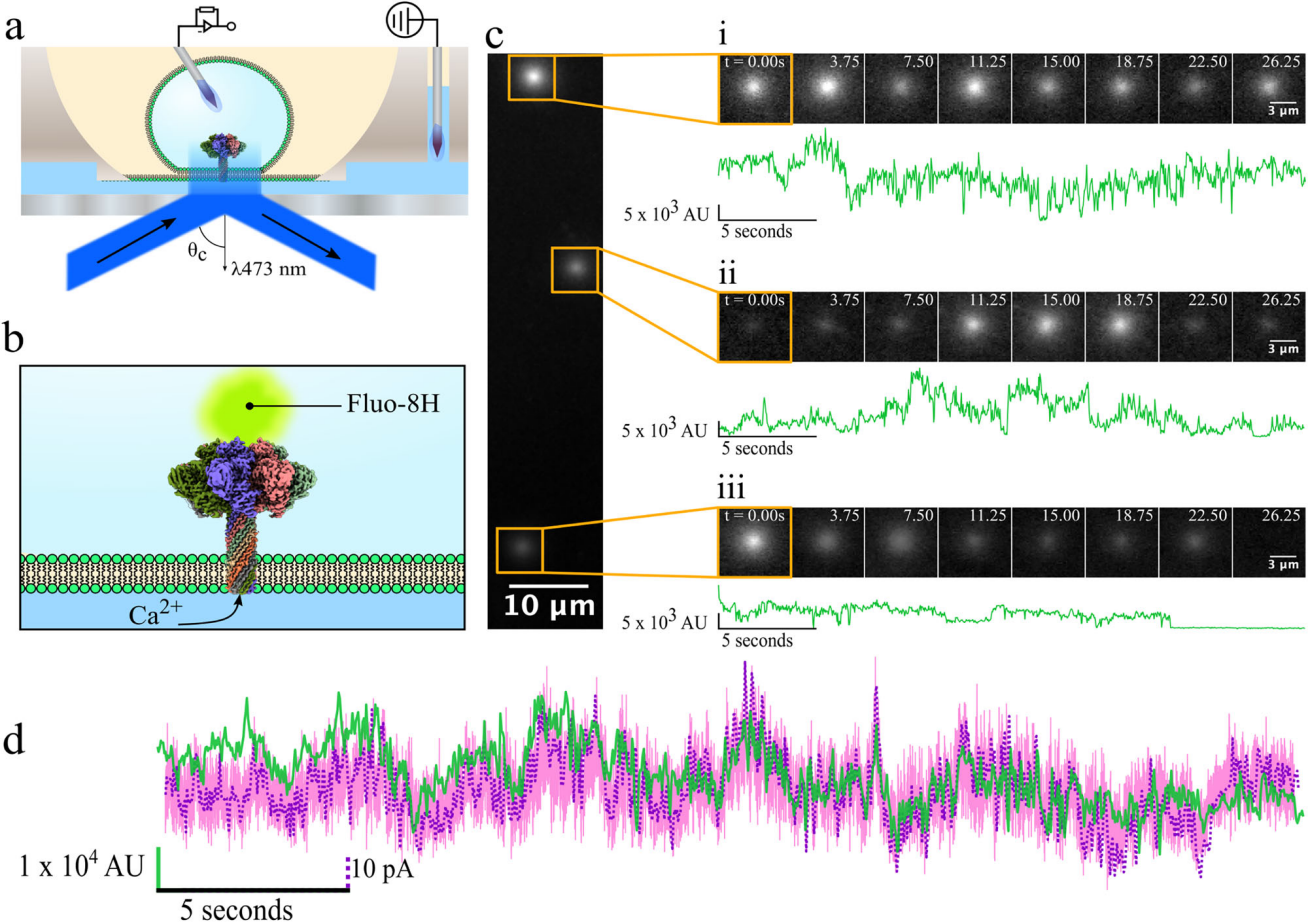
Direct imaging of functional Vpb4Aa2 pores in artificial bilayers. **a** Experimental setup for imaging Ca^2+^ flux through Vpb4Aa2 pores in a hydrogel supported droplet interface bilayer via Total Internal Reflection Fluorescence (TIRF) microscopy. **b** Ca^2+^ flux through Vpb4Aa2 pores binds the calcium sensitive fluorescent dye, Fluo-8H, on the opposing side of the membrane, allowing for visualisation of individual pore function. **c** A portion of the imaged bilayer showing three functional Vpb4Aa2 pores (visualised as a median pixel-projection of a 30 s measurement with 30 ms frame time). (i-iii) Time-course Ca^2+^ flux imaging of each of the three Vpb4Aa2 pores displayed as fluorescent image montage showing Ca^2+^ flux response at 3.75 s intervals, and quantified fluorescence over time (green trace), corresponding to Ca2+ flux (30 ms frame time). **d** The summed fluorescence time-course signal of the three imaged pores (green) shows close agreement with simultaneously measured electrophysiology current (purple) as indicator for total ion flux across the bilayer (light-purple represents raw electrophysiology measurement, dark-purple overlay represents electrophysiology data binned to equivalent sampling rate as the image acquisition).

To verify that Vpb4Aa2 possessed pore-forming ability in artificial membrane systems, we utilised single-molecule imaging via Total Internal Reflection Fluorescence (TIRF) microscopy to measure Ca^2+^ flux^39^ through individual pores in hydrogel-supported droplet interface bilayers (DIBs)^40^ (Fig. 5a). The bilayers are formed by contacting two lipid monolayers, spontaneously self-assembled at water-oil interfaces. In this case, an aqueous droplet is brought into contact with a spin-coated hydrogel layer on a glass coverslip, in an oil environment with dissolved lipid, creating a lipid bilayer amenable to optical access and simultaneous electrophysiology. Ca^2+^ transit from the underlying aqueous hydrogel through individual Vpb4Aa2 pores is imaged directly via the Ca^2+^ sensitive fluorescent indicator, Fluo-8, present in the aqueous droplet (Fig. 5b). Here, the Ca^2+^ flux imaging revealed the ability of Vpb4Aa2 to form pores in artificial lipid bilayers. Figure 5 shows the fluorescence signal by Ca^2+^ flux through three individual Vpb4Aa2 pores (Fig. 5c and Supplementary Movie 2). Quantitative fluorescence imaging allows characterisation of each pore and its contribution to the overall ion flux, with the summed fluorescence mirroring the electrophysiology current (Fig. 5d). Having established the pore-forming activity of the protein, a more detailed electrophysiological study was initiated.

### Electrophysiological characterisation of Vpb4Aa2 in DIB artificial membrane

**Fig. 6:**
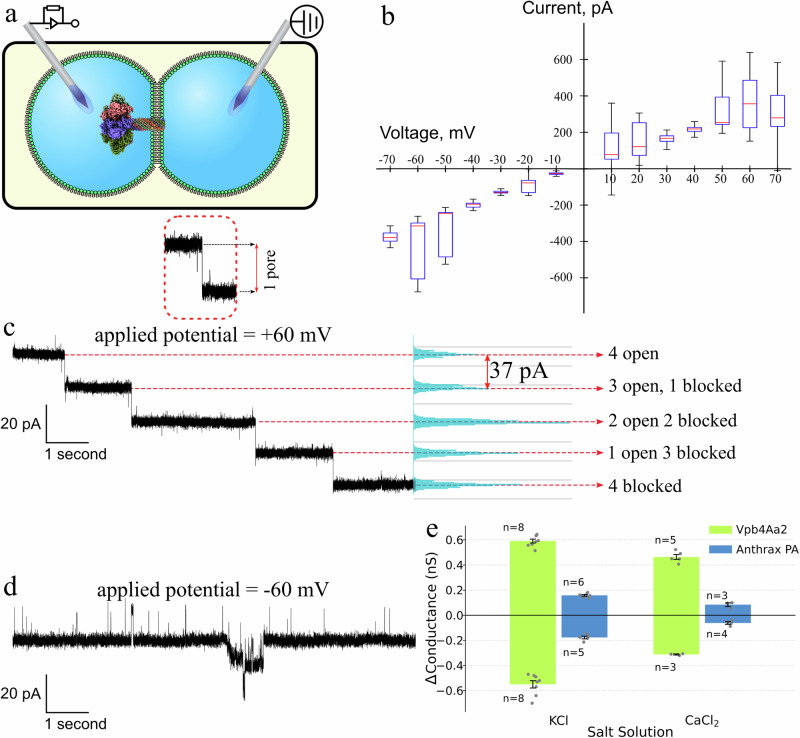
Electrophysiological behaviour of Vpb4Aa2 in droplet interface bilayers and comparison against PA. **a** Experimental setup for electrophysiology in droplet interface bilayers (DIBs). Both Vpb4Aa2 and PA spontaneously self-assemble and insert into the bilayer. **b** The current/voltage (I/V) relationship of Vpb4Aa2 in a single bilayer over −70 to +70 mV in 10 mV increments, alternating between negative and positive potential over 4 repeated cycles. Each voltage increment consisted of 130196 measurements (13 s). Based on a single pore conductance of ~37 pA and a median current of 357 pA at 60 mV, 10 pores were estimated to be measured in each sweep. The centre line of each box (red) represents the median, the bounds of the box (blue) represent the interquartile range, and the whiskers are the most extreme value not considered to be an outlier. Outlier is determined by being >75th percentile + (1.5 * IQR) or <25th percentile - (1.5 * IQR). **c** Exemplar current traces showing the conductance behaviour of Vpb4Aa2 under +60 mV revealed step-wise current changes corresponding to conductance of single pores ( ~ 37 pA). The inset histogram demonstrates consistent step heights between current states corresponding to individual Vpb4Aa2 pores. **d** Current traces under applied −60 mV revealed variability in behaviours with transient upward spikes indicative of short-lived blocked states at negative potential. **e** Conductance comparisons between individual Vpb4Aa2 and PA pores in single bilayers bathed either in KCl or CaCl_2_. Minima and maxima of the bars represent the mean change in conductance observed during pore opening/closing events with bar sign (positive or negative) indicating the applied potential for each salt condition. Individual observed changes in conductance are displayed as grey filled circles. *N* for each condition, indicating the number of insertion events, is given on the bar. Error bars represent standard deviation.

Following successful confirmation that Vpb4Aa2 can form bona fide membrane-spanning pores, we performed electrophysiology experiments in a droplet interface bilayer (DIB) artificial membrane (Fig. 6a)^41^. In this system, 1 M KCl was used to provide charge carrier ions (K^+^ and Cl^-^), while the activated protein was added on one side of the droplet bilayer to ensure a single orientation of insertion into the membrane (Fig. 6a). This same side of the membrane contained the driving electrode, while the opposite side contained the ground electrode. Based on this orientation, the voltage applied across the membrane is referenced as the potential difference between the β-barrel stem opening and the larger cap end of the Vpb4Aa2 pore.

A potential difference was applied across the otherwise impermeable membrane and a current, indicative of ionic flux across the bilayer through the Vpb4Aa2 pores, was measured. Current measurements under applied potentials between -70 mV and +70 mV showed ionic conductance under both negative and positive applied potentials (Fig. 6b). The observed conductance scaled approximately in proportion to the applied potential, demonstrating the largely non-rectifying nature of the Vpb4Aa2 pore (i.e., no de facto strong ionic current directional preference or voltage dependency).

At higher positive applied voltages ( + 50 to +70 mV), however, a plateauing of current was observed in the I/V curve (Fig. 6b). An explanation for this is given on inspection of the electrophysiology time-course traces (Supplementary Fig. 11a). The traces showed a tendency for stochastic Vpb4Aa2 pore block at higher positive potentials, characterised by the stepwise decreases in current over time as individual pores stochastically block. Unblocking events, characterised by an equivalent magnitude step increase in current, are infrequently observed. This gave rise to a net effect of an attritional decrease in current over time under higher positive applied potentials, as observed in the time-course trace at +60 mV (Fig. 6c). Reversing the applied potential resulted in channel unblocking and recovery of current, evidencing continued presence of functional pores.

Comparable pore blockage was not observed at negative potentials (Fig. 6d and Supplementary Fig. 11a). Instead, transient (ms timescale) gating-like events were observed at applied voltages of −60 mV (Fig. 6d). These gating-like events are represented as brief upward spikes, which correspond to short-lived declines in current consistent with transient pore block. Traces under lower negative applied potential, however, do not share the transient pore longer-timescale blocking events.

### Electrophysiological comparison between Vpb4Aa2 and PA

Given that the Vpb4 subclass is unique regarding its independent killing mechanism, we then performed a comparative analysis on the electrophysiological characteristics of Vpb4Aa2 as a membrane-disrupting pore in contrast to the related toxin PA as a translocase. Analysis of the pore conductance in KCl and CaCl_2_ environments revealed higher conductance of Vpb4Aa2 for both monovalent K^+^ and divalent Ca^2+^ carriers and under both positive and negative applied potentials (Fig. 6e and Supplementary Table 4). This much larger conductance of Vpb4Aa2 is indicative of greater ion flux through the pore, suggesting that wider ɸ-clamp, the overall neutrality, and/or the charge complementarity of the β-barrel facilitate greater ionic transport across the membrane.

At the single channel level, flickering increases in PA current, previously ascribed to changes in ɸ-clamp occupancy and dilation^14,42^, were observed as frequent transient spikes to higher current states on the millisecond-timescale (Supplementary Fig. 11c). In contrast, such behaviour is largely absent in Vpb4Aa2 (Fig. 6c and Supplementary Fig. 11ab). High frequency transient reductions in current (channel block) are seen in PA, in an otherwise persistent open-state, attributed to pore structural flexibility^43^ and changes in ɸ-clamp occupancy^44^. Similar behaviour, albeit at lower frequency, was only observed in Vpb4Aa2 under higher negative applied potentials (−60 mV) (Fig. 6d). Unlike PA, longer time-scale block was observed in Vpb4Aa2 under positive potentials (Fig. 6c) These differences are likely a result of the reduced influence of the Vpb4Aa2 ɸ-clamp, as alanine substitution in the PA ɸ-clamp (PA_63_ F427A) notably result in comparable changes in behaviour^44,45^, along with an increase in channel conductance^45^, reduction in cation selectivity^46^, and abolition of translocation function^28^. Consequently, it is likely that the differences in the ɸ-clamp between Vpb4Aa2 and Anthrax PA significantly impacts channel behaviour, contributing to increased conductance and reduced selectivity in Vpb4Aa2, conferring properties well suited to a non-specific ion-release channel. Taken together, these results suggest that despite the large degree of structural similarity with other members of the Bacterial_Exotoxin_B family, Vpb4Aa2 can function as a bona fide pore causing ionic leakage as a possible mechanism of action, which remains to be further investigated.

### Prediction of Vpb4-like proteins within the family

To explore the structural landscape of the Vpb4 subclass within the Bacterial_Exotoxin_B family, we queried the N-terminal domains of Vpb4Aa2 (Pro-domain and D1-3, residues 41-626) to look for proteins with homologous folds within the Protein DataBank (PDB) and AlphaFold Protein Structure Database (AFDB). Searches of the PDB returned hits on the characterised family members CdtB, Iota-B, PA, and C2-II as expected. In contrast, searches of the AFDB, revealed numerous uncharacterised members with varying degrees of sequence and fold similarity.

**Fig. 7:**
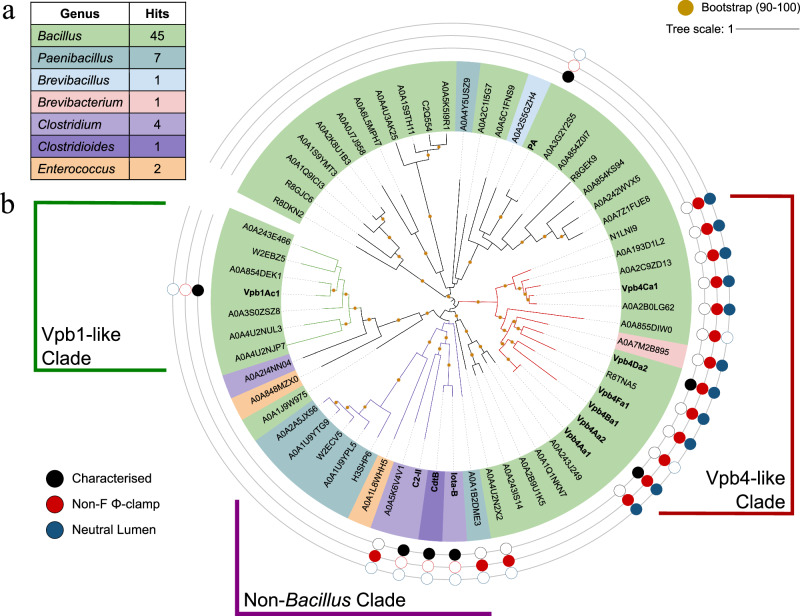
Phylogenetic analysis of putative Bacterial_Exotoxin_B family members based on FoldSeek-AFDB hits. **a** Spread of the structurally characterised family members (PA, CdtB, Iota-B, C2-II, and Vpb1Ac1), Vpb4 subclass members, and top 50 AFDB hits among the genera. **b** Phylogenetic tree highlighting Vpb4-like clade, Vpb1-like clade, and a clade of binary toxins outside of the Bacillus genus. Tree is annotated based on the protein characterisation status and the features of the respective lumen (where no circles are depicted, the analysis has not been performed).

To characterise the Vpb4 subclass further, we performed phylogenetic analysis of AFDB hits with probability > 0.8, TM-score > 0.6, and individual pLDDT > 0.7. The AFDB hits consist of 50 proteins originating from 31 unique species/strains from the genera *Bacillus, Paenibacillus, Brevibacillus, Clostridium, Clostridioides, Enterococcus*, and *Brevibacterium* (Fig. 7a). A phylogenetic tree was then constructed using these AFDB hits, together with 6 members of the Vpb4 subclass taken from the Bacterial Pesticidal Protein Resource Centre (BPPRC) (Vpb4Aa1, Vpb4Aa2, Vpb4Ba1, Vpb4Ca1, Vpb4Da2, and Vpb4Fa1), other structurally relevant family members (PA, CdtB, Iota-B, and C2-II), and Vpb1Ac1 as representative of the Vpb1 subclass (Fig. 7b).

Phylogenetic reconstruction of the Bacterial_Exotoxin_B family revealed a distinct clade of Vpb4-like proteins separate from the PA, CdtB, Iota-B, C2-II, and Vpb1Ac1 with high confidence as observed by the bootstrap value (Fig. 7b). This Vpb4-like clade comprises all previously recognised Vpb4 subclass members, seven uncharacterised *Bacillus* proteins, and a single protein from *Brevibacterium* (Fig. 7b). Interestingly, sequence-level analysis of the members of the Vpb4 clade showed that 13 members had a consistent lack of the canonical phenylalanine ɸ-clamp with either missing residues or mutation to residues with side chains sterically smaller than phenylalanine (alanine, serine, threonine, and glutamine). Furthermore, AlphaFold-predicted pore structures revealed an overall neutral charged lumen and charge complementarity, except for Vpb4Ba1 and A0A855DIW0 (Supplementary Fig. 12). Notably, two uncharacterised proteins (A0A193D1L2 and A0A2C9ZD13) in the Vpb4 clade also originate from *B. thuringiensis* strains and thus are putative Vpb4 members.

In the broader tree topology, the Vpb4-like clade was observed to diverge early from a common ancestor with the binary Vpb1-clade and non-*Bacillus* clade. Three other proteins from the genera *Bacillus*, *Clostridium*, and *Paenibacillus* (A0A4U2N2X2, A0A5K6V4V1, and A0A1B2DME3) also exhibit the phenylalanine substitution of the ɸ-clamp, but each also contains negatively charged residues in its lumen (Supplementary Fig. 12). Among them, A0A5K6V4V1 was designated to be BECb, a less studied non-canonical Bacterial_Exotoxin_B family member with a serine ɸ-clamp. BECb was shown to be functional as a translocase for the ADP-ribsoylating BECa protein in cell assay, but was also able to act independently to cause fluid accumulation in suckling mouse assay when administered independently^47,48^. In this regard, the presence of complementary charged residues in the lumen and missing encoded enzymatic components to Vpb4 proteins could potentially define the Vpb4 subclass.

## Discussion

The Western Corn Rootworm (WCR, *D. virgifera virgifera*), also known as the billion-dollar bug, is one of the most voracious insect pests infesting corn crops in North America^49^. The two-spotted leaf beetle (*M. hieroglyphica*) is another important pest targeting broad host crops in East and Southeast Asia, particularly in China^50^. Given the rising resistance against currently deployed insecticides^51^, there is a pressing need to develop more pesticides (including pesticidal pore forming proteins) with distinct modes of action as additional tools for pest management. One of the emerging alternatives is the Vpb4Da2 protein, a Bt toxin discovered to be active against WCR with no cross-resistance with the currently deployed proteins (Cry3 and Gpp34/Tpp35)^30–32^; and Vpb4Fa1, a Bt toxin recently discovered active against *M. hieroglyphica*. The Vpb4 subclass members (to which Vpb4Da2 and Vpb4Fa1 proteins belong) are encoded as isolated proteins and are functional in and of themselves, unlike the canonical members from the Bacterial_Exotoxin_B family^3^. To understand the mechanism of the Vpb4 proteins, we performed biochemical characterisations of a member of the Vpb4 subclass, Vpb4Aa2.

In this study, we determined the pore structure of Vpb4Aa2. While the protein specificity has yet to be determined against any targets (invertebrate or otherwise), the identical fold of the N-terminal PFD (domains 1-3) between the protein and Vpb4Da2, combined with the very close phylogenetic relationship with Vpb4Da2 and Vpb4Fa1, suggests analogous function in terms of pore-formation and oligomerisation. Akin to the rest of the Bacterial_Exotoxin_B family members, Vpb4Aa2 undergoes proteolytic removal (at K211 according to Edman Degradation) of the ~20 kDa pro-domain to form functional pores^3^. We found that the Vpb4Aa2 protein is also consistent with other family members regarding pore formation and oligomerisation in other respects. Features, such as the dual-calcium-binding site at Domain 1 (and another calcium-binding-site at Domain 4 for some members), unravelling of the MIL at Domain 2, and the formation of a heptameric complex are well-conserved among the family members^16,18–22^. The Vpb4Aa2 structure also represents a 6-domain Bacterial_Exotoxin_B family member in its pore state.

From a functional perspective, the most defining feature of the two members of Vpb4 subclass (Vpb4Fa1 and Vpb4Da2) is the ability to kill without having a secondary component. While cytotoxicity of canonical Bacterial_Exotoxin_B members in the absence of their cognate A component has been observed at a cellular level in CdtB^10,11^, Iota-B^52^, and PA^53^, administration of purified protein has not been shown to result in lethality in animal models^54–56^. Interestingly, a recent study by Simpson et al. revealed that a *C. difficile* strain with the *cdtB* gene, but not the *cdtA* gene, increases the virulence level in comparison to strain without the two genes in a hamster model, but not in a mouse model^57^. Notably, administration of purified CdtB protein was not performed in the hamster model. Given that two different forms of Vpb4 have been shown to kill whole organisms as purified proteins, we hypothesised that the Vpb4 subclass may function through different mechanisms of killing compared to PA, CdtB, and Iota-B.

To understand this distinct mechanism further, we looked into whether the Vpb4Aa2 pores exhibit key structural features for translocation. The dual-calcium-binding site at Domain 1 itself is involved in cargo toxin binding^17,19,21^. While the capability to bind a secondary component remains a question, our model reveals that key components for translocation are missing in Vpb4Aa2. The first distinguishing feature is the lack of negatively charged clamps, which are thought to assist translocation through deprotonation or microenvironments that help refold the cargo proteins^13,22^. Instead, the Vpb4Aa2 pore model reveals a neutral lumen with the two charged side chains occupied by complementary pairing in the transmembrane lumen.

The other critical feature of Vpb4Aa2 is the presence of an alanine ɸ-clamp rather than the canonical phenylalanine critical for translocation. In PA, mutation from phenylalanine to alanine of the clamp abolished translocation, as the hydrophobic bottleneck composed of the phenylalanine is rationalised to be essential for preventing retro-translocation and producing directional bias^58^. Furthermore, the lack of a functional equivalent of an aspartate two residues upstream of the ɸ-clamp (D425 in PA) also hints at the inability to translocate^24,27^. Changes in any of these regions are associated with failure in translocation, whilst retaining the ability to form pores with increased channel conductance and reduced ion selectivity^13,25–29^. Here, we reason that this assemblage of distinct features in the Vpb4Aa2 pore, associated with loss of translocase function in archetypal family members, contribute to a different mechanism of action. This reconciles previously reported observations of the ability of Vpb4 sub-family members to kill at organism level without a partner toxin. Thus, the Vpb4Aa2 pore lumen is illustrative of other Vpb4 members and provides insights into how these proteins might function as simple lytic pore-forming proteins rather than translocases.

The Vpb4Aa2 cell-based assays performed against WCR cells did not produce evidence of activity, which may reflect that the source insects are refractory or that the required receptors are not present in these cell lines. Given the low identity of the receptor binding domains, this is not surprising, given that insecticidal toxins tend to have narrow target ranges. To further our understanding of the Vpb4Aa2 function as a pore, electrophysiology experiments were undertaken. Our preliminary electrophysiology study revealed a pore with prolonged open characteristics, indicative of a pore with largely fixed luminal conductance and a stable open state. The conductance of our pores is greater than PA and less ion selective, with comparable properties to those of PA mutants with alanine substitution in the ɸ-clamp^44–46^. Regarding the charge profile within the lumen, the charge complementary pairings on the β-barrel resemble those of cytolytic pore-forming proteins, such as aerolysin^59^ and Alpha-hemolysin^60^. While complementary pairs effects in this system have not been extensively studied, a recent paper by Mayer et al.^61^ suggested that luminal charge and the presence of complementary pairing play major roles in gating and passaging of ions across the β-barrel. Here, we hypothesise that both the loss of the bottleneck and the charge profile of the pore lumen are critical in the independent function of the Vpb4Aa2 subclass.

As we isolated the Vpb4Aa2 pore and demonstrated perforation of the membrane in DIB at neutral pH, we hypothesise that the protein may be able to insert into the membrane at this pH state in the physiological context. This is contrary to PA, where the low pH during endocytosis drives pore formation^58^. Based on the calcium density that we observe at domain 4 in each subunit of our model, we also hypothesised that the pore formation of Vpb4 is not driven by a calcium depletion event in the endosome as previously reported in CdtB^12^. Given these observations, together with the loss of structural features required for translocation and lack of an encoded A component, we further hypothesised that pore formation for Vpb4Aa2 may occur at the cell surface, rather than requiring endocytosis and acidification. Whether endocytosis is involved during Vpb4Aa2 activity in vivo is currently unknown.

**Fig. 8:**
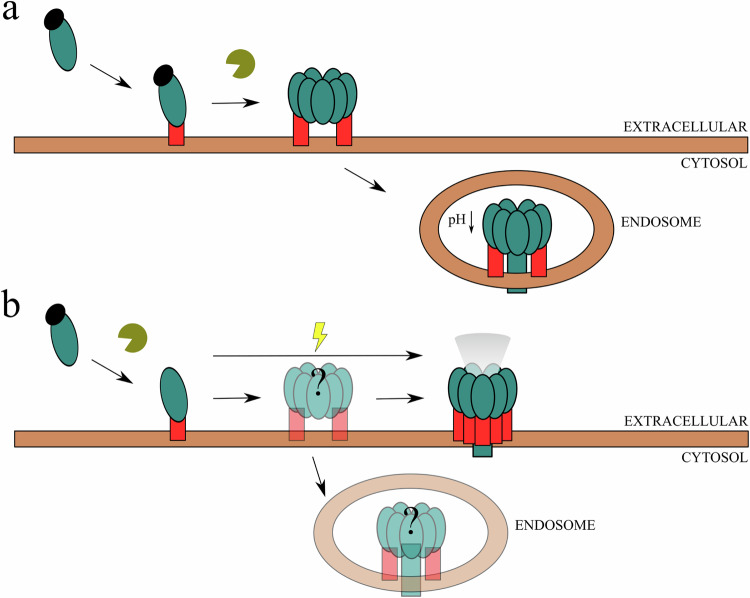
Proposed pore-forming pathway of the Vpb4 protein compared to PA. **a** Proposed pathway of PA involves receptor binding and activation, pre-pore formation, and endocytosis, as membrane perforation is driven by low pH. **b** Proposed pathway of the Vpb4 protein involves receptor binding to increase effective concentration and immediate pore formation. Whether intermediary states exist or maturation in the endosome is necessary within the oligomerisation/perforation process remains unsolved.

While the trigger for pore formation remains a question, here we hypothesised that concentration is involved in oligomerisation. Given that our method to obtain the complex involves enriching the protein to a high concentration (7.5 mg/mL), we propose that at high concentration, each Vpb4 subunit is more likely to interact with each other and oligomerise. In the context of the insect guts, the presence of receptors may contribute by orienting the activated subunits in proximity to initiate the oligomerisation process or to help reduce the energy threshold for oligomerisation to occur. According to the substantial domain rearrangement of the C-terminal domains as a pore complex, we also propose that receptor binding of Vpb4Aa2 may be involved in pore formation. Based on the fold of domain 5, this receptor could very well be proteinaceous (based on similarity to domain 5 of CdtB)^62^ or glycan (based on similarity to CBM-34-like proteins FIP-fve and FIP-nha)^38^. Thus, our proposed model of the Vpb4 activation involves proteolytic activation and receptor binding (Fig. 8). Whether pre-pore formation occurs during the pore formation pathway remains an open question.

Structure-guided clustering performed in our study delineates a Vpb4-like lineage that diverges both in sequence and pore architecture from the translocating Bacterial_Exotoxin_B proteins. Using heuristic FoldSeek^63^ query searches of Vpb4Aa2 PFDs in the AFDB, we were able to define a Vpb4-like clade containing all currently recorded Vpb4 subclass members and Vpb4-like candidates. All members lack the canonical phenylalanine ɸ-clamp, the structural signature of translocating family members, and instead carry smaller side-chain substitutions. This loss of the ɸ-clamp also correlates with a neutrally charged lumen in AlphaFold pore models (with the exception of Vpb4Ba1 and the A0A855DIW0 identified above). We also identified three family members that carry non-canonical ɸ-clamp and predicted negatively charged lumens. This includes the partially characterised BECb^47,48^, which has a charged lumen resembling canonical translocases, but with a wider ɸ-clamp reminiscent of Vpb4 clade.

Phylogenetically, while both the Vpb4 and Vpb1 toxins originated from *B. thuringiensis*, the branches of the two subclasses form a well-supported basal split, where Vpb1 clusters more closely with non-*Bacillus* translocating toxins. Because our tree is constrained by a limited number of curated sequences, we are unable to resolve the nature of the last common Bacterial_Exotoxin_B ancestor (independent cytolytic versus binary translocating pores). Nevertheless, the pore hallmarks identified offer a practical filter for mining AFDB and related resources for uncharacterised single-component cytolytic members of the family. Here, we propose that the two identified Vpb4-like proteins originated from *B. thuringiensis*, A0A193D1L2 and A0A2C9ZD13, are members of the Vpb4 subclass and may exhibit activity against invertebrates in a similar manner to Vpb4Fa1 and Vpb4Da2.

Collectively, our study explains the ability of members of the Vpb4 subclass to cause membrane damage directly through pore formation and provides explanations at the molecular level for this ability, in contrast to the translocase activity of related proteins. The structure and the biophysical characterisation of the Vpb4 subclass also lays the foundation for identification of previously uncharacterised cytolytic pores from the Bacterial_Exotoxin_B family. While Vpb4Aa2 targets and its mechanism of interaction with receptors remain to be elucidated, our findings provide a framework on deep database searches for identification and screening strategies for potential pesticidal proteins within the agricultural industry.

## Methods

### Cloning and production of Vpb4Aa2 protein

The *vpb4Aa2* gene sequence (accession MZ766551) was amplified from *Bacillus thuringiensis* strain V004.17. To generate the coding sequence (CDS) map, the contig sequence containing the *vpb4Aa2* gene was annotated using Prodigal^64^ v2.6.3 to predict CDS of putative protein, followed by protein prediction via NCBI blastx^65^ web interface (https://blast.ncbi.nlm.nih.gov/Blast.cgi), and IGV-Web^66^ (https://igv.org/app) for gene visualisation. The putative N-terminal signal peptide in the sequence (residues 1-28) was removed before being cloned into a pET22b-derived vector. The missing signal peptide was replaced by a periplasmic signal peptide derived from cytochrome b562, a His-tag to facilitate purification, and a TEV cleavage site (Supplementary Fig. 1).

The BL21 *Escherichia coli* expression system was used to produce the Vpb4Aa2 protein. The protein production was induced by the addition of 0.1 mM IPTG (final concentration) to a 500 mL *E. coli* culture (reaching OD_600_ value of 0.8–1). The culture was incubated at 18 °C for 16 h with shaking at 180 rpm. For periplasmic extraction of the protein, cell pellets were resuspended in 10 mL of TSE buffer (200 mM Tris-HCl pH 8, 500 mM sucrose, 1 mM EDTA) per gram of cells before incubating on ice for 30 min. The cell suspension was centrifuged at 8000 *g* for 20 min at 4 °C, and the pellets were further resuspended with an equal volume of MgSO_4_ solution (5 mM MgSO_4_, 1 mM EDTA, 1 mM PMSF) before incubating on ice for 15 min. The suspension was centrifuged again at 8000 g for 20 min, and the periplasmic fraction in the supernatant was collected for purification of Vpb4 protein.

The periplasmic content was initially batch-bound with 3 mL of Ni-NTA resin (Qiagen) on a roller for 1 h at 4 °C. Following batch binding, the resin was poured into a 2.5 × 10 cm Econo-Column® Chromatography column (Bio-Rad) and the flowthrough was collected. The resin was then washed with 5 × 5 mL wash buffer (30 mM Tris-HCl pH 8, 10 mM imidazole). Nickel-NTA bound proteins were eluted with 8 ×3 mL elution buffer (30 mM Tris-HCl pH 8, 200 mM imidazole). The presence of Vpb4Aa2 protein in the elution was confirmed by SDS-PAGE analysis and Bradford assay. Once confirmed, the eluates were dialysed in a dialysis buffer (20 mM Tris-HCl pH 8, 150 mM NaCl) overnight ( ~ 16 h) at 4 °C.

Following dialysis, the Vpb4Aa2 was further purified through size exclusion chromatography (SEC) using a Superdex 200 16/600 column (Cytiva) in SEC buffer (20 mM Tris-HCl pH 8, 150 mM NaCl). SEC was performed in Unicorn v5.31 (Cytiva) system. The presence of Vpb4Aa2 protein in the UV chromatogram peak was confirmed by SDS-PAGE. Purified Vpb4Aa2 protein was concentrated to 2 mg/mL and stored at 4 °C for no more than 1 week before trypsinisation. Protein concentration was determined using Bradford assay (Bio-Rad).

### Conversion to Vpb4Aa2-pore in detergent

Removal of the pro-domain and oligomerisation into Vpb4Aa2 pores was achieved by trypsin digestion of the Vpb4Aa2 at 1 mg/mL in the presence of detergents Lauryl Maltose Neopentyl Glycol (LMNG, 0.01% w/v) and Cholesteryl Hemisuccinate (CHS, 0.001% w/v). Trypsinisation was performed on Vpb4Aa2 at a 1:50 molar ratio of trypsin to Vpb4Aa2, overnight ( ~ 16 h) at room temperature. Following trypsinisation, the Vpb4Aa2 pores were subjected to SEC using a Superdex 200 10/300 column (Cytiva) in 20 mM Tris-HCl pH 8, 150 mM NaCl, 0.001% w/v LMNG, 0.0001% w/v CHS. The trypsin-activated Vpb4Aa2 was subsequently concentrated to 7.5 mg/mL for plunge freezing on cryo-EM grids for data collection.

### Ethanol incubation and mass photometry

Following the protocol for Iota-B oligomerisation^21^, we treated Vpb4Aa2 with 10% v/v EtOH during the trypsinisation process (conditions as mentioned above). The protein was then subjected to SEC using a Superose 6 10/300 column (Cytiva) in 20 mM Tris-HCl pH 8, 150 mM NaCl, 0.001% w/v LMNG, 0.0001% w/v CHS. Fractions were collected for SDS-PAGE and mass photometry to observe oligomer formation. Mass photometry experiments were conducted on TwoMP mass photometer (Refeyn). Data collection was performed on the AcquireMP v2024R1 (Refeyn) software. Mass calibration was performed on DiscoverMP v2024R1 (Refeyn) software using three standards: BSA (66 kDa), Apoferritin (480 kDa), and Thyroglobulin (660 kDa). Three fractions were used for the experiment to highlight the presence of monomers, oligomers, and the detergent peak. The histograms were graphed on GraphPad Prism v10.4.1 at a bin width of 5.

### Sample preparation, data collection and data processing

All cryo-EM experiments were performed at the Monash Ramaciotti Cryo-EM Facility. Before vitrification, a copper R1.2/1.3 Quantifoil Holey Carbon Grid (Quantifoil Micro Tools GmbHa) was glow discharged for 30 s in ambient atmospheric conditions at 38 mbar. Sample vitrification was performed using Mark IV Vitrobot (FEI), where 3 μL of protein sample were blotted on the glow-discharged grid. Proteins were screened on Tecnai G2 T20 Electron Twin TEM (FEI). Data collection was performed using the EPU Software v2.12.1.2782 (Thermo Fisher) on Titan Krios Cryo-TEM (FEI) with a Quantum energy filter (Gatan) and K3 Bioquantum detector (Gatan) at a pixel size of 0.82 A^2^ and 60 e/A^2^ total dose.

Single particle cryo-EM analysis was performed using RELION-5.0 through the M3 MASSIVE High-Performance Computing facility (Supplementary Fig. 13). The raw movies were subjected to motion correction with MotionCor3 and Contrast Transfer Function (CTF) estimation with CTFFIND4^67^. Initial particle picking was performed using Laplacian of Gaussian picking at a maximum and minimum diameter of 325 Å and 175 Å on 347 micrographs. Particles were then extracted with a box size of 400 pixels and binned five-times prior to 2D classification to 50 2D classes. Classes with observable pore features were used for Topaz training and picking^68^, followed by iterative 2D classifications. The final 2D classes were selected for 3D initial reference and 3D classifications to five 3D classes. The final set of particles were re-extracted at 320 pixels prior to 3D auto-refine job with applied C1 or C7 symmetry. Particles then underwent CTF refinement^69^ and Bayesian polishing^70^ prior to the final round of 3D auto-refine. All refinement jobs were run using blush regularisation^71^. Masked refinement of the poorly resolved density was trialled, but no significant improvement was observed. The raw map is deposited to EMDB under the code EMD-71647 for the C7 reconstruction and code EMD-68793 for the C1 reconstruction.

### Model generation of the Vpb4Aa2 pore

Due to the limited C-terminal densities, two different models of Vpb4Aa2 were generated in this study: the pore model and the full-length subunit model. The pore model of Vpb4Aa2 was generated into the C7 symmetry map, encompassing domains 1-5 (N216-N853, ~81% complete chain). The β-barrel pore was modelled using the pre-existing cryo-EM model of CdtB (PDB: 7YVS, 35% sequence identity), while the remaining portion of the model was generated using ColabFold^72^. Due to limited densities, a loop in D5 (N828-D831) was omitted, while residues N346 and K347 at the tip of the β-barrel were replaced with alanine.

The full-length subunit model of Vpb4Aa2 encompassed domains 1-6 (N216-S965, 94% complete chain), incorporating domains 1-5 from the C7 model and the auxiliary C-terminal domain 6. The C-terminal domain 6 was generated using ColabFold^72^. Due to poor resolution, domain 6 was docked (rather than built) into the density using the fitmap command in ChimeraX 1.10.1^73^, as highlighted in Supplementary Fig. 9. Domain 6 was modelled as backbones with missing residues at S875-Y883, D921-R925, and G935-R937. The domain movement between pro-Vpb4 (pro-Vpb4Da2 as representative structure) and full-length subunit of Vpb4Aa2 pore was visualised using the morph command in ChimeraX 1.10.1^73^, as shown in Supplementary Movie 1.

For model building and confirmation of calcium ions in domain 4, the two reconstructed (C7 and C1) volumes were initially sharpened using EMReady V2.0^74,75^. Model building and localised real-space-refinement were performed on WinCoot 0.9.8^76^ and Isolde 1.6.0^77^. Global real-space-refinement and validation were performed on Phenix 1.20^78^ before deposition in PDB. The assigned codes are 9PHF for the Vpb4Aa2 pore and 22ZO for the full-length subunit. Validations of both models are summarised in Supplementary Table 1. Local density during model-building is highlighted in Supplementary Fig. 9. One-to-one sequence identity and similarity was calculated on EMBOSS Needle as part of EMBL-EBI Job Dispatcher^79^. Multiple sequence alignment and group sequence identity calculation were performed on Clustal Omega as part of EMBL-EBI Job Dispatcher^79^. Analysis of the pore lumen diameter was generated in Hole2^80^ v2.3.1 and graphed in GraphPad Prism v10.4.1.

### Electrophysiology methods

In a Faraday Cage, a PMMA incubation chip was taped to a reflective surface on a stage. A trough in the chip was filled with 4 mg/mL 1,2-diphytanoyl-sn-glycero-3-phosphocholine (DPhPC, Avanti Polar Lipids) dissolved in 60% v/v hexadecane (Sigma-Aldrich) and 40% v/v silicone oil (Sigma-Aldrich). Then, 0.1 mm Ag/AgCl electrodes dipped into molten 0.75% w/v low gelling temperature agarose (Sigma-Aldrich) were mounted onto micromanipulators. Electrodes were plugged into a headstage (203BU) of a patch-clamp amplifier (Axopatch 200B, Axon Instruments, Molecular Devices, California, USA), then lowered into the lipid-oil mixture. Droplets of 0.2 µL were pipetted onto each electrode. The droplet on the working electrode contained 40 μg/mL Vpb4Aa2 in 1 M KCl, 10 mM HEPES, pH 7, or, where specified, 0.5 M CaCl_2_, 10 mM HEPES, pH 7. The droplet on the ground electrode was 1 M KCl, 10 mM HEPES, pH 7, or, where specified, 0.5 M CaCl_2_, 10 mM HEPES, pH 7.

After 10 min of incubation time, droplets were brought together until touching, at which point a ± 23 mV triangular wave at 10 Hz was applied. The current was passed through a digital analogue converter (National Instruments BNC-2110) to a PC and was recorded using WinEDR v4.0.4 (University of Strathclyde). Upon bilayer formation, the triangular wave was stopped, and a voltage protocol, measuring in 10 mV increments, was applied for the range ±10 mV to ±70 mV. Post acquisition processing was performed in MatLab vR2023b (MathWorks), during which a Chung-Kennedy filter, a forward-backward non-linear filter, was applied^81^. For experiments investigating anthrax PA, the working electrode droplet was 40 *μ*g/mL PA in 1 M KCl, 10 mM MES pH 5.5 or 0.4 M CaCl_2_, 12 mM MES, pH 5.5 and the ground electrode droplet was the same buffer, without PA.

### TIRF microscopy methods

A coverslip was plasma-treated (Femto Plasma cleaner, Diener Electronic, Ebhausen, Germany), and 120 μL molten 0.75% w/v low gelling temperature agarose (Sigma-Aldrich) in water was spin coated (WS-650MZ-23NPP/LITE, Laurell, Pennsylvania, US) onto the coverslip. The coverslip was mounted onto a 16-well PMMA chip and sealed by pipetting 170 *μ*L molten 3.5% w/v low gelling temperature agarose (Sigma-Aldrich) in 0.33 M CaCl_2_, 10 mM HEPES into a microfluidic channel. The PMMA chip was taped inside a Faraday Cage, on the stage of an inverted microscope (Nikon Ti-U Eclipse, Nikon Instruments), mounted on an optical table (Nexus, ThorLabs). A 1 mm Ag/AgCl electrode was inserted into the microfluidic channel and plugged into the ground port of the headstage of a patch-clamp amplifier. DPhPC (8 mg/mL) dissolved in 60% v/v hexadecane and 40% v/v silicone oil was pipetted into each well of the chip, and into the trough of a PMMA incubation chamber.

A droplet solution, composed of 1 M KCl, 6.67 mM HEPES, pH 7.0, 400 *μ*M EDTA (Sigma-Aldrich), 50 *μ*M Fluo-8 (AAT BioQuest), and 470 nM activated Vpb4Aa2 was made. Droplets <0.05 *μ*L were pipetted into a trough and allowed to incubate for 30 minutes. An incubated droplet was transferred to a well in the PMMA chip and monitored visually for bilayer formation. The droplet was then attached to a 0.1 mm Ag/AgCl electrode dipped into molten 0.75% w/v low gelling temperature agarose mounted onto a micromanipulator and plugged into the working port of the headstage of a patch-clamp amplifier.

Bilayers were investigated using a 60x magnification TIRF lens (CFI Apochromat TIRF 60XC Oil, Nikon Instruments), with Fluo-8 illuminated by a 473 nm laser (Ventus, Laser Quantum UK). The optics are depicted in Supplementary Fig. 14. The resulting photons were captured by a charge-coupled device (Andor iXon Ultra EMCCD, Oxford Instruments, UK), which was controlled through the Andor Solis I Software v4.29.300012.0 (Oxford Instruments, UK). Post acquisition processing was performed in the Fiji^82^ distribution of ImageJ v1.54p and MatLab vR2023b (MathWorks).

### Cell culture methods and toxicity assays

The activity of purified and activated Vpb4Aa2 was tested on three insect cell lines: two isolated from *Spodoptera frugiperda*, Sf9 (11496015, Thermo Fisher) and Sf21 (11497013, Thermo Fisher), and one from *Diabrotica virgifera virgifera*, DvWL2 (USDA, ARS Biological Control of Insects Research Laboratory). Cell lines from *S. frugiperda* were maintained in Grace’s Insect Medium (G9771, Sigma-Aldrich) supplemented with 10% v/v foetal bovine serum (FBS, Pan Biotech), DvWL2 cells were maintained in EX-CELL 420 Medium (14420 C, SAFC) supplemented with 10% v/v heat-inactivated FBS and 1% w/v penicillin-streptomycin (company). All cell lines were grown at 28 °C with ambient CO_2_. For bioassays, cells were plated in 96-well plates at 10,000 cells/well in 150 µL of culture medium. Once cells had reached 70% confluency, Vpb4Aa2 was added at a range of concentrations (10, 50 and 100 µg/mL). Buffer only was added as a negative control. For activation, Vpb4Aa2 was trypsin activated using immobilised TPCK treated trypsin resin (20233, Thermo Scientific, Waltham, MA, USA) at 37 °C for 16 h, followed by centrifugation to remove the resin prior to use. At 48 h post Vpb4Aa2 addition, cellular viability was assessed via resazurin assay as described previously^83^. Briefly, the culture medium of the cells was replaced with culture medium containing 10 μg/mL resazurin diluted in DPBS (10% v/v), and the cells were incubated at 37 °C for 4 h. Fluorescence was measured using a Spectramax Gemini EM plate reader (Molecular Devices) at excitation and emission wavelength of 445 nm and 585 nm, respectively.

### Phylogenetic analysis for characterised family members

Initial phylogenetic analysis was performed on all Vpb4 subclass members from BPPRC^1,84^ (Vpb4Aa1, Vpb4Aa2, Vpb4Ba1, Vpb4Ca1, Vpb4Da2, and Vpb4Fa1), and other characterised Bacterial_Exotoxin_B members (PA, CdtB, Iota-B, C2-II, and Vpb1Ac1). Accession codes are listed in Supplementary Table 5. For phylogenetic analysis, multiple sequence alignments were performed on MAFFT^85^ v7.526 using local pairwise alignment strategy and 1000 iterative refinement cycles. Gaps were then trimmed using trimAL^86^ v1.5 in automated mode. The phylogenetic tree was constructed in IQ-TREE^87^ v3.0 based on a model generated from ModelFinder^88^ and supported with 1000 ultrafast bootstrap approximations^89^. The phylogenetic tree was visualised using iTOL^90^ v7, and rooted at the midpoint.

### Database search for uncharacterised Bacterial_Exotoxin_B members

The database search for phylogenetic analysis was performed using FoldSeek^63^ of domain 1-3 of Vpb4Aa2 against PDB^91^ and AFDB^92,93^ databases (last accessed 19 April 2025). Candidates for analysis consisted of AFDB hits with Prob. > 0.8, TM-score > 0.6, and average pLDDT > 0.7 and each of the Vpb4 subclass members (Vpb4Aa1, Vpb4Aa2, Vpb4Ba1, Vpb4Ca1, Vpb4Da2, and Vpb4Fa1) and characterised Bacterial_Exotoxin_B members (PA, CdtB, Iota-B, C2-II, and Vpb1Ac1). All sequence accessions and model metadata are provided in Supplementary Tables 5 and 6.

Phylogenetic analysis was performed as described for characterised family members, except trimming was performed using a gap threshold of 10% instead to prevent over-trimming. Clades are annotated based on the genus of origin. Based on the sequence alignment, nodes with non-canonical ɸ-clamps were labelled and selected for pore lumen prediction. To assign the lumen charge, D1-3 of each node was selected based on alignments against Vpb4Aa2 and queried as 7 copies into the AlphaFold Server^94^.

### Reporting summary

Further information on research design is available in the Nature Portfolio Reporting Summary linked to this article.

## Supplementary information

Supplementary Information

Description of Additional Supplementary Files

Supplementary Movie 1

Supplementary Movie 2

Reporting Summary

Transparent Peer Review file

## Source data

Source Data

## Data Availability

The Vpb4Aa2 pore complex in C7 symmetry model and map have been deposited in the PDB database under accession code 9PHF 10.2210/pdb9phf/pdb (pdb_00009PHF) and in the EMDB database under accession code EMD-71647. The single full-length subunit of the Vpb4Aa2 pore complex model has been deposited in the PDB database under accession code 22ZO 10.2210/pdb22zo/pdb (pdb_000022ZO). The C1 reconstruction of the pore has been deposited in the EMDB database under accession code EMD-68793 in EMDB. All models from other publications used for structural comparison in this study are summarised in Supplementary Table 5 are available in the PDB database under accession codes 7KXR 10.2210/pdb7kxr/pdb, 6UZB 10.2210/pdb6uzb/pdb, 6KLW 10.2210/pdb6klw/pdb, 6O2N 10.2210/pdb6o2n/pdb, 7VNN 10.2210/pdb7vnn/pdb, 6SMS 10.2210/pdb6sms/pdb, 7MJR 10.2210/pdb7mjr/pdb, 9FM6 10.2210/pdb9fm6/pdb, 9KG6 10.2210/pdb9kg6/pdb, 1OSY 10.2210/pdb1osy/pdb, and 8GO5 10.2210/pdb8go5/pdb. Protein accession codes are available in the Uniprot database for P13423, Q46221, A8DS70, O86171, V9I0N3, A0A2U5GYP0, and A0A2U5FTM5; and NCBI GenBank for AEH05932, MZ766551, OUB77819, and PQ478524. All chromatography data, uncropped gels, Hole2 measurement data, and cell-based assay data and minimum data for electrophysiology graphs (single I/V relationship and conductance comparisons) are provided in the Source Data file. All raw data for electrophysiology experiments were deposited in the Zenodo repository 10.5281/zenodo.19552438. Source data are provided with this paper.
