# Supplementary material for: Structural basis for independent pore function of Vpb4 from Bacillus thuringiensis
Source: Nat Commun. 2026 Jul 1;17:8198. doi: 10.1038/s41467-026-74567-y (PMC13462110; doi:10.1038/s41467-026-74567-y)
Supplement: Supplementary file 1 [file 41467_2026_74567_MOESM1_ESM.pdf]

Structural basis for independent pore function of Vpb4 from *Bacillus thuringiensis*

Raymond Wirawan[1,#], David Jamieson[2,#], Hannah Baird[2,#], Colin Berry[3], Chris J. Lupton[1], Hari Venugopal[5], Luis E. Valentin-Alvarado[1], Hannah L. Best[3], D. Dafydd Jones[3], Lainey J. Williamson[3], Husam Sabah Auhim[3,4], Oliver Castell[2,*,%], Michelle Dunstone[1,*], Bradley Spicer[1,*,%]

Biomedicine Discovery Institute, Monash University, Clayton, Victoria, Australia

School of Pharmacy and Pharmaceutical Sciences, Cardiff University, Cardiff, UK

School of Biosciences, Cardiff University, Cardiff, UK

Department of Biology, College of Science, University of Baghdad, Al-Jadriya Campus, Baghdad, Iraq

Ramaciotti Centre for Cryo-Electron Microscopy, Monash University, Clayton, Victoria, Australia

# These authors contributed equally.

*These authors jointly supervised the work.

% Correspondence: Bradley.Spicer@monash.edu, CastellO@cardiff.ac.uk

Supplementary Information

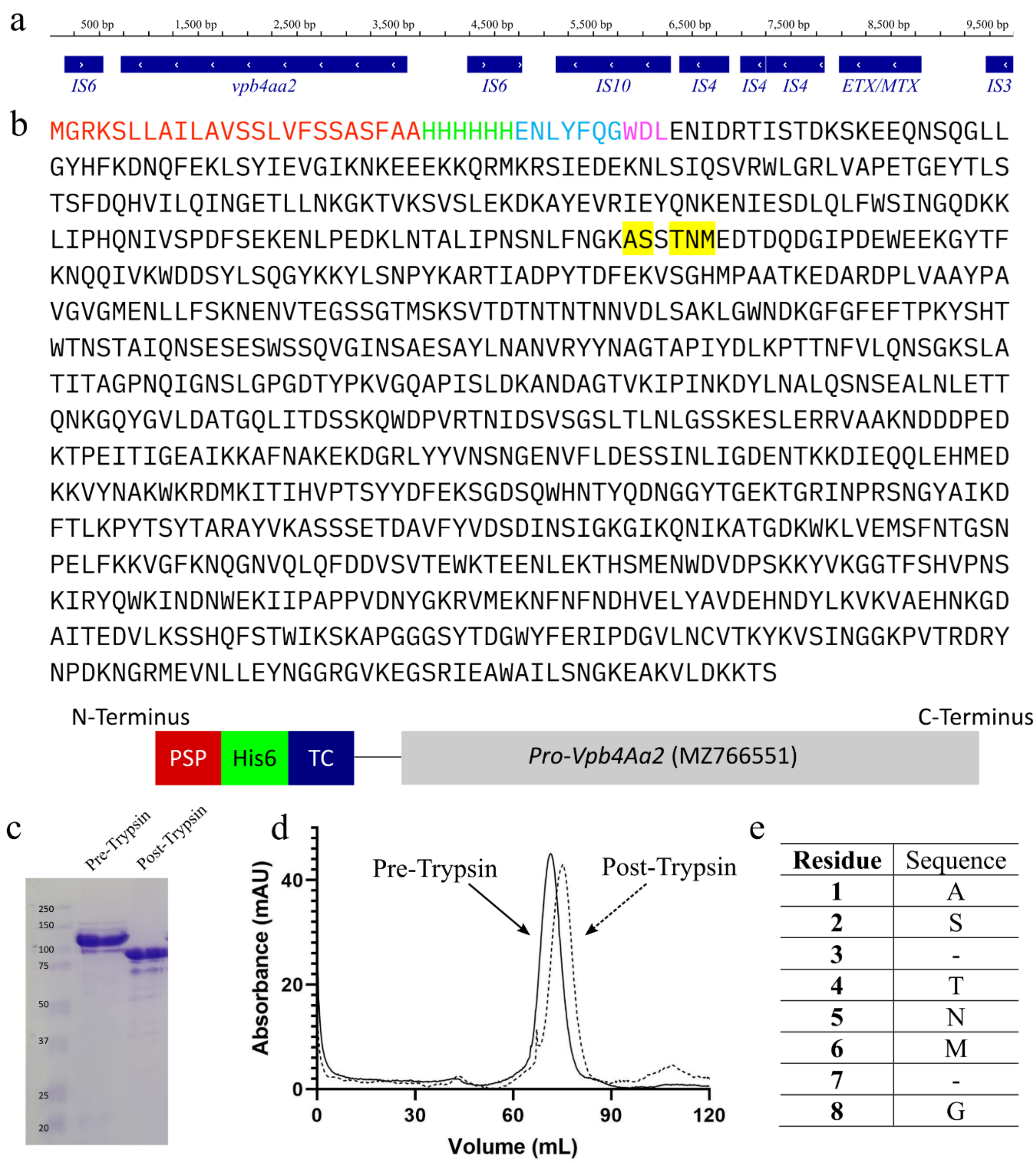

Residue	Sequence
1	A
2	S
3	-
4	T
5	N
6	M
7	-
8	G

Supplementary Figure 1 Vpb4Aa2 construct and trypsin activation. a) Annotated CDS map of Vpb4Aa2 in the contig of *B. thuringiensis* strain V004.17 highlights lack of A components. **b)** The Vpb4Aa2 construct used in this study and schematics, with periplasmic signal peptide (PSP) in red (derived from Cyt b562), 6xHis-tag (His6) in green, and TEV cleavage site (TC) in blue. The text highlighted in yellow marks residues identified by N-terminal sequencing. **c)** SDS-PAGE bands of Vpb4Aa2 before and after trypsinisation. **d)** Size exclusion chromatography profile of Pro-Vpb4Aa2 before and after trypsinisation on Superdex 200 16/600 column. **e)** N-terminal sequence analysis of Vpb4Aa2 after trypsinisation.

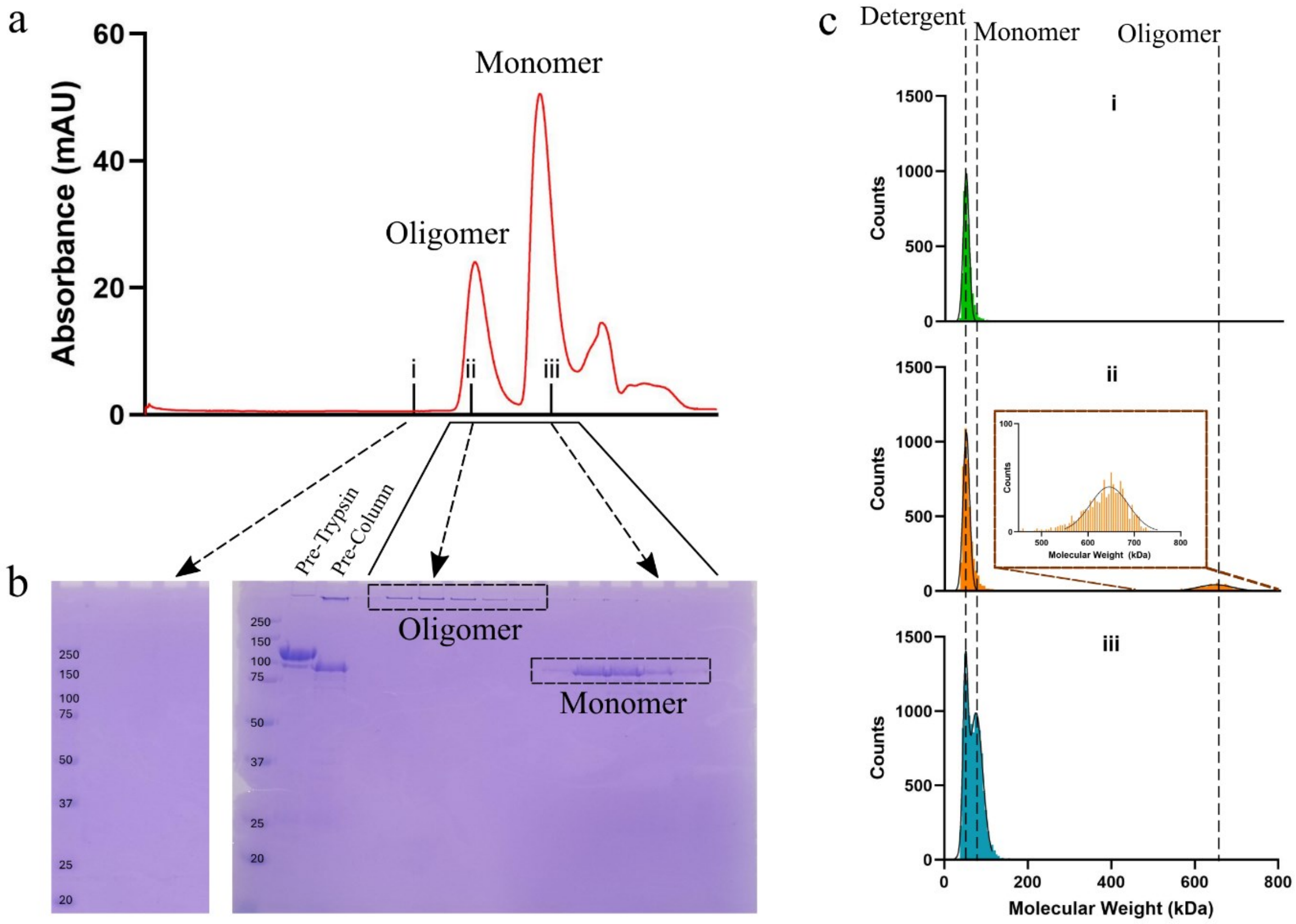

Supplementary Figure 2 Characterisation of Vpb4Aa2 oligomerisation in the presence of EtOH and Detergent. a) Size exclusion chromatography profile of Vpb4Aa2 with EtOH incubation resolved on a Superose 6 10/300 column. Fractions analysed by mass photometry experiments are highlighted. b) Fractions of EtOH-incubated Vpb4Aa2 on SDS-PAGE (non-reducing and non-boiled) with molecular weight markers labelled. c) Mass photometry histogram (bin width = 5) of fraction (i) representing background detergent when no protein is present, fraction (ii) representing oligomer peak with an inset ranging from 450 – 800 kDa, and fraction (iii) representing monomer peak.

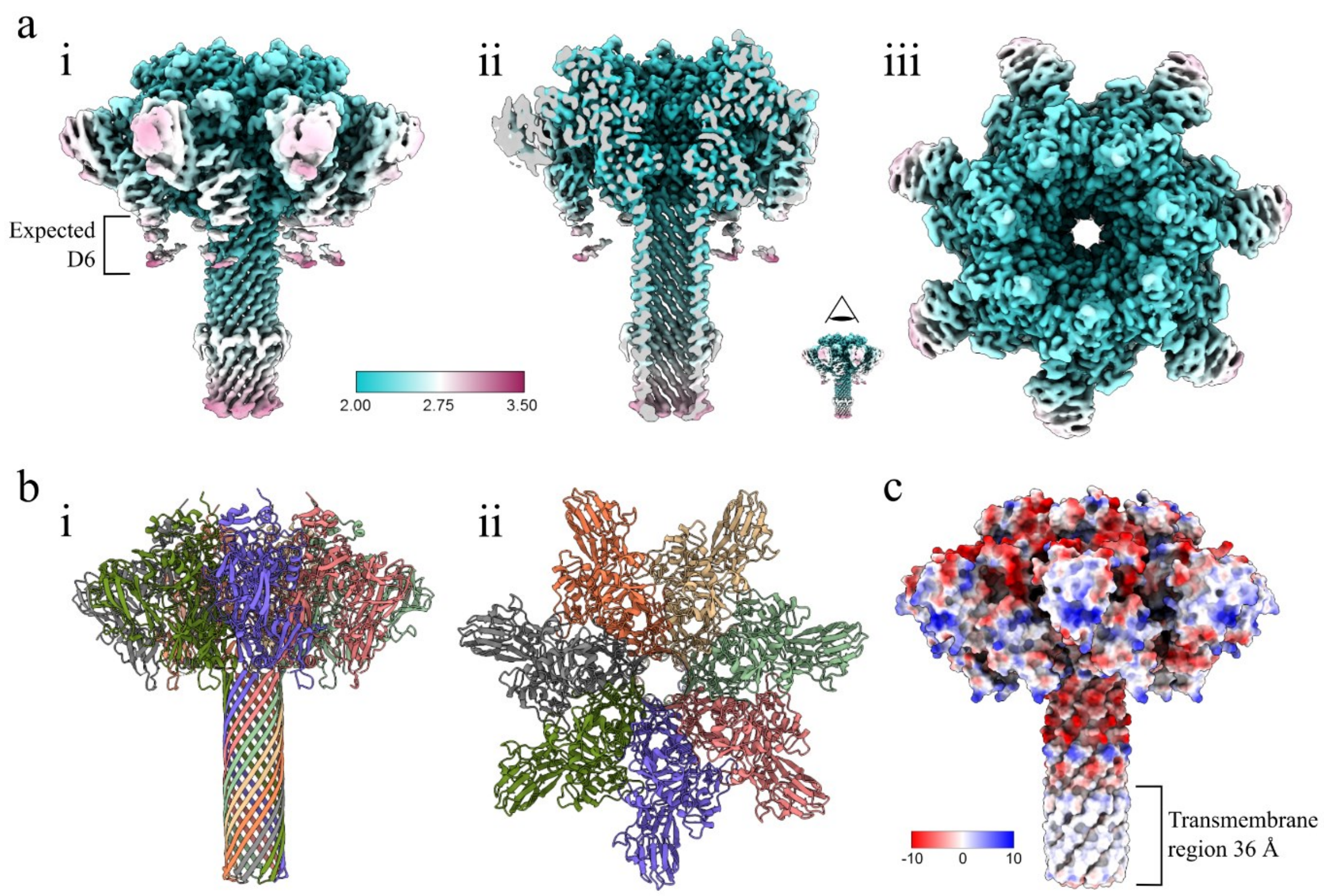

Supplementary Figure 3 Local resolution, ribbon model, and electrostatic potential of Vpb4Aa2 Pore. a) Local resolution map of Vpb4Aa2 pore in C7 symmetry: (i) front view; (ii) cross-section view; and (iii) top view with associated colour key for resoltuion. b) Ribbon representation of Vpb4Aa2 pore model: (i) front view; ii) top view. c) Electrostatic potential surface representation of Vpb4Aa2 pore with its associated colour key at front view, highlighting the transmembrane region.

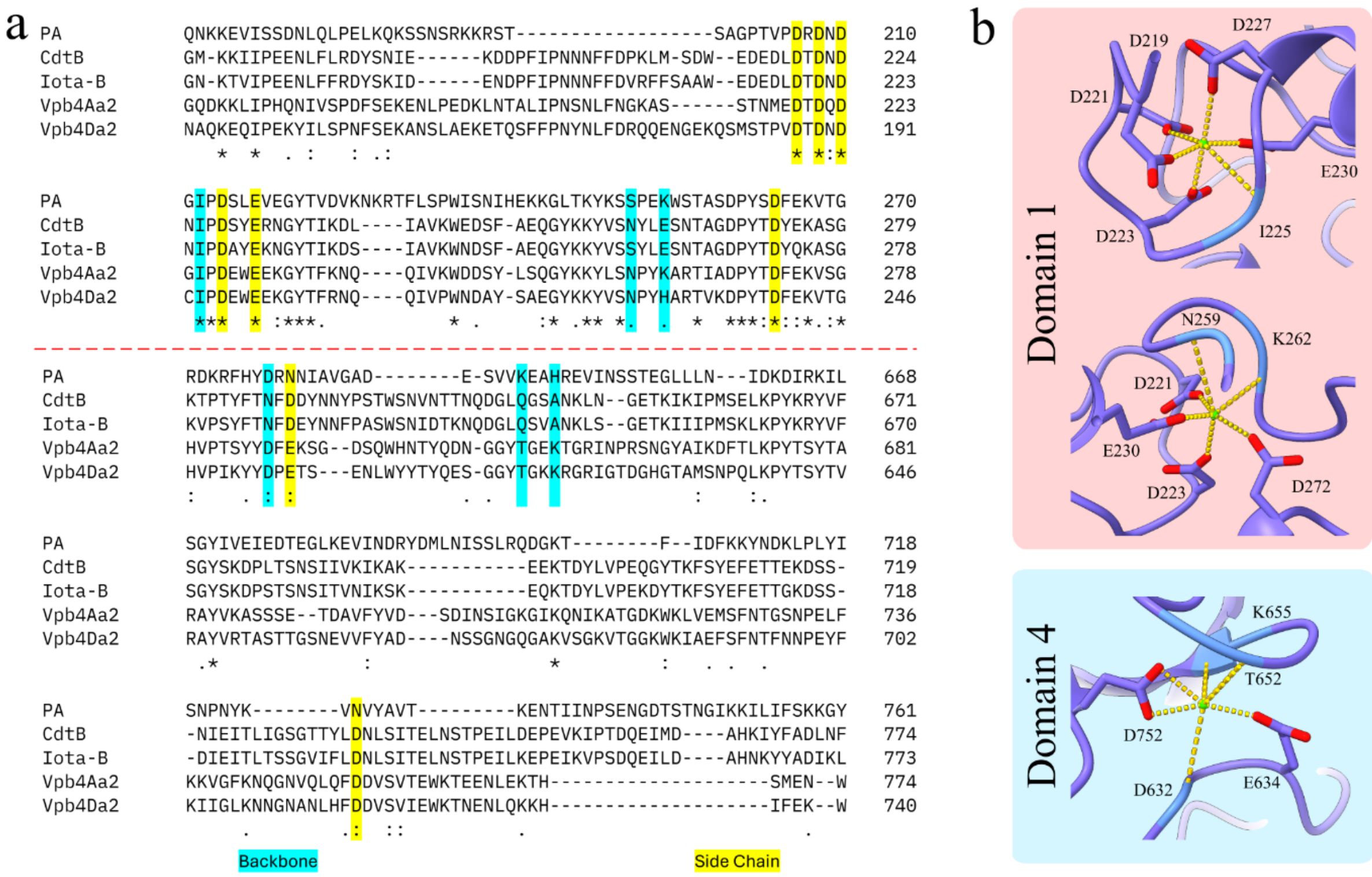

Supplementary Figure 4 Calcium-binding residues and domain 4 comparison. a) Sequence alignment of the calcium-binding residues (side-chain interactions in yellow and backbone in blue) in the Bacterial_Exotoxin_B family. **b)** Calcium-binding residues in Domain 1 (shaded red) and Domain 4 of Vpb4Aa2 (shaded blue).

```
PA              QNKKEVISSDNLQLPELKQKSSNSRKKRST------------------SAGPTVPDRDND    181
CdtB            GM-KKIIPEENLFLRDYSNIE------KDDPFIPNNNFFDPKLM-SDW--EDEDLDTDND    224
Iota-B          GN-KTVIPEENLFFRDYSKID------ENDPFIPNNNFFDVRFFSAAW--EDEDLDTDND    223
Vpb4Aa2         GQDKKLIPHQNIVSPDFSEKENLPEDKLNTALIPNSNLFNGKAS------STNMEDTDQD    223
Vpb4Da2         NAQKEQIPEKYILSPNFSEKANSLAEKETQSFFPNYNLFDRQQENGEKQSMSTPVDTDND    191
                   *  *  . :   : .:                                    * *:*

PA              GIPDSLEVEGYTVDVKNKRTFLSPWISNIHEKKGLTKYKSSPEKWSTASDPYSDFEKVTG    241
CdtB            NIPDSYERNGYTIKDL----IAVKWEDSF-AEQGYKKYVSNYLESNTAGDPYTDYEKASG    279
Iota-B          NIPDAYEKNGYTIKDS----IAVKWNDSF-AEQGYKKYVSSYLESNTAGDPYTDYQKASG    278
Vpb4Aa2         GIPDEWEEKGYTFKNQ----QIVKWDDSY-LSQGYKKYLSNPYKARTIADPYTDFEKVSG    278
Vpb4Da2         CIPDEWEEKGYTFRNQ----QIVPWNDAY-SAEGYKKYVSNPYHARTVKDPYTDFEKVTG    246
                 ***  * :***.           * .     :* .** *.  .  *  ***:*::*.:*

PA              LNAQDDFSSTPITMNYNQFLELEKTKQ-LRLDTDQVYGNIATYNFENGRVRVDTGSNWSE    476
CdtB            LNTMDQFSSRLIPINYDQLKKLDAGKQ-IKLETTQVSGNFGTKN-SSGQIVTEG-NSWSD    505
Iota-B          LNTMDQFNARLIPINYDQLKKLDSGKQ-IKLETTQVSGNYGTKN-SQGQIITEG-NSWSN    504
Vpb4Aa2         LDKANDAGTVKIPINKDYLNALQSNSEALNLETTQNKGQYGVLD-ATGQLITDSSKQWDP    509
Vpb4Da2         LDKANEAGTVKIAINAEQLDKIQAGTEILNIETTQNRGQYGILD-EKGQVIPG--GEWDP    475
                *:  :: .:  * :* : :  ::  .: :.::* *  *: .  :   *::      .*.
```

α-Clamp   NSS/NSQ Loop

Supplementary Figure 5 Secondary Toxin Binding Residues in PA, CdtB, and Iota-B. For α-clamp, the highlighted residues are R178, L187, F202, L203, P205, I207, I210, F236, and F464 of PA. The red box represents the only α-clamp residue conserved in Vpb4Aa2. For the NSS/NSQ loop, the residues highlighted in green are N491-S493 for CdtB and N490-Q492 for Iota-B.

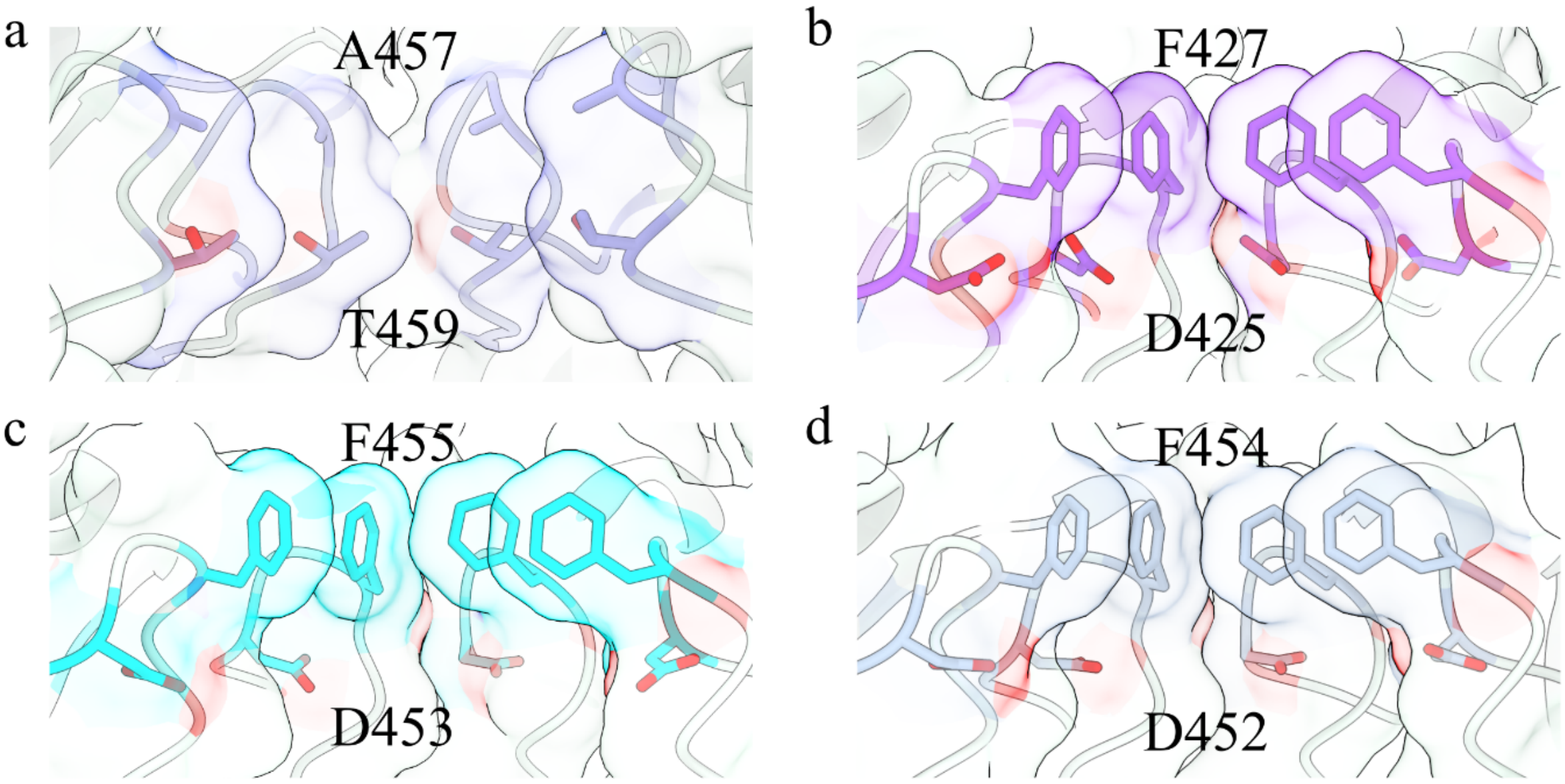

e

```
Vpb4Aa2 DKANDAGTVKIPINKDYLNALQSNSEALNLETTQNKGQYGVLDA-TGQLITDSSKQWDPV
Vpb4Aa1 DKANDAGTVKIPINKDYLDALQSNSEALDLETTQNKGQYGVLDA-TGQLITDSSKQWDPV
Vpb4Ba1 DRLEDG--TRISINAEQLDSIQNNSKKLSLETPQVGGQYGLLRD--GGLVINDFQTWAPI
Vpb4Ca1 DKANEAGTMKISLNAAQLDSIQSQGEIIDLETTQSSGQYGILDPSNGNLITDAGKQWDHI
Vpb4Da2 DKANEAGTVKIAINAEQLDKIQAGTEILNIETTQNRGQYGILDE-KGQVIPG--GEWDPI
Vpb4Fa1 DKANEAGTVKIAINADQLDALQDRSEVLNLETTQNKGQYATLDD-TGLPVTDPSKQWDPI
        *: ::.   :* :*   *: :*   : :.:** *  ***. *    *  :      *  :

Vpb4Aa2 DKANDAGTVKIPINKDYLNALQSNSEALNLETTQNKGQYGVLD-ATGQLITDSSKQWDPV
PA      NAQDDFSSTPITMNYNQFLELEKTKQ-LRLDTDQVYGNIATYNFENGRVRVDTGSNWSEV
CdtB    NTMDQFSSRLIPINYDQLKKLDAGKQ-IKLETTQVSGNFGTKN-SSGQIVTEG-NSWSDY
Iota-B  NTMDQFNARLIPINYDQLKKLDSGKQ-IKLETTQVSGNYGTKN-SQGQIITEG-NSWSNY
        :  :: .:  * :* : :  ::  .: :.::* *  *: .  :   *::      .*.

  PA_D425             ϕ-clamp             Vpb4Aa2_T459
 Equivalent                                Equivalent
```

Supplementary Figure 6 ϕ-clamp environment of Vpb4Aa2 pores and related toxins. Side view of the ϕ-clamp loop of **a)** Vpb4Aa2, **b)** PA, **c)** CdtB, **d)** Iota-B. **e)** Sequence alignment of residues around the ϕ-clamp among the Vpb4 subclass (top) and structurally characterised Bacterial_Exotoxin_B members (bottom).

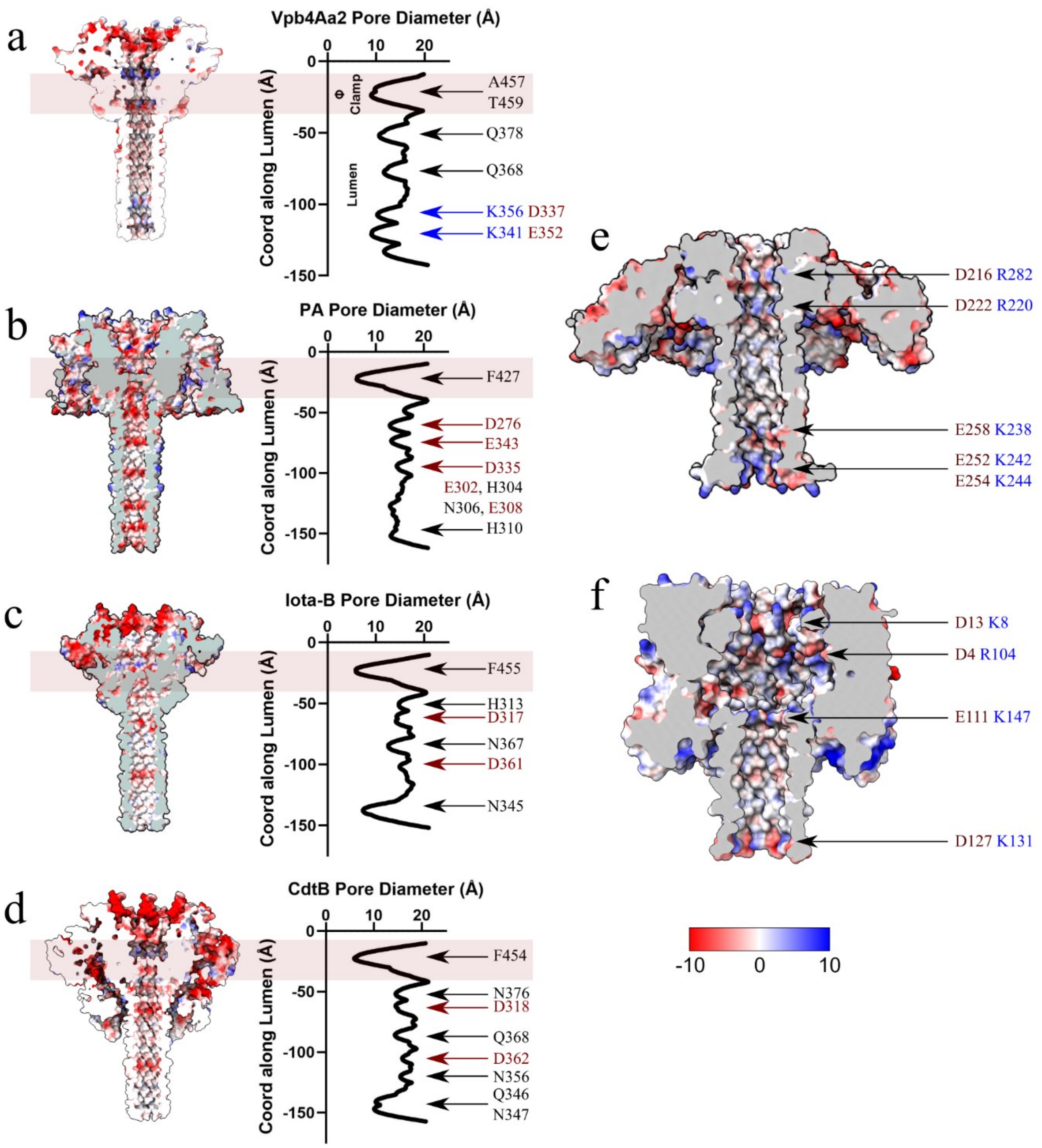

Supplementary Figure 7 Pore lumen profile of related toxins. Electrostatic potential surface representation and pore diameter profile and associated colour key of **a)** Vpb4Aa2, **b)** PA (7KXR), **c)** Iota-B (6KLW), and **d)** CdtB (6O2N). Electrostatic potential surface representation and complementary charge pairings of **e)** Aerolysin (9FM6) and **f)** Alpha-hemolysin (9KG6).

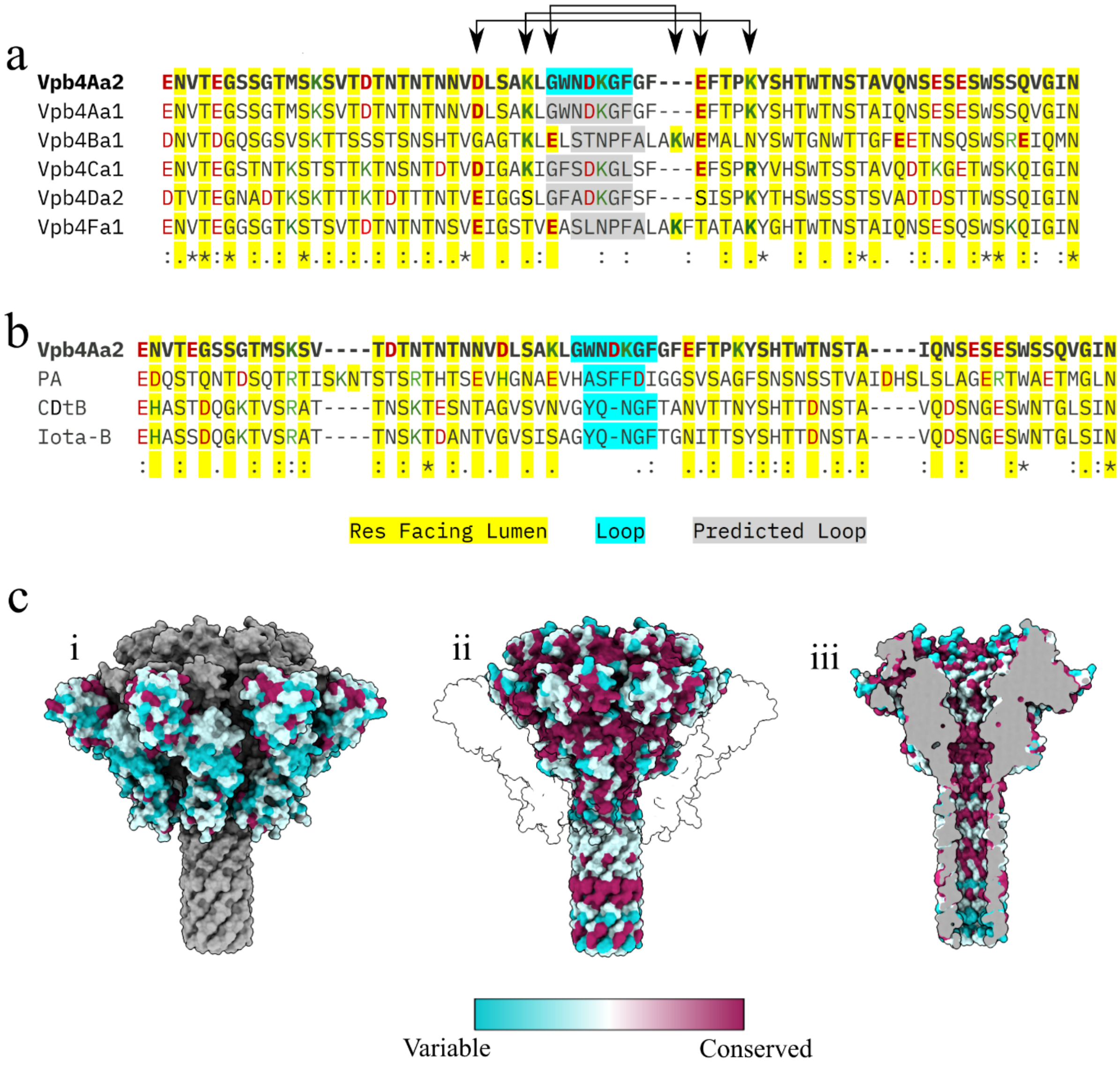

Supplementary Figure 8 Sequence alignments and conservation colouring of the pore-forming loop of Vpb4Aa2 and related toxins. Sequence alignments between **a)** Vpb4 members and **b)** related toxins. The loop at the end of the β-barrel is highlighted based on experimental structure (cyan) and AlphaFold prediction (grey). Yellow highlights lumen-interfacing residues, where arrows indicate complementary charged residues within the lumen. **c)** Conservation colouring of the Vpb4Aa2 pore compared to the sequence of Vpb4Da2 and Vpb4Fa1 with associated colour key at the (i) receptor binding domains (RBD, domains 4-6), (ii) pore-forming domains (PFD, domains 1-3), and (iii) pore lumen.

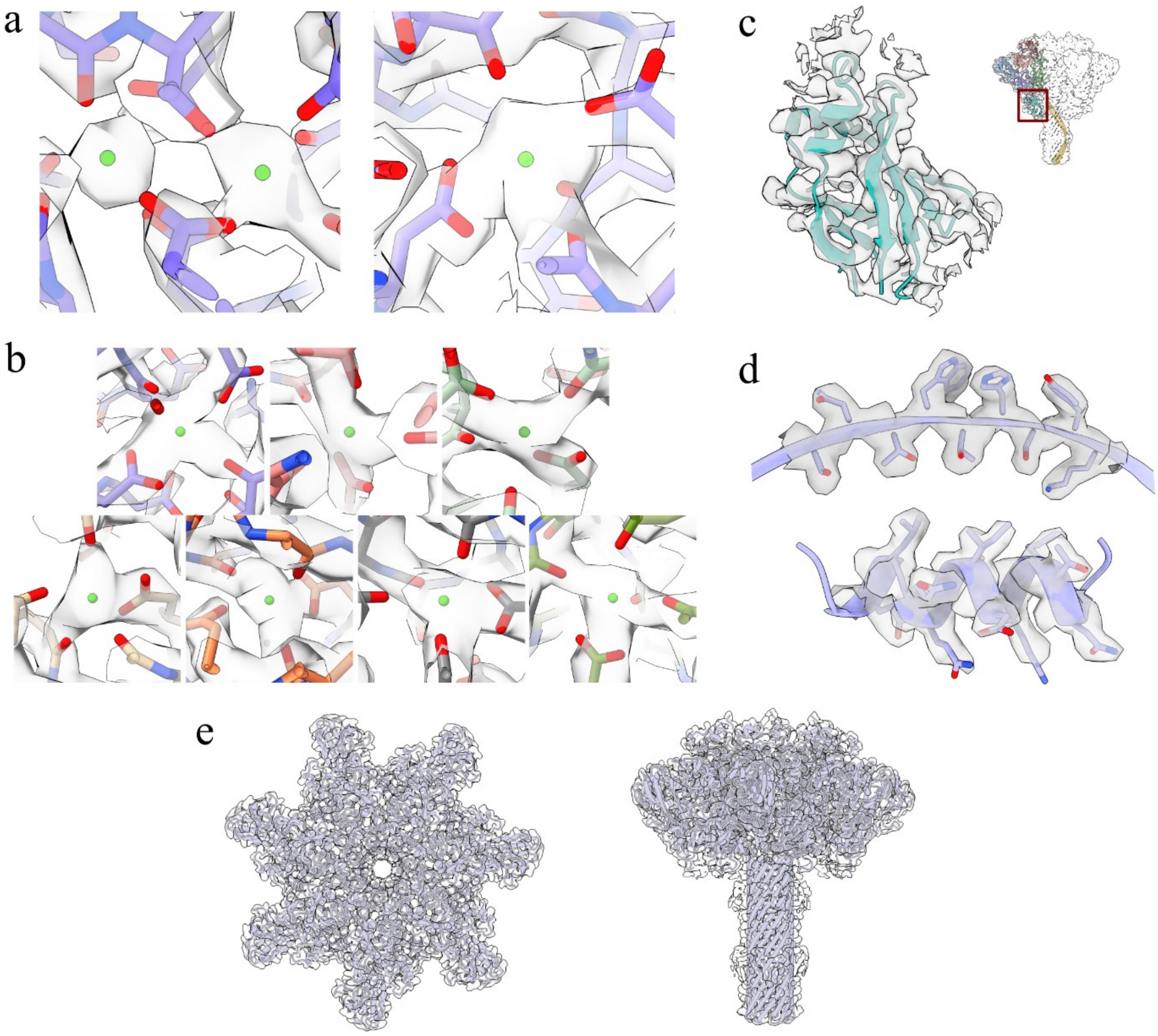

Supplementary Figure 9 Local density of select regions of the Vpb4Aa2. a) Local density of calcium ions (in green) on domain 1 (left) and domain 4 (right). **b**) Local density of calcium ions on domain 4 of each subunit on the C1 reconstruction of Vpb4Aa2 pore. **c)** Fitting of the AlphaFold-modelled domain 6 into the map density. **d)** Local density of β-sheet (top, K356-S264) and α-helix (bottom, N594-E604). **e)** Top view (left) and front view (right) of Vpb4Aa2 pore model fit to the sharpened map.

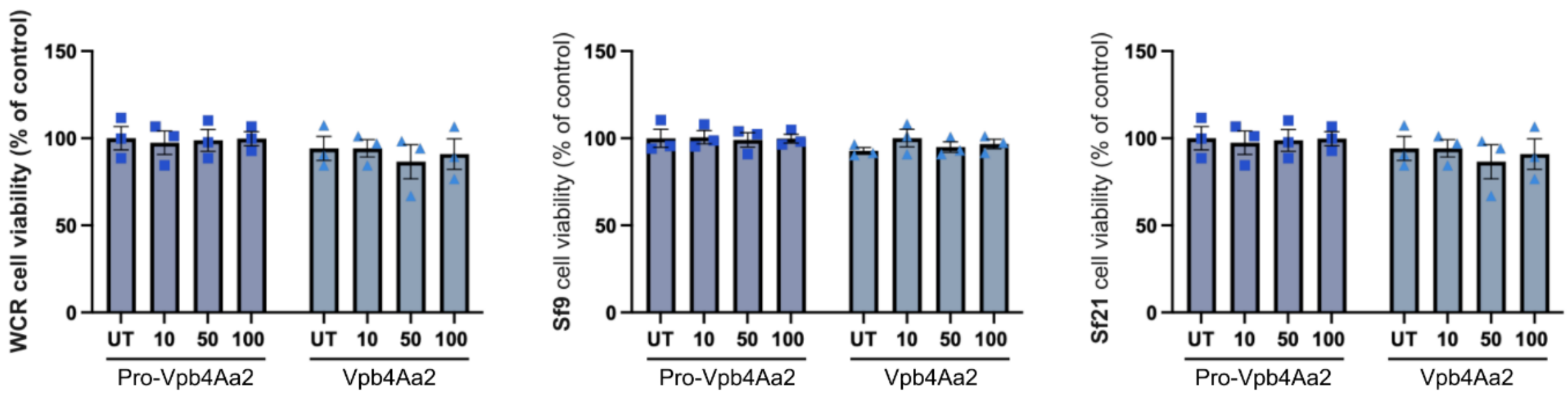

Supplementary Figure 10 Vpb4Aa2 activity assay against insect cells. Cells were treated with a range of concentrations (10, 50 and 100 µg/mL) and survival was measured after 48 h post-treatment, with an amount corresponding to µg/mL. Error bars denote standard error of the mean (n = 3).

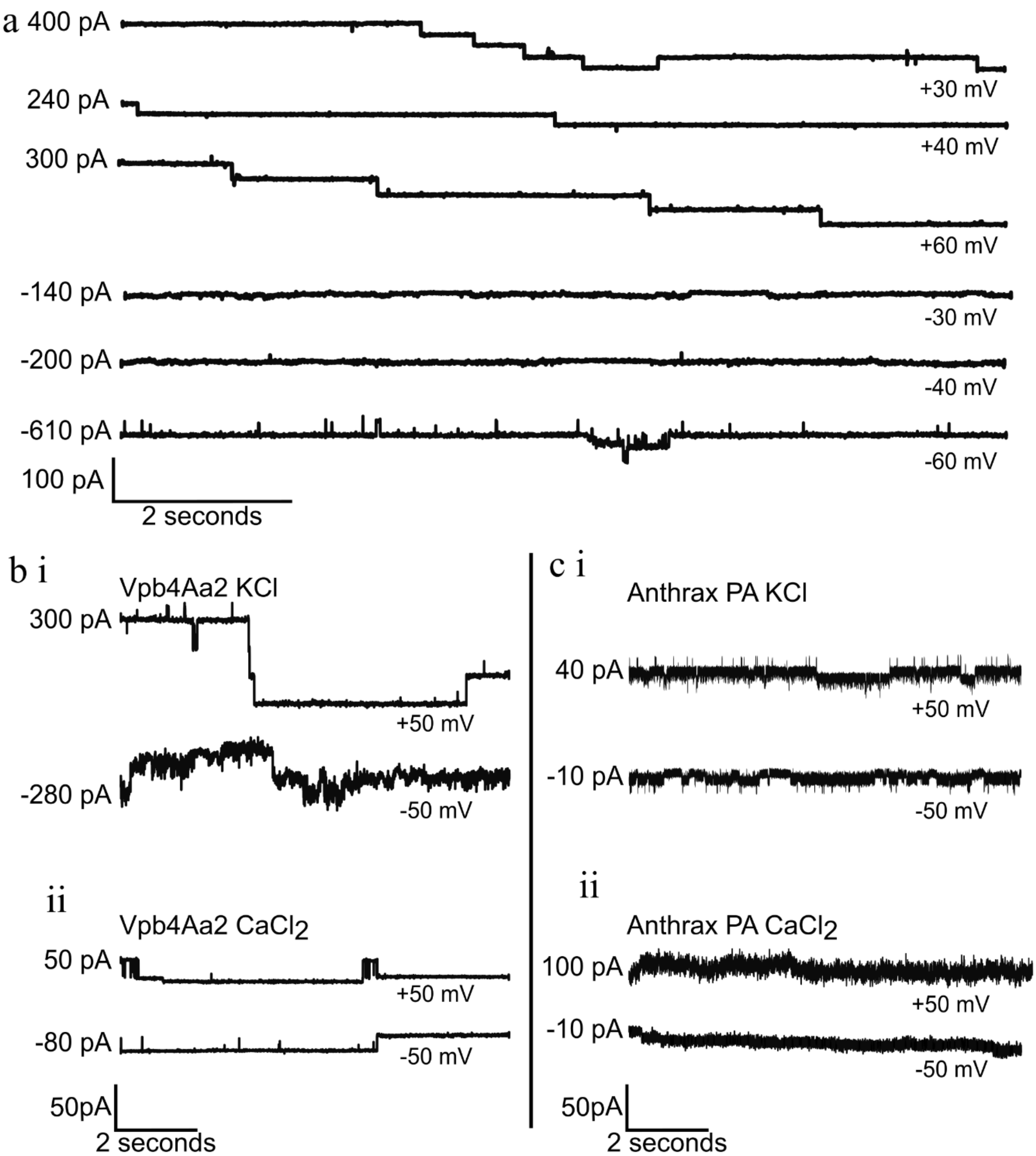

Supplementary Figure 11 Electrophysiology of Vpb4Aa2. a) Representative current traces of Vpb4Aa2 at applied potentials of +30, +40, +60, -30, -40 and -60 mV in 1 M KCl, 10 mM HEPES. **b**) Vpb4Aa2 current traces at +50 and -50 mV in i) KCl and ii) $CaCl_2$ and **c)** comparator Anthrax PA electrophysiology current traces at +50 and -50 mV in i) KCl and ii) $CaCl_2$.

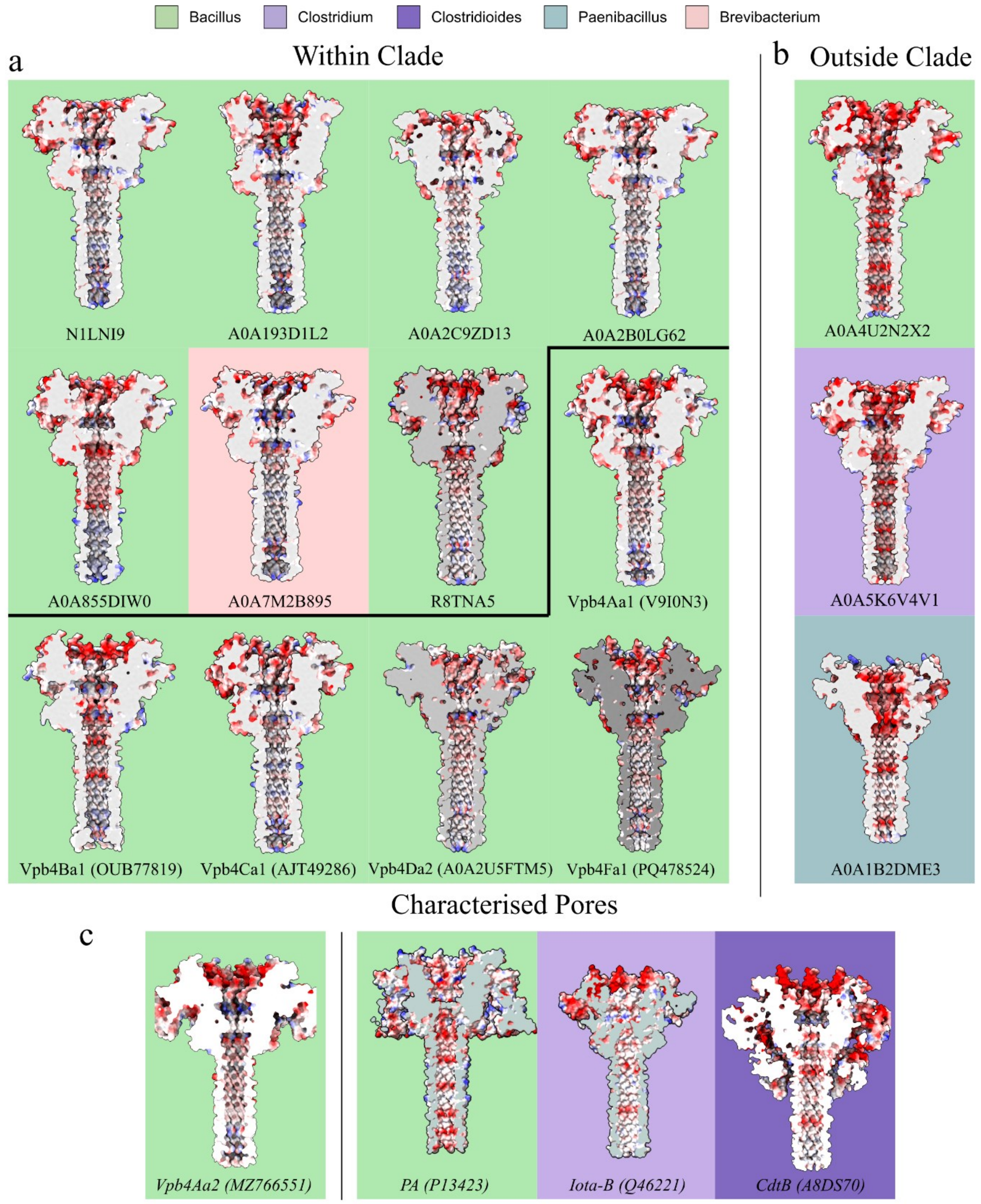

Supplementary Figure 12 Cross-section of the pore lumens of putative Bacterial_Exotoxin_B without canonical ϕ-clamps. Non-canonical pores are grouped **a)** within the Vpb4-like clade and **b)** outside the Vpb4-like clade. **c)** Structurally characterised pores of the Bacterial_Exotoxin_B (PA, Iota-B, and CdtB) members are included for comparisons. Backgrounds are coloured based on genus.

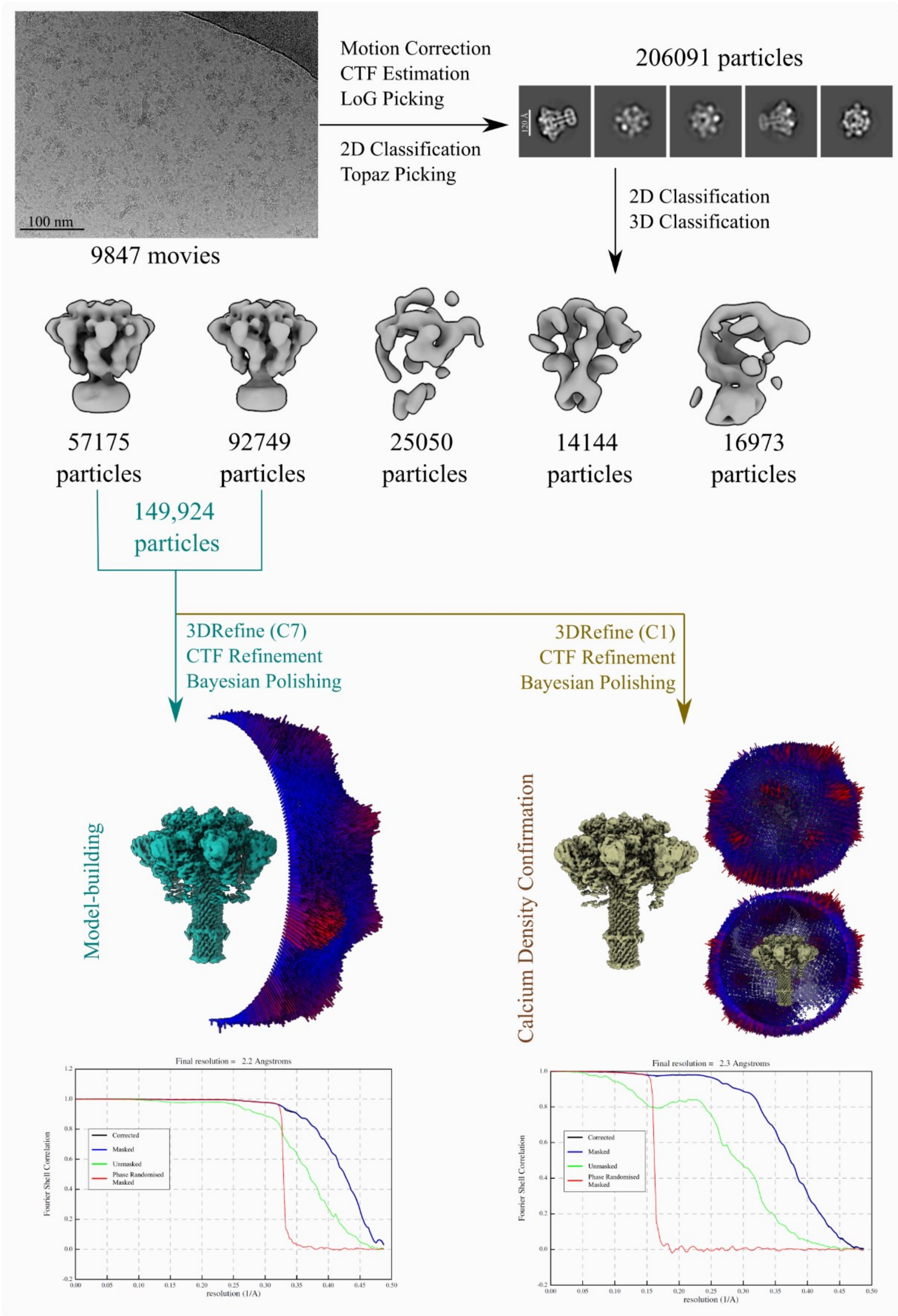

Supplementary Figure 13 Cryo-EM single particle reconstruction workflow. Single particle analysis was performed on RELION 5.0. Two reconstructions were performed on a single dataset, yielding a 2.2 Å structure of Vpb4Aa2 pore at C7 symmetry for model-building and 2.3 Å structure of Vpb4Aa2 pore at C1 symmetry for confirmation of calcium ions.

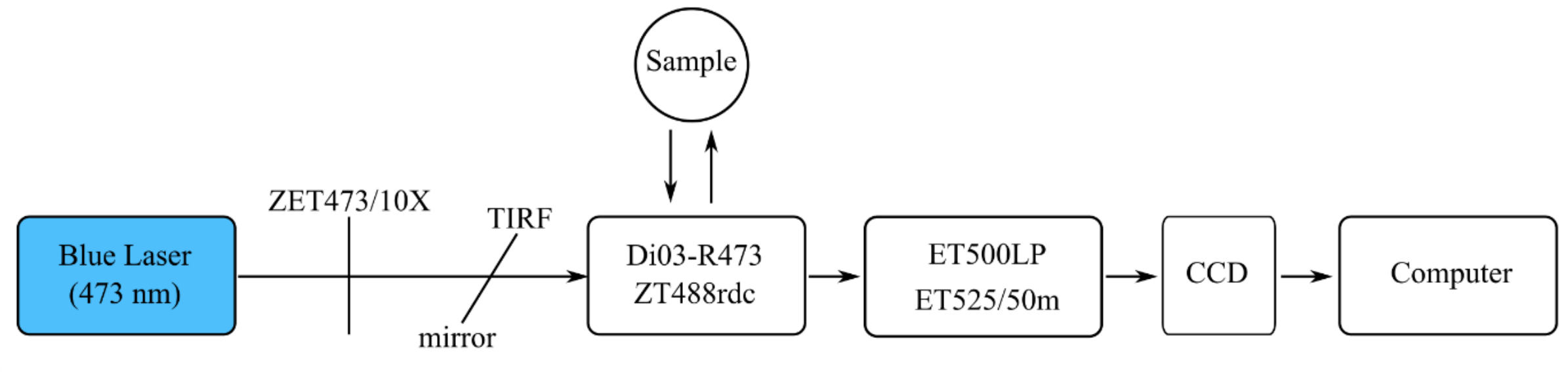

Supplementary Figure 14 Optics setup for Ca^{2+} flux under TIRF microscope as described in TIRF microscopy methods.

Supplementary Table 1 Statistics for reconstruction and refinement. Model building statistics were generated in Phenix 1.20.

	**Pore**	**Monomer**
**Data collection**		
Voltage (kV)	300	
Electron exposure (e^-/Å^2)	60	
Defocus range (µm)	1300 - 3000	
Pixel Size	0.82	
Spherical aberrations (mm)	2.7	
Particle images (#)	149924	
Symmetry imposed	C7	
Map resolution (0.147 FSC threshold)	2.2	
**Model Composition**		
Non-hydrogen atoms	35021	5470
Protein residues	4438	729
Ligands	21	3
**B-factors (Å^2)**		
Protein	17.80/111.77/38.28	15.28/124.35/41.82
Ligand	25.95/40.76/32.57	26.52/39.09/31.86
**Bonds (RMSD)**		
Lengths (Å)	0.007	0.006
Angles (°)	1.356	1.002
**Ramachandran plot (%)**		
Favoured	97.78	96.04
Allowed	2.22	3.06
Disallowed	0.00	0.00
**Validation**		
MolProbity score	0.95	1.04
Clashscore	1.53	1.34
Poor rotamers (%)	0.00	0.36

Supplementary Table 2 PDBePISA interface analysis of two Vpb4Aa2 pore subunits. Residues labelled with a single asterisk (*) is from D1, and double asterisk (**) is from D2.

HYDROGEN BONDS		
**Structure 1**	**Dist (Å)**	**Structure 2**
TYR 261[OH]*	3.62	ASN 238[OD1]*
LYS 262[NZ]*	2.77	GLY 278[O]*
ARG 264[NH2]*	2.83	THR 496[O]**
ARG 264[NH2]*	3.15	THR 496[OG1]**
PHE 351[N]**	2.88	TRP 344[O]**
PHE 353[N]**	2.83	LEU 342[O]**
TYR 357[N]**	2.77	LEU 338[O]**
HIS 359[N]**	2.90	VAL 336[O]**
TRP 361[N]**	2.73	ASN 334[O]**
THR 362[OG1]**	3.44	THR 333[OG1]**
ASN 363[N]**	2.82	ASN 332[O]**
THR 365[N]**	2.81	ASN 330[O]**
ILE 367[N]**	2.84	ASP 328[O]**
GLN 368[NE2]**	2.83	THR 327[OG1]**
ASN 369[N]**	2.74	VAL 326[O]**
SER 370[OG]**	3.37	LYS 324[O]**
GLU 371[N]**	2.79	LYS 324[O]**
GLU 373[N]**	2.72	MET 322[O]**
SER 374[OG]**	2.74	THR 321[OG1]**
TRP 375[N]**	2.91	GLY 320[O]**
SER 376[OG]**	3.51	SER 319[OG]**
SER 377[N]**	2.79	SER 318[O]**
VAL 379[N]**	2.84	GLU 316[O]**
ILE 381[N]**	2.77	VAL 314[O]**
ASN 817[ND2]	3.69	LEU 673[O]
TYR 261[O]*	3.42	GLN 239[NE2]*
PRO 269[O]*	3.12	GLN 498[NE2]**
PHE 351[O]**	2.74	TRP 344[N]**
GLU 352[OE1]**	3.11	LYS 341[NZ]**
PHE 353[O]**	2.80	LEU 342[N]**
PRO 355[O]**	2.91	ALA 340[N]**
TYR 357[O]**	2.73	LEU 338[N]**
HIS 359[O]**	2.89	VAL 336[N]**
TRP 361[O]**	2.71	ASN 334[N]**
ASN 363[O]**	2.72	ASN 332[N]**
THR 365[O]**	2.82	ASN 330[N]**
ILE 367[O]**	2.74	ASP 328[N]**
ASN 369[O]**	2.73	VAL 326[N]**
GLU 371[O]**	2.76	LYS 324[N]**
GLU 371[OE1]**	3.58	LYS 324[NZ]**
GLU 373[O]**	2.81	MET 322[N]**
TRP 375[O]**	2.77	SER 319[OG]**
TRP 375[O]**	3.01	GLY 320[N]**
SER 377[O]**	2.78	SER 318[N]**
SER 377[O]**	3.88	SER 318[OG]**
VAL 379[O]**	2.90	GLU 316[N]**
ILE 381[O]**	2.70	VAL 314[N]**
GLU 385[OE2]**	2.73	LYS 461[NZ]**
SER 420[O]**	3.00	ASN 390[ND2]**
SER 420[OG]**	3.15	SER 450[N]**

ASN 382[ND2]**	2.98	ASN 313[OD1]**	LEU 421[O]**	3.46	ASN 390[ND2]**
ASN 382[ND2]**	2.87	ASN 311[OD1]**	ALA 454[O]**	2.69	ASN 429[ND2]**
SER 383[N]**	2.75	GLU 312[O]**	GLU 482[OE1]**	2.73	ALA 447[N]**
SER 383[OG]**	2.75	GLU 312[OE1]**	THR 483[O]**	2.72	GLN 446[NE2]**
ALA 384[N]**	3.16	LYS 310[O]**	GLN 485[OE1]**	2.58	ASN 433[N]**
ASN 411[ND2]**	3.24	ALA 447[O]**	ASN 513[OD1]**	3.43	TYR 404[OH]**
ASN 411[ND2]**	2.71	PRO 448[O]**	ASN 513[OD1]**	3.21	ALA 283[N]*
THR 423[N]**	2.77	SER 450[OG]**	ASP 542[OD2]	2.89	LYS 564[NZ]
THR 423[OG1]**	3.74	ASN 429[O]**	ASP 543[OD2]	2.69	ARG 289[NH2]*
THR 425[N]**	3.06	ASN 429[OD1]**	GLU 545[OE2]	2.70	GLN 239[N]*
THR 425[OG1]**	2.83	PRO 428[O]**	GLU 545[OE2]	2.65	ASN 238[N]*
ASN 455[ND2]**	2.73	ALA 457[O]**	ASN 817[OD1]	3.75	THR 672[OG1]
ASN 465[ND2]**	3.68	PHE 308[O]**	**SALT BRIDGES**		
ASN 465[ND2]**	2.94	SER 309[OG]**	**Structure 1**	**Dist (Å)**	**Structure 2**
THR 484[OG1]**	2.82	TYR 441[OH]**	LYS 356[NZ]**	3.63	ASP 337[OD1]**
GLN 485[NE2]**	2.75	ILE 431[O]**	GLU 352[OE1]**	3.11	LYS 341[NZ]**
LYS 487[N]**	3.11	ASN 433[OD1]**	GLU 352[OE2]**	3.48	LYS 341[NZ]**
TYR 490[OH]**	3.74	LEU 499[O]**	GLU 371[OE1]**	3.58	LYS 324[NZ]**
THR 512[OG1]**	3.82	SER 434[O]**	GLU 385[OE2]**	2.73	LYS 461[NZ]**
ASN 540[ND2]	3.66	GLU 286[OE1]*	ASP 542[OD2]	2.89	LYS 564[NZ]
ASN 594[ND2]	3.64	SER 575[O]	ASP 543[OD2]	2.69	ARG 289[NH2]*
LYS 597[NZ]	2.93	ASN 576[O]			

Supplementary Table 3 PDBePISA Average subunit-subunit interface and ΔG value among the structurally characterised pore structure of the family members. Only the pore-forming domains (D1-3) were used for PDBePISA interface analysis for consistency.

Protein	PDB	Interface Area ($Å^2$)	$\Delta_i G$ (kcal/mol)	Description
Vpb4Aa2	9PHF	3465.3	-8.5	Domain 4 and 5 removed
PA	7KXR	3493.8	-4.0	Domain 4 and LF removed
Iota-B	6KLW	3152.0	-8.3	Iota-A removed
CdtB	6O2N	3164.9	-12.1	Non-β-barrel CdtB heptamer removed

Supplementary Table 4 Displayed conductance value of Vpb4Aa2 and PA in KCl and $CaCl_2$ environment.

	Vpb4Aa2 (nS)		PA (nS)	
	+	-	+	-
KCl	0.59 ± 0.041	0.55 ± 0.076	0.15 ± 0.016	0.17 ± 0.024
$CaCl_2$	0.46 ±0.044	0.31 ± 0.008	0.084 ± 0.024	0.062 ± 0.021

Supplementary Table 5 Summary of accession codes for characterised Bacterial_Exotoxin_B members, identified Vpb4 subclass members, and other proteins used in the study. For the phylogenetic analysis, NCBI GenBank accession codes were listed for proteins without UniProt entries. Structures of Vpb4Aa2 (with asterisk) were determined in this study

Protein	PDB Accession	UniProt Accession	NCBI GenBank Accession
PA	7KXR (Pore)<br>6UZB (Pore, SF 5)	P13423	-
Iota-B	6KLW (Pore)	Q46221	-
Cdtb	6O2N (Pore)<br>7VNN (Pore, SF 5)	A8DS70	-
C2-II	-	O86171	-
Vpb1Ac1	6SMS (Pro-monomer)	-	AEH05932
Vpb4Aa1	-	V9I0N3	-
Vpb4Aa2*	9PHF (Pore)<br>22ZO (Pore Monomer)	-	MZ766551
Vpb4Ba1	-	-	OUB77819
Vpb4Ca1	-	A0A2U5GYP0	-
Vpb4Da2	7MJR (Pro-monomer)	A0A2U5FTM5	-
Vpb4Fa1	-	-	PQ478524
Aerolysin	9FM6 (Pore)	-	-
Alpha-hemolysin	9KG6 (Pore)	-	-
FIP-fve	1OSY	-	-
FIP-nha	8GO5	-	-

Supplementary Table 6 Summary of accession codes and metadata for AFDB hits by FoldSeek used in the study. Tables are sorted based on sequence identity to the pore-forming domains (D1-3) of Vpb4Aa2.

Target	Origin	Prob.	Seq. Id.	TM-Score	Pos
A0A7M2B895	*Brevibacterium sp. JNUCC-42*	1	60.8	0.763	1-586
R8TNA5	*Bacillus cereus VD184*	0.97	54.3	0.805	1-522
A0A2B0LG62	*Bacillus cereus*	1	54.1	0.723	1-586
A0A2C9ZD13	*Bacillus thuringiensis serovar iberica*	1	53.8	0.736	1-586
N1LNI9	*Bacillus sp. GeD10*	1	53.6	0.761	1-586
A0A855DIW0	*Bacillus thuringiensis*	1	49.9	0.765	1-586
A0A193D1L2	*Bacillus thuringiensis serovar coreanensis*	0.92	47	0.743	1-459
A0A243IS14	*Bacillus thuringiensis serovar konkukian*	1	43	0.724	1-586
A0A2B9U1K5	*Bacillus cereus*	1	42.9	0.772	1-586
A0A1Q1NKN7	*Bacillus thuringiensis*	1	40.8	0.791	1-586
A0A243J249	*Bacillus thuringiensis serovar konkukian*	1	40.1	0.861	1-586
A0A4U2N2X2	*Bacillus wiedmannii*	1	39.3	0.763	1-586
A0A1B2DME3	*Paenibacillus sp. BIHB4019*	0.99	38.5	0.768	1-586
A0A1J9W975	*Bacillus anthracis*	1	37	0.717	1-586
A0A5K5I9R1	*Bacillus sp. FDAARGOS_235*	0.99	36.8	0.748	1-586
A0A6L5MPH7	*Bacillus thuringiensis*	1	35.2	0.745	5-586
A0A1L8WHH5	*Enterococcus ratti*	1	33.7	0.749	1-586
A0A854DEK1	*Bacillus thuringiensis*	0.99	33.6	0.845	1-586
A0A5K6V4V1	*Clostridium perfringens*	1	33.2	0.776	3-586
A0A2C1I5G7	*Bacillus cereus*	0.99	32.8	0.762	1-586
A0A243E466	*Bacillus thuringiensis serovar toumanoffi*	0.99	32.6	0.782	1-586
A0A7Z1FUE8	*Bacillus pseudomycoides*	0.99	32.3	0.71	4-586
A0A1Q9ICI3	*Bacillus cereus*	1	31.8	0.777	2-586

A0A1S9YMT3	*Bacillus cereus*	1	31.8	0.818	1-586
A0A854KS94	*Bacillus thuringiensis serovar shandongiensis*	0.99	31.8	0.764	3-586
A0A3S0ZSZ8	*Bacillus sp. VKPM B-3276*	0.99	31.6	0.698	1-586
A0A2K8U1B3	*Bacillus cereus*	1	31.5	0.827	2-586
A0A242WVX5	*Bacillus thuringiensis serovar cameroun*	0.99	31.4	0.731	5-586
A0A2S5GZH4	*Brevibacillus laterosporus*	0.94	31.1	0.775	54-545
R8GEK9	*Bacillus cereus VD196*	0.99	30.4	0.766	1-586
A0A0J7J958	*Bacillus cereus*	1	30.3	0.742	1-586
A0A1U9YTG9	*Paenibacillus larvae subsp. pulvifaciens*	0.99	30.3	0.805	1-586
A0A854Z0I7	*Bacillus cereus*	0.99	30.1	0.717	5-586
A0A848MZX0	*Enterococcus mundtii*	0.99	30.1	0.746	3-586
W2EBZ5	*Paenibacillus larvae subsp. larvae DSM 25719*	0.95	30.1	0.757	1-510
R8GJC6	*Bacillus cereus VD196*	0.99	29.9	0.733	1-586
H3SHP6	*Paenibacillus dendritiformis C454*	0.99	29.8	0.729	3-586
A0A2I4NN04	*Clostridium botulinum*	0.99	29.7	0.725	1-586
A0A4U2NUL3	*Bacillus cereus*	0.99	29	0.763	1-586
A0A3G2Y2S5	*Bacillus cereus*	0.99	28.4	0.794	1-586
A0A4Y5USZ9	*Paenibacillus larvae*	0.84	28.3	0.647	86-586
A0A4U2NJP7	*Bacillus cereus*	0.96	27.7	0.789	31-586
R8DKN2	*Bacillus cereus VD133*	0.99	27.3	0.741	1-586
A0A5C1FNS9	*Bacillus sp. JAS24-2*	0.99	27.3	0.663	1-586
C2Q554	*Bacillus mycoides*	0.99	27.2	0.756	1-586
A0A2A5JX56	*Paenibacillus larvae subsp. larvae B-3650*	0.95	27.2	0.639	34-586
A0A1S9TH11	*Bacillus cereus*	0.99	27.1	0.781	1-586
A0A4U3AK25	*Bacillus mycoides*	0.98	26.2	0.763	51-586
A0A1U9YPL5	*Paenibacillus larvae subsp. pulvifaciens*	0.97	26.1	0.803	2-576

W2ECV5	*Paenibacillus larvae subsp. larvae DSM 25719*	0.99	24.2	0.693	2-576
