# Supplementary material for: Structural basis for independent pore function of Vpb4 from Bacillus thuringiensis
Source: Nat Commun. 2026 Jul 1;17:8198. doi: 10.1038/s41467-026-74567-y (PMC13462110; doi:10.1038/s41467-026-74567-y)
Supplement: Supplementary file 2 [file 41467_2026_74567_MOESM2_ESM.pdf]

Description of Additional Supplementary Files

File Name: Supplementary Movie 1

Description: Domain rearrangement of Vpb4Aa2 before and after pore formation. The rearrangement was modelled using the morph command in ChimeraX 1.10.1. Pro-Vpb4Da2 crystal structure was used as a Vpb4 representative to morph into the full-length Vpb4Aa2 monomer.

File Name: Supplementary Movie 2

Description: Raw movies for Ca^{2+} flux experiments revealed three individual pores. Videos were taken through the optical setup in Supplementary Figure 14.
