# Supplementary material for: Structural basis for independent pore function of Vpb4 from Bacillus thuringiensis
Source: Nat Commun. 2026 Jul 1;17:8198. doi: 10.1038/s41467-026-74567-y (PMC13462110; doi:10.1038/s41467-026-74567-y)
Supplement: Supplementary file 3 [file 41467_2026_74567_MOESM5_ESM.pdf]

nature portfolio

Corresponding author(s): Bradley Spicer, Oliver Castell

Last updated by author(s): Apr 23, 2026

Reporting Summary

Nature Portfolio wishes to improve the reproducibility of the work that we publish. This form provides structure for consistency and transparency in reporting. For further information on Nature Portfolio policies, see our Editorial Policies and the Editorial Policy Checklist.

Statistics

For all statistical analyses, confirm that the following items are present in the figure legend, table legend, main text, or Methods section.

n/a	Confirmed	
☐	☒	The exact sample size (*n*) for each experimental group/condition, given as a discrete number and unit of measurement
☐	☒	A statement on whether measurements were taken from distinct samples or whether the same sample was measured repeatedly
☒	☐	The statistical test(s) used AND whether they are one- or two-sided<br>*Only common tests should be described solely by name; describe more complex techniques in the Methods section.*
☒	☐	A description of all covariates tested
☒	☐	A description of any assumptions or corrections, such as tests of normality and adjustment for multiple comparisons
☐	☒	A full description of the statistical parameters including central tendency (e.g. means) or other basic estimates (e.g. regression coefficient) AND variation (e.g. standard deviation) or associated estimates of uncertainty (e.g. confidence intervals)
☒	☐	For null hypothesis testing, the test statistic (e.g. F, t, r) with confidence intervals, effect sizes, degrees of freedom and P value noted<br>*Give P values as exact values whenever suitable.*
☒	☐	For Bayesian analysis, information on the choice of priors and Markov chain Monte Carlo settings
☒	☐	For hierarchical and complex designs, identification of the appropriate level for tests and full reporting of outcomes
☒	☐	Estimates of effect sizes (e.g. Cohen's *d*, Pearson's *r*), indicating how they were calculated

Our web collection on statistics for biologists contains articles on many of the points above.

Software and code

Policy information about availability of computer code

Data collection	Mass Photometry: AcquireMP v2024R1 (Refeyn).<br>Size Exclusion Chromatography curve: Unicorn v5.31 (Cytiva).<br>Cryo-EM movies: EPU Software v2.12.1.2782 (Thermo Fisher).<br>TIRF Microscopy: Andor Solis I Software v4.29.300012.0 (Oxford Instruments).<br>Electrophysiology current: WinEDR v4.0.4 (university of Strathclyde).
Data analysis	Gene annotation: Prodigal v2.6.3, NCBI blastx (https://blast.ncbi.nlm.nih.gov/Blast.cgi), IGV-Web (https://igv.org/app).<br>General graphs: GraphPad Prism v10.4.1<br>Mass photometry: DiscoverMP v2024R1<br>Molecular Visualisation and diameter measurement: ChimeraX v1.10.1, HOLE2 v2.3.1<br>Cryo-EM processing: RELION-5.0, CTFFIND4, MotionCor3, EMReady v2.0, WinCoot v0.9.8, Phenix v1.20<br>TIRF Microscopy and Electrophysiology: Matlab vR2023b, FIJI imageJ v1.54p, WinEDR v4.0.4<br>Phylogeny: MAFFT v7.526, trimAL v1.5, IQ-TREE v3.0

For manuscripts utilizing custom algorithms or software that are central to the research but not yet described in published literature, software must be made available to editors and reviewers. We strongly encourage code deposition in a community repository (e.g. GitHub). See the Nature Portfolio guidelines for submitting code & software for further information.

Data

Policy information about availability of data

All manuscripts must include a data availability statement. This statement should provide the following information, where applicable:

- Accession codes, unique identifiers, or web links for publicly available datasets
- A description of any restrictions on data availability
- For clinical datasets or third party data, please ensure that the statement adheres to our policy

The Vpb4Aa2 pore complex in C7 symmetry model and map have been deposited in the PDB database under accession code 9PHF [https://doi.org/10.2210/pdb9phf/pdb] (pdb_00009PHF) and in the EMDB database under accession code EMD-71647 [https://www.ebi.ac.uk/emdb/EMD-71647]. The single full-length subunit of the Vpb4Aa2 pore complex model has been deposited in the PDB database under accession code 22ZO [https://doi.org/10.2210/pdb22zo/pdb] (pdb_000022ZO). The C1 reconstruction of the pore has been deposited in the EMDB database under accession code EMD-68793 [https://www.ebi.ac.uk/emdb/EMD-68793] in EMDB. All models from other publications used for structural comparison in this study are summarised in Supplementary Table 5 are available in the PDB database under accession codes 7KXR [https://doi.org/10.2210/pdb7kxr/pdb], 6UZB [https://doi.org/10.2210/pdb6uzb/pdb], 6KLW [https://doi.org/10.2210/pdb6klw/pdb], 6O2N [https://doi.org/10.2210/pdb6o2n/pdb], 7VNN [https://doi.org/10.2210/pdb7vnn/pdb], 6SMS [https://doi.org/10.2210/pdb6sms/pdb], 7MJR [https://doi.org/10.2210/pdb7mjr/pdb], 9FM6 [https://doi.org/10.2210/pdb9fm6/pdb], 9KG6 [https://doi.org/10.2210/pdb9kg6/pdb], 1OSY [https://doi.org/10.2210/pdb1osy/pdb], and 8GO5 [https://doi.org/10.2210/pdb8go5/pdb]. Protein accession codes are available in the Uniprot database for P13423 [https://www.uniprot.org/uniprotkb/P13423/entry], Q46221 [https://www.uniprot.org/uniprotkb/Q46221/entry], A8DS70 [https://www.uniprot.org/uniprotkb/A8DS70/entry], O86171 [https://www.uniprot.org/uniprotkb/O86171/entry, V9I0N3 [https://www.uniprot.org/uniprotkb/V9I0N3/entry], A0A2U5GYP0 [https://www.uniprot.org/uniprotkb/A0A2U5GYP0/entry], and A0A2U5FTM5 [https://www.uniprot.org/uniprotkb/A0A2U5FTM5/entry]; and NCBI GenBank for AEH05932 [https://www.ncbi.nlm.nih.gov/protein/AEH05932], MZ766551 [https://www.ncbi.nlm.nih.gov/nuccore/MZ766551], OUB77819 [https://www.ncbi.nlm.nih.gov/protein/OUB77819], and PQ478524 [https://www.ncbi.nlm.nih.gov/nuccore/PQ478524]. All chromatography data, uncropped gels, Hole2 measurement data, and cell-based assay data and minimum data for electrophysiology graphs (single I/V relationship and conductance comparisons) are provided in the Source Data file. All raw data for electrophysiology experiments were deposited in the Zenodo repository [10.5281/zenodo.19552438].

Research involving human participants, their data, or biological material

Policy information about studies with human participants or human data. See also policy information about sex, gender (identity/presentation), and sexual orientation and race, ethnicity and racism.

Reporting on sex and gender	N/A
Reporting on race, ethnicity, or other socially relevant groupings	N/A
Population characteristics	N/A
Recruitment	N/A
Ethics oversight	N/A

Note that full information on the approval of the study protocol must also be provided in the manuscript.

Field-specific reporting

Please select the one below that is the best fit for your research. If you are not sure, read the appropriate sections before making your selection.

[x] Life sciences [ ] Behavioural & social sciences [ ] Ecological, evolutionary & environmental sciences

For a reference copy of the document with all sections, see nature.com/documents/nr-reporting-summary-flat.pdf

Life sciences study design

All studies must disclose on these points even when the disclosure is negative.

Sample size	Electrophysiological data collection and calcium flux imaging was based on the stochastic nature of pore insertion into artificial bilayers and the observational nature of single-molecule recordings. In all experiments of these types multiple experiments were carried out to identify reproducible reconstitution and routine channel recording conditions prior to gathering the displayed data. Calcium flux fluorescence imaging data (Fig 5c and 5d) represents 3 examples of individual pores. For I/V relationships, current values were recorded during four separate voltage application cycles within a single bilayer to ensure comparable measurement at different voltages on the same protein population. Each applied voltage is within a full cycle lasting 15 seconds of which 13 seconds representing ~130196 measurements was harvested. For conductance comparisons between different pores (Fig6e), data were extracted from three independent experiments. The number of analysed events per experiment varied due to the stochastic nature of insertion events but was sufficient to represent characteristic behaviour observed consistently across repetitions. Exemplar traces demonstrating representative pore behaviours (Fig6c, 6d, S11A, S11b, S11c) under different voltage and salt conditions were extracted from the same group of experiments. No sample size calculations were performed for cell-based assays
Data exclusions	Data were excluded from quantitative summaries (e.g., conductance and I/V analyses) when bilayers contained large numbers of simultaneously active pores, which hindered clear resolution of individual pore behaviour. Due to the stochastic properties of the system when working at low number of protein channels, data exclusions were made during analysis where conductance steps indicative of individual

	pore insertions or closings could no longer be confidently distinguished. No other data exclusions reported in the study.
Replication	Protein preparation and activation was performed multiple times for the purpose of the study. Cell viability assays were independently repeated three times using separate viles of cull culture batches. Reagents for culture were obtained from the same supplier and - where possible - lots to minimise variability. All replication experiments were successful with consistent outcomes obtained across experiments.<br><br>Electrophysiology findings were verified through both internal and external replication, together with a thorough process of measurements across a range of protein concentrations to establish the reconstitution regime for single molecule analysis, whilst also confirming repeatability of protein insertion, measurement and behaviour by both electrophysiology and imaging, before embarking on bone fide data collection. Internal replication was performed within experiments via repeated voltage application protocols and/or image acquisitions. Where feasible, experiments were also independently repeated (typically three times). Due to the stochastic nature of pore insertion, cross-experiment comparisons were sometimes limited in interpretability (e.g. due to differing numbers of reconstituted protein pores). Nonetheless, for all observations included in the manuscript, key behaviours were reproducibly observed under comparable experimental conditions, and representative traces have been selected accordingly.
Randomization	For electrophysiology dataset, allocation of pores into experimental groups was not applicable in the traditional sense, as the data were observational and based on stochastic insertion of single molecules into bilayers. In comparisons between different pores, experimental conditions (e.g., bilayer composition, voltage application protocol) were held constant, and data were grouped based on pore type. As such, covariates were controlled through consistent experimental conditions rather than randomisation. No other randomization reported in the study.
Blinding	Blinding was not employed during data collection or analysis. Given the experimental design involves sample preparation by same personnel, real-time observation, and the need for operator intervention during each experiments, blinding was not feasible. However, efforts were made to minimise bias by predefined protocols and criteria for the electrophysiology experiments and automated imaging and analysis of fluorescent output signal for the viability assays.

Reporting for specific materials, systems and methods

We require information from authors about some types of materials, experimental systems and methods used in many studies. Here, indicate whether each material, system or method listed is relevant to your study. If you are not sure if a list item applies to your research, read the appropriate section before selecting a response.

Materials & experimental systems

n/a	Involved in the study
[x]	[ ] Antibodies
[ ]	[x] Eukaryotic cell lines
[x]	[ ] Palaeontology and archaeology
[x]	[ ] Animals and other organisms
[x]	[ ] Clinical data
[x]	[ ] Dual use research of concern
[x]	[ ] Plants

Methods

n/a	Involved in the study
[x]	[ ] ChIP-seq
[x]	[ ] Flow cytometry
[x]	[ ] MRI-based neuroimaging

Eukaryotic cell lines

Policy information about cell lines and Sex and Gender in Research

Cell line source(s)	Sf9 (11496015, Thermo Fisher) and Sf21 (11497013, Thermo Fisher) from Spodoptera frugiperda<br>DvWL2 (USDA, ARS Biological Control of Insects Research Laboratory) from Diabrotica virgifera virgifera
Authentication	The cells were not authenticated.
Mycoplasma contamination	The cells were not tested for mycoplasma contamination.
Commonly misidentified lines<br>(See ICLAC register)	No commonly misidentified cell lines were used.

Plants

Seed stocks	N/A
Novel plant genotypes	N/A
Authentication	N/A
