# Supplementary material for: Structural basis for independent pore function of Vpb4 from Bacillus thuringiensis
Source: Nat Commun. 2026 Jul 1;17:8198. doi: 10.1038/s41467-026-74567-y (PMC13462110; doi:10.1038/s41467-026-74567-y)
Supplement: Supplementary file 4 [file 41467_2026_74567_MOESM6_ESM.pdf]

nature portfolio

Peer Review File

Structural basis for independent pore function of Vpb4 from Bacillus thuringiensis

Corresponding Author: Mr Raymond Wirawan

This file contains all reviewer reports in order by version, followed by all author rebuttals in order by version.

Version 0:

Reviewer comments:

Reviewer #1

(Remarks to the Author)
The manuscript by Wirawan et al. describes structural and some functional characterization of the bacterial toxin Vpb4Aa2 from B.thuringiensis. The protein can be classified based on its sequence and structure as a member of the bacterial exotoxin family highlighted by anthrax PA. However, well characterized bacterial toxins of the family are known to act as multiprotein complexes that include the cell binding protein translocase structurally homologous to Vpb4Aa2 and one or more “active” protein components, usually enzymes that when translocated into cytosol disrupt the cell in some way, leading to cell death. The target of the study presented in the manuscript is different in the sense that it is posited to provide significant toxicity without an active component, and authors suggest that the mechanism of toxicity is based simply on the disruption of the cell membrane and that cell death is driven by resulting electrolyte balance disruption.

The main focus of the manuscript is the structure determination of the membrane insertion form of the toxin by means of cryoelectron microscopy. As a structural biologist myself, I certainly appreciate this kind of analysis. Where I think the paper is lacking is the functional studies that could support some of the proposed conclusions, (limited here to some biophysical characterization of pore formation in a lipid membrane - electrophysiology is certainly not my area of expertise, but I am not sure if the included studies can be described as “electrophysiological” given that no cells or tissues were involved).

The most important omission here is proof that the Vpb4Aa2 is, in fact, a toxin. Authors present some cell toxicity studies that, unfortunately, indicate that at least for the insect cell lines tested no toxicity is observed up to 0.1 mg/ml of protein concentration. Perhaps they are right and the cell lines they used lack the proper toxin receptor, but one cannot simply ignore the lack of the evidence that this specific protein is, indeed, insecticidal. Yes, the related protein Vpb4Da2 is reported to have such properties, but with midlevel sequence homology (57% identical) the function would have to be confirmed in some manner.

Furthermore, the assertion that this class of toxins lack the active component in vivo requires some proof as well. Authors should point to some studies that indicate that at least for the Vpb4Da2, at which point they could extend this as a hypothesis to the rest of the toxins that carry similar key structural elements. Note that just because Vpb4Da2 is insecticidal on its own does not prove that it is not binding some yet unknown toxic enzyme in vivo. I may be unaware of studies that provide such proof due to simple ignorance, so if I am mistaken please reference some specific papers that prove that Vpb4Da2 does not, in fact, have a partner enzyme. Sequence variation in the canyon region of the protein only indicates that it will not bind active components of other toxins, and does not prove that no such active component exists.

Perhaps one piece of supportive evidence could be to determine the toxicity of the Vpb4Da2 as the positive control in the cell toxicity assays utilized in the course of this study. It has been demonstrated that this toxin is lethal to western corn rootworm, so determining effective toxin concentration could be useful in order to establish a reference point.

Authors also dismiss standalone toxicity of the other translocases as low level activity. At least for the CDTb toxicity is on the order of 0.5ug/ml. It would be useful to demonstrate that the Vpb4 class toxins have higher toxicity. Otherwise the conclusion perhaps should be that the Vpb4 is not a novel class of toxins that evolved to act without an enzymatic partner, but rather that it represents a simpler descendant of the ancestral translocase that has not yet evolved into the binary toxin. It also appears to be the case that the authors use the argument of the allegedly low standalone toxicity of other translocases to bolster their hypothesis that Vpb4 class proteins insert directly into the cell membrane without the need to trigger the conformational

change in mature endosomes. That may well be the case, but just like with some other conclusions offered, that would require more proof. Another possible approach would be to swap in the receptor binding domain from, say, CDTb and see if such a construct is toxic to Vero cells. This could bolster the argument that Vpb4's are effective toxins by simply piercing the outer cell membrane.

In essence, this is a very nice structural study augmented with some biophysical data on ion channel like properties. I do not have any concerns with that - the study itself is sound and makes some rather interesting observations regarding the beta-barrel geometry. I also find that this study provides an important contribution as it presents the first full length membrane inserted model structure of the anthrax-like bacterial translocase that has 3 receptor binding domains (the term which is rather unfortunate given that it is very likely that two of these do not bind any receptors). As a co-author of the recently published study on the role of endosomal calcium depletion in activation of the CDTb I find it particularly interesting if the authors could present some analysis on the CBM-like domain that appears to retain calcium (from what I understand, it's only detected in C1-refined structure, so it's possible that only some CBM-like domains retain the metal and not all) - perhaps to see whether calcium depletion may be effective in triggering the conformational change or at least to look into structural differences that may explain why calcium switch is not relevant for Vpb4Aa2.

I am not sure if this is feasible, but it would be terrific if one could analyze the co-evolution of binding and active components of various toxins, if the goal is to provide supporting evidence that Vpb4 class of toxins evolved from binary toxins to a standalone hemolysin-like toxins.

To summarize, this is a good study, but based on my past experience with journals such as Nature Communications, it would require a bit more functional studies to justify the proposed impact.

Sincerely,

Ed Pozharski.

Reviewer #2

(Remarks to the Author)
In this manuscript, the authors determined a cryo-EM structure of Bacillus thuringiensis Vpb4Aa2, a recently discovered bacterial toxin, and performed electrophysiology experiments to show its unique characteristics compared to other toxins of the same family. The article is novel, based on well-performed experiments, and well-written.

I have only one major concern and a few minor comments before I can recommend the article for acceptance.

If I haven't missed anything, the authors couldn't show that the protein prep they are working on is toxic to any cells or organisms. While I can understand that this might be due to the high specificity to its receptor, that also can mean that the protein is non-functional due to the method of production or purification. If the latter is true, the following biochemical and structural analysis of such a sample becomes irrelevant. I find it necessary to show that the toxin is toxic at concentrations compared to those of other members of the family.

I thank the authors for sharing raw cryo-EM data and models. I find them of very good quality. Also great that you described methods and added supplementary figures about cryo-EM data collection and processing. I kindly ask to add scale bars to 2D class averages and show viewing direction distribution on Supplementary Figure 13. Finally, to the cryo-EM part, you show that some domains appear flexible and couldn't be resolved to high resolution. I recently had a similar experience but running refinement in Relion 5 with Blush regularization could improve it. I suggest trying it as well; it shouldn't take much time.

Finally, two small textual points:
- Abstract, line 15: Bacterial exotoxin B is a vague name for me. It would also fit for streptococcal pyrogenic exotoxin B or TcdB of C. difficile. Is there an option to call it differently?
- I would suggest using Iota-B instead of IB abbreviation for the C. perfringens toxin.

Version 1:

Reviewer comments:

Reviewer #1

(Remarks to the Author)
The main concern of my initial review was that the manuscript lacked experimental evidence that the protein authors

selected for their structural study is indeed insecticidal (both as capability and true function). The revised version of the manuscript does not address this concern and instead argues in a clearer manner that because the target protein has ~50% identity with another B.thuringiensis protein that was shown to have some level of insecticidal activity it therefore must be an insecticide. I checked and one of the classic binary toxins of the same heptameric translocase class, CDTb, shares about 43% sequence identity with the Vpb4Da2 – but obviously one would not assume that CDTb translocase is a non-binary toxin just based on sequence identity (and obviously is not an insecticidal toxin). Especially in bacteria with its powerful horizontal gene transfer mechanisms, functional conclusions based on sequence alignments are always difficult (and yet admittedly leave a lot of room for various hypotheses, which is obviously exciting).
As I said previously, authors present excellent structural and biophysical data. Yes, the protein forms a channel (not surprising given its homology to bacterial protein translocases). Yes, related proteins appear toxic to insects. What is a leap for me is the proposal that this means that this protein has evolved to be an insect toxin without the need for an enzyme payload. I understand that this could be my reading of what the authors propose, and perhaps I have gone too far.
But if that is indeed the suggestion, then in my mind it would require a bit more evidence. I still see two problems here. Firstly, the protein itself does not show cell toxicity. I understand that the authors believe that this is simply because they didn't stumble across proper target yet. I understand the assumption that all the proteins of this class feature interchangeable receptor binding domains which allows them to switch targets while maintaining core architecture and ability to perforate membranes. Still, what authors show is that the protein can form pores spontaneously without the need for environmental changes or presence of receptor. So why then it is not just toxic across the board to all insects? Isn't it also plausible that it has evolved a different function? In the end it comes down to the fact that structure is there to explain function and cannot be used as proof by itself.
Secondly - perhaps I misunderstand the entire point here, and the focus is on how these proteins might function when used as agricultural tools. Let me explain. When I think of bacterial protein toxins I assume that they are pathogenic to host at concentrations produced by bacteria upon normal colonization. For example, CDT kills humans not only because the translocase component perforates mature endosomes, but because the enzyme destroys cytoskeleton. CDTb by itself is toxic to Vero cells, but in concentrations that are much higher and not practical for the bacteria to produce (but could be useful if one chooses such scaffold as a poison protein). Is it possible that Vpb4 class protein toxicity is not inherent to its evolved function? I understand that Vpb4Da2 was shown to kill WCR, but it's not obvious how effective protein concentration in those experiments compares to in vivo values and background levels of other translocase toxicity.
I also expect that binary toxins evolved from some channel forming protein by acquiring ability to bind enzyme payload, not the other way around. It is fairly obvious that binary toxin system is much more efficient and it's unclear why organism would evolve towards lower toxicity (well, on the other hand, killing the host is not always the bests option). Thus the argument could be that Vpb4 class of proteins might serve as a model of ancestral protein that might have had some limited toxicity, perhaps in addition to its true function. It's an interesting proposition, and given reasonable homology levels across all classes of these translocases, it might be just as likely as reverse evolution.
Also, and correct me here if I am wrong, but B.thuringiensis uses cry proteins as the major toxin against its insect hosts. Unless yet unknown insect target is resistant to cry proteins, it's unclear what would be the evolutionary pressure for evolving new hemolysin style toxins.
I am also surprised that authors could not express the Vpb4Da2. The monomeric form of that protein was determined by crystallography, which often indicates that the protein does not present significant complications when it comes to expression. Could you at least swap in Vpb4Da2 receptor binding domains and test the chimeric protein on WCR?
In summary, the impression one gets from a superficial look at the manuscript is that it discovers example of binary toxin evolution towards non-binary membrane perforating version. I suspect that the reality might be much more complex (but also unfortunately more mundane) than this exciting proposition. It would be great if the author's proposition is correct, but it might require more experimental evidence (starting with demonstrating that the protein they chose is indeed toxic to insects).

Reviewer #2

(Remarks to the Author)
The authors addressed my concerns and majorly improved cryo-EM reconstructions. I recommend the article for publication.

Open Access This Peer Review File is licensed under a Creative Commons Attribution 4.0 International License, which permits use, sharing, adaptation, distribution and reproduction in any medium or format, as long as you give appropriate credit to the original author(s) and the source, provide a link to the Creative Commons license, and indicate if changes were made.
In cases where reviewers are anonymous, credit should be given to 'Anonymous Referee' and the source.
The images or other third party material in this Peer Review File are included in the article's Creative Commons license, unless indicated otherwise in a credit line to the material. If material is not included in the article's Creative Commons license and your intended use is not permitted by statutory regulation or exceeds the permitted use, you will need to obtain permission directly from the copyright holder.
To view a copy of this license, visit https://creativecommons.org/licenses/by/4.0/

Dear Editorial Team,

Thank you for sending us the reviews of our manuscript entitled: 'Structural basis for independent pore function of Vpb4 from Bacillus thuringiensis'. We have modified the manuscript to address the points made by the reviewers and have conducted additional experiments which we believe has enhanced our study.

The major issues raised in the reviews appear to be (i) that it is possible that there is an unidentified second component that could act with Vpb4 proteins (in a similar way to the interactions of the known well characterised enzymatic toxin components such as the Protective Antigen (PA)-like family) and (ii) that we did not identify a specific insect target for the Vpb4 variant that we used in our study. We feel that we have sufficiently addressed these concerns through the rebuttal.

On the first issue of potential secondary components, we accept that this could be possible in theory but unlikely. The accumulative evidence points towards Vpb4 proteins having toxicity towards whole insects when administered alone. We have, furthermore, actively looked for evidence of a candidate partner within the proximity to the Vpb4 gene and do not find any associated genes. Lastly, our structural analysis suggests that Vpb4 proteins do not share structural features for translocation. Beyond this body of evidence, we are unsure how to prove a negative – it is conceivable that a second component, which is not absolutely required for insect killing, may exist elsewhere in the bacterial genome but we have no leads to identify this. Should a cryptic partner protein be discovered in due course, our structure will be key in understanding any interaction and translocation.

Regarding the lack of a known insect target for our protein variant, we have tested against a small range of insect cells, but these were not sensitive. Insecticidal toxins, generally, have narrow target ranges, and it would be unsurprising to fail in identifying a target, given the thousands of insects against which it would be necessary to test. Nonetheless, the reviewers' points are correct. We believe that the Vpb4Aa2 protein that we present, has sufficient in common with the known active Vpb4Da2 and Vpb4Fa1 proteins to give constructive insights into how the Vpb4 clade of proteins may function. Our findings highlight similarities between these toxins at the functional pore-forming region that we identified as important components in the characterization of the Vpb4 clade (as detailed in the responses to reviewers below).

Finally, on the issue raised regarding terminology, the term electrophysiology has evolved to include not just cells and tissues but more general measurement of electrical properties in biological systems, which include artificial bilayers such as black lipid membranes or droplet interface bilayers (the one we used in the study). This has been addressed in section 1.1.

As a result, we maintain that our Vpb4Aa2 structure gives important insights into the potential function of this clade of proteins, and we demonstrate its ability to act as a bona fide pore through our electrophysiology studies.

We hope that these explanations will aid you in your decision to proceed with our manuscript submission.

Reviewer #1 (Remarks to the Author):

We want to start by thanking the reviewer in highlighting those areas of the manuscript that need to be clarified or strengthened. It is excellent feedback and we hope that we can adequately address each point below.

The manuscript by Wirawan et al. describes structural and some functional characterization of the bacterial toxin Vpb4Aa2 from *B. thuringiensis*. The protein can be classified based on its sequence and structure as a member of the bacterial exotoxin family highlighted by anthrax PA. However, well characterized bacterial toxins of the family are known to act as multiprotein complexes that include the cell binding protein translocase structurally homologous to Vpb4Aa2 and one or more "active" protein components, usually enzymes that when translocated into cytosol disrupt the cell in some way, leading to cell death. The target of the study presented in the manuscript is different in the sense that it is posited to provide significant toxicity without an active component, and authors suggest that the mechanism of toxicity is based simply on the disruption of the cell membrane and that cell death is driven by resulting electrolyte balance disruption.

1.1 The main focus of the manuscript is the structure determination of the membrane insertion form of the toxin by means of cryoelectron microscopy. As a structural biologist myself, I certainly appreciate this kind of analysis. Where I think the paper is lacking is the functional studies that could support some of the proposed conclusions, (limited here to some biophysical characterization of pore formation in a lipid membrane - electrophysiology is certainly not my area of expertise, but I am not sure if the included studies can be described as "electrophysiological" given that no cells or tissues were involved).

The reviewer is correct to note the origin of the electrophysiology technique implies measurements in living cells or tissues. However, it has come to include more general measurement of electrical properties of biological systems (and more recently biomimetic man-made nanopores) in isolated membranes, whether excised from cells or made artificially - such as those we employ here. In this context the term electrophysiology is in common usage to describe the electrical measurement of electrical activity / ion flux in membrane spanning pores and ion channels in artificial bilayers such as black lipid membranes, suspended lipid bilayers, droplet interface bilayers and patched GUVs. This includes the examples listed here: 10.1007/978-1-62703-351-0_8, 10.1007/978-1-0716-0818-0_4, 10.1016/bs.mie.2021.03.001, 10.1038/nprot.2015.003, 10.1038/nprot.2013.061, 10.1073/pnas.1609142114.

While it is true that we lack functional studies on the toxin target in vivo, our electrophysiological experiments do show in vitro functionality as a pore. We would also point out that our support for the function also arises from additional phylogenetic analysis (more on the next reviewer point 1.2).

1.2 The most important omission here is proof that the Vpb4Aa2 is, in fact, a toxin. Authors present some cell toxicity studies that, unfortunately, indicate that at least for the insect cell lines tested no toxicity is observed up to 0.1 mg/ml of protein concentration. Perhaps they are right and the cell lines they used lack the proper toxin receptor, but one cannot simply ignore the lack of the evidence that this specific protein is, indeed, insecticidal. Yes, the related protein Vpb4Da2 is reported to have such properties, but with midlevel sequence homology (57% identical) the function would have to be confirmed in some manner.

The reviewer is correct that despite testing against available insect cell lines, no target for the Vpb4Aa2 was discovered. While Vpb4Da2 has observed larvicidal activity against WCR, we were unable to express Vpb4Da2 recombinantly (10.1371/journal.pone.0260532). In addition, the Vpb4Fa1 protein sequence (known to kill *Monolepta hieroglyphica,* 10.3390/toxins17040167) is not publicly available and was not available upon request when the paper was initially published. However, we were able to express and purify Vpb4Aa2 and support its function through a combined phylogenetic analysis, structural analysis and electrophysiology approach to characterise the function of the Vpb4 proteins in general.

Insecticidal toxins tend to have narrow target ranges, and it would be unsurprising to fail to find a target given thousands of insects against which it would be necessary to test. What is important to understand is that the Vpb4 family has two members (Vpb4Da2 and Vpb4Fa1) that can each target two different species of beetle and kill them without the need for a translocated factor (10.1371/journal.pone.0260532, 10.3390/toxins17040167).

We have recently received the sequence of Vpb4Fa1, kindly provided by the authors of the paper (10.3390/toxins17040167), which we have incorporated into the manuscript. We maintain that the Vpb4Aa2 protein that we present, is sufficiently similar to Vpb4Da2 and Vpb4Fa1 (53% and 55.6% identity respectively; needle alignment) to give constructive insight into how the Vpb4 clade of proteins may function. Notably, Vpb4Da2 and Vpb4Fa1 (with known insect targets) share a 50% sequence identity with each other, comparable to the similarity of each of these to our Vpb4Aa2.

With respect to the functional pore forming domains (PFD, Domains 1-3) that are central to our discussion of the properties of the pore, we note that the Vpb4Aa2 identity with Vpb4Da2 and Vpb4Fa1 are even higher (68% and 77% respectively; 83.3% and 86.7% similarity). We, therefore, believe it is likely that Vpb4Aa2 would also have insecticidal properties against different targets. Based on our structural analysis of the Vpb4Aa2 pore, the features that we identify as important characteristics of this clade are located within this highly similar region (PFD, domains 1-3).

Furthermore, Kouadio et al. (10.1371/journal.pone.0260532) have also shown that a Vpb4 clade protein they named Vpb4C.6693 is non-toxic to Western corn rootworm (WCR) but that a chimera including the PFD (domains 1-3) from this protein with the receptor binding domains (RBD, D4-6) from Vpb4Da2, is WCR-active. This indicates that specificity is driven by the RBDs; and that the pore regions from otherwise non-toxic proteins are able to participate in active cell killing when appropriately targeted. The PFD of Vpb4C.6693 is 80.5% and 70.0% identical to the equivalent regions of Vpb4Da2 and Vpb4Aa2, respectively.

Revision to the Results of the manuscript:

Line 103: While we were unable to express Vpb4Da2 and Vpb4Fa1, we were able to express and purify the close homologue, Vpb4Aa2. Vpb4Aa2 is a Vpb4 member with overall identity of 53% and 55.6% to Vpb4Da2 and Vpb4Fa1 respectively.

Line 200: This further suggests that the β-barrel of the Vpb4Aa2 retains a net neutral charge, correlating with the fact that translocation is not required for cytolytic function.

Line 203: When comparing Vpb4Aa2 with the other members of the Vpb4 subclass, features of the pore lumen are conserved. Particularly, the ϕ-clamp landscape and the pore-forming loop were shown to be consistent in a sequence-level analysis (Supplementary Figure 6; and 8a and b), with the exception of Vpb4Ba1 as an outlier. Indeed, comparison to characterised family members (Vpb4Da2 and Vpb4Fa1) revealed increased identity in the functional PFD (domain 1-3), sharing 68% and 77% sequence identity, respectively (83.3% and 86.7%

similarity). In comparison, the receptor binding domains (domain 4-6) of Vpb4Aa2 only shares 42.9% and 35.7% identity to Vpb4Da2 and Vpb4Fa1 respectively. The identity is particularly reflected in the residues at the pore lumen of Vpb4Aa2 (coloured based on sequence conservation between the three proteins; Supplementary Figure 8c), suggesting similar mechanism with different targets. Thus, the structural features seen in the Vpb4Aa2 pore lumen are illustrative of other Vpb4 subclass members and provide valuable insights into how these proteins might function as simple lytic pore-forming proteins rather than as translocases.

Line 259: However, given the low identity on the receptor binding domains, it was unsurprising to fail to find a target given that insecticidal toxins tend to have narrow target ranges.

Line 336: … notably result in comparable changes in behaviour[44,45], along with an increase in channel conductance[45], reduction in cation selectivity[46], and abolition of translocation function[28].

Revision to the Discussion of the manuscript

Line 402: The Vpb4 subclass members (to which Vpb4Da2 and Vpb4Fa1 proteins belong) are encoded as isolated proteins and are functional in and of themselves, unlike the canonical members from the Bacterial Exotoxin B family[3]. To understand the mechanism of the Vpb4 proteins, we performed biochemical characterisations of a member of the Vpb4 subclass, Vpb4Aa2.

Line 407: While the protein specificity has yet to be determined against invertebrate targets, the identical fold of the N-terminal PFD (domains 1-3) between the protein and Vpb4Da2; and the high sequence similarity in this region to Vpb4Da2 and Vpb4Fa1 suggests analogous function in terms of pore-formation and oligomerisation.

Further explanation on the structural features of PA in the manuscript

Line 61: The charged clamps create an electrostatic filter within the pore serving as a charge-state ratchet. Together with the ϕ-clamp, the charged clamps prevent the return of the unfolded A component as it undergoes Brownian diffusion through the pore and deprotonation along the pH gradient.

Line 439: The other critical feature of Vpb4Aa2 is the presence of an alanine ϕ-clamp rather than the canonical phenylalanine critical for translocation. In PA, mutation from phenylalanine to alanine of the clamp abolished translocation, as the hydrophobic bottleneck composed of the phenylalanine is rationalised to be essential for preventing retro-translocation and producing directional bias[58].

Line 444: Changes in any of these regions are associated with failure in translocation, whilst retaining the ability to form pores with increased channel conductance and reduced ion selectivity[13,25-29]

Line 446: Here, we reason that this assemblage of distinct features in the Vpb4Aa2 pore, associated with loss of translocase function in archetypal family members, contribute to a different mechanism of action. This reconciles previously reported observations of the ability of Vpb4 sub-family members to kill at organism level without a partner toxin.

Minor addition of Vpb4Fa1 in the sentence:

Line 360: A phylogenetic tree was then constructed using these AFDB hits, together with the 6 members of the Vpb4 subclass taken from the Bacterial Pesticidal Protein Resource Center (BPPRC) at secondary first-letter name rank (Vpb4Aa1, Vpb4Aa2, Vpb4Ba1, Vpb4Ca1, Vpb4Da2, and Vpb4Fa1), …

Line 695: Initial phylogenetic analysis was performed on all Vpb4 subclass members from BPPRC[1,84] (Vpb4Aa1, Vpb4Aa2, Vpb4Ba1, Vpb4Ca1, Vpb4Da2, and Vpb4Fa1) ...

Line 708: Candidates for analysis consist of AFDB hits with Prob. > 0.8, TM-score > 0.6, and average pLDDT > 0.7 and each of the Vpb4 subclass members (Vpb4Aa1, Vpb4Aa2, Vpb1Ba1, Vpb1Ca1, Vpb4Da2, and Vpb4Fa1) …

Figure 2 and 7: *Addition of Vpb4Fa1 in the tree*

Supplementary Figure 6: *Addition of Vpb4Fa1 in the sequence level analysis.*

Supplementary Figure 8: *Addition of Vpb4Fa1 in the sequence level analysis and sequence conservation colouring of the pore. A cropped portion of the figure shown below.*

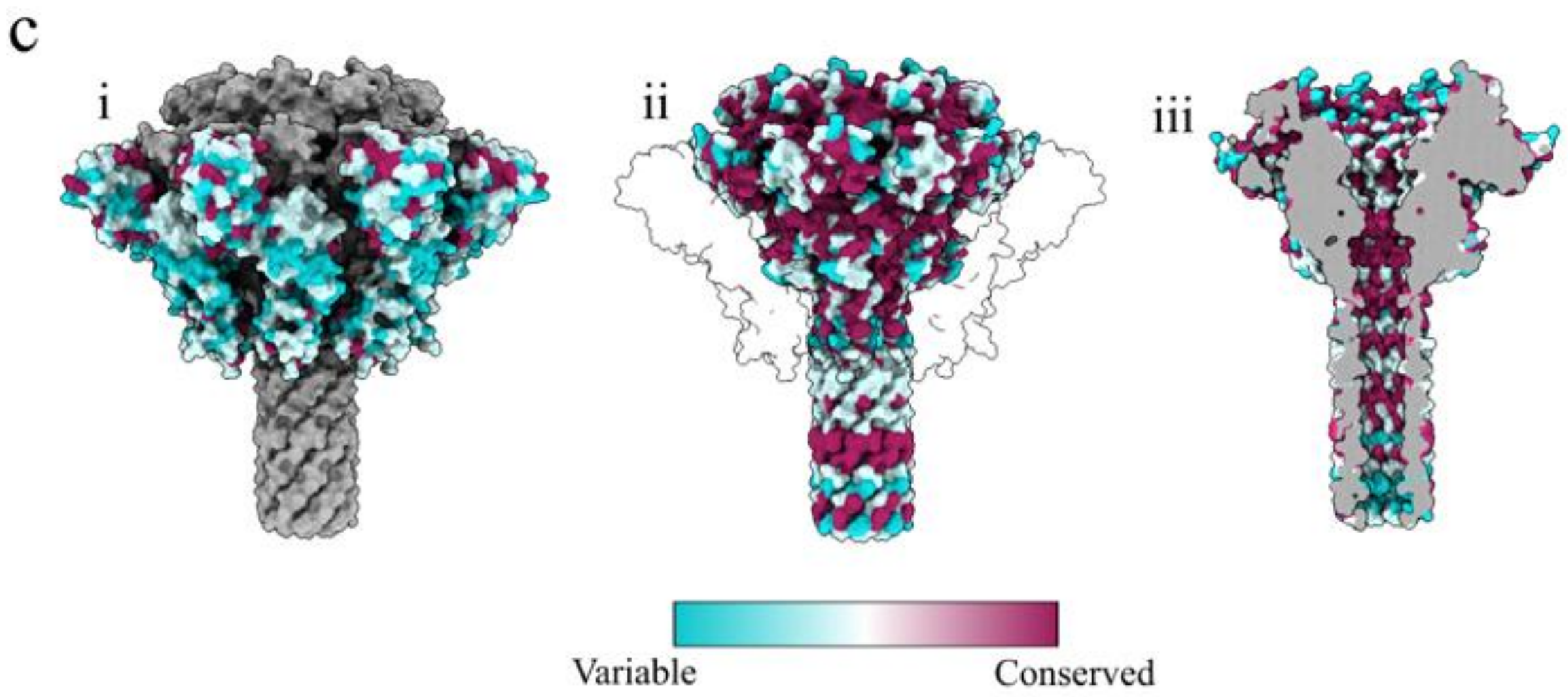

Supplementary Figure 12: *Addition of Vpb4Fa1 AlphaFold-predicted lumen*

Supplementary Table 5: *Addition of Vpb4Fa1 in the accession table*

Minor changes of Vpb4Da1/2 nomenclature to Vpb4Da2 for consistency to original literature (10.1371/journal.pone.0260532)

Line 70: The first documented example of toxicity exerted by this subclass is the Vpb4Da2 protein, …

Line 80: Current structural knowledge revealed that pro-Vpb4Da2 …

Line 136: Due to a lack of structural data on pro-Vpb4Aa2, we analysed the structural rearrangement of Vpb4Aa2 in comparison to the related protein pro-Vpb4Da2.

Line 221: … one extra domain, and Vpb4Aa2 and Vpb4Da2 having a further structural domain 6 (Figure 2a).

Line 232: The fold of domain 5 is also consistent to Vpb4Da2 and CdtB (Figure 4a) with an RMSD of 0.97 and 1.08 Å respectively.

Line 240: We next characterised the movement of the C-terminal domains of the Vpb4Aa2 pore with respect to the structure of pro-Vpb4Da2, which represents a related family member in its pro-form.

Line 253: Insecticidal activity has been demonstrated for Vpb4Da2 against Western corn rootworm and Vpb4Fa1 against the two-spotted leaf beetle[31,33]

Line 399: One of the emerging alternatives is the Vpb4Da2 protein, a Bt toxin discovered to be active against WCR with no cross-resistance with the currently deployed proteins (Cry3 and Gpp34/Tpp35)[30-32].

Figure 2, 4, and 7; Supplementary Figure 4, 5, 6, 8, and 12; Supplementary Table 5: *Changing Vpb4Da1/2 to Vpb4Da2.*

1.3 Furthermore, the assertion that this class of toxins lack the active component in vivo requires some proof as well. Authors should point to some studies that indicate that at least for the Vpb4Da2, at which point they could extend this as a hypothesis to the rest of the toxins that carry similar key structural elements. Note that just because Vpb4Da2 is insecticidal on its own does not prove that it is not binding some yet unknown toxic enzyme in vivo. I may be unaware of studies that provide such proof due to simple ignorance, so if I am mistaken please reference some specific papers that prove that Vpb4Da2 does not, in fact, have a partner enzyme. Sequence variation in the canyon region of the protein only indicates that it will not bind active components of other toxins, and does not prove that no such active component exists.

Thank you for highlighting this point. We seek to clarify here and in the manuscript what we mean by independent activity and how this differs to other clades in the Bacterial exotoxin B PFP family.

The Vpb4Da2 protein has been demonstrated to be active on its own (10.1371/journal.pone.0260532) as has the Vpb4Fa1 protein (10.3390/toxins17040167) in whole insect bioassays. This includes testing the protein expressed as a transgenic crop, and also purified recombinant protein administered through feeding assays. Although the reviewer is correct that activity alone does not preclude an interaction with a partner protein, we are uncertain how to demonstrate a lack of binding in this regard.

Circumstantially, we observe that within the two-component family of related toxins, the enzymatic A component tends to be encoded in close proximity to the B component, such as the Vpb1Ae1/Vpa2Ah1 (EF442245) and CdtA/CdtB (10.1371/journal.ppat.1005758), etc. Generally, the Vpb4 clade proteins have no identifiable enzymatic components encoded in the immediate vicinity (10.1016/j.ygeno.2019.06.020), particularly no ADP ribosyltransferases, adenylate cyclases or Zn metalloproteinases, which are the known enzymatic components for two-part toxins. We have also actively looked for evidence of a candidate partner within the proximity to the Vpb4 gene and do not find any associated genes. It is also important to point out that the well-conserved ϕ-clamp in the family has been replaced from phenylalanine to alanine in the Vpb4 family. It has been established (at least in PA) that F-to-A substitution results in loss of ability to translocate protein (10.1126/science.1113380).

It is impossible to rule out remotely encoded proteins of unidentified enzymatic character as translocation partners for the Vpb4 proteins but the ability to kill insects alone (demonstrated for Vpb4Da2 and Vpb4Fa1) indicates a significant effect worthy of structural study for this family. Should a cryptic partner protein be discovered in due course, our structure will be key in understanding any interaction and translocation, which must differ from the canonical mechanism of the wider family owing to the absence of the ϕ-clamp.

Revision to the Introduction of the manuscript:

Line 48: Examples of such enzymes include ADP ribosyltransferases (Iota-A, CdtA)[5,6], zinc metalloproteinases (LF)[7], or adenylate cyclases (EF)[8].

Line 68: Unlike archetypal family members, the Vpb4 subclass consists of proteins with no genes for enzymatic components (ADP ribosyltransferase, zinc metalloproteinase, or adenylate cyclase) encoded in the immediate vicinity of the Vpb4 gene[2].

Line 73: The toxicity has been shown through administration of purified protein in an artificial diet[31] and when expressed in genetically modified plant tissue[32]. Recently, another family member, Vpb4Fa1, was described to have activity in artificial diet assay against the corn pest Monolepta hieroglyphica (the two-spotted leaf beetle)[33]. To summarise, the Vpb4 subclass of proteins are unique as they are functional in killing whole organisms without needing to translocate a secondary component

Revision to the Results of the manuscript:

Line 105: Genomically, Vpb4Aa2 is located flanked by two transposases and without a secondary component encoded in its vicinity.

Line 168: In comparison to CdtA/CdtB and Iota-A/Iota-B binary toxin systems, Vpb4Aa2 shared the calcium binding region at Domain 1 important for binding of the A component. However, the NSS/NSQ residues in the loop previously characterised to clamp the enzymatic partners were not conserved in Vpb4Aa2 (Supplementary Figure 5).

Revision to Methods of the manuscript:

Line 518: To generate the coding sequence (CDS) map, the contig sequence containing the Vpb4Aa2 gene was annotated using Prodigal 2.6.3[64] to predict CDS of putative protein, followed by NCBI blastx[65] for protein prediction, and IGV[66] for gene visualisation.

Supplementary Figure 1: *Addition of contig map encoding Vpb4Aa2.*

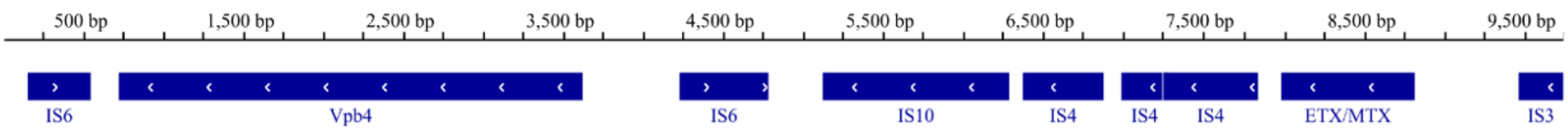

1.4 Perhaps one piece of supportive evidence could be to determine the toxicity of the Vpb4Da2 as the positive control in the cell toxicity assays utilized in the course of this study. It has been demonstrated that this toxin is lethal to western corn rootworm, so determining effective toxin concentration could be useful in order to establish a reference point.

We thank the reviewer for this suggestion; however, we are uncertain of the utility of the test. We know that Vpb4Da2 kills WCR: if it also kills the WCR cells, this only demonstrates that the Vpb4Da2 receptors are expressed in the cell line. The receptor binding domains, RBDs, are more divergent than pore domains within the family, with Vpb4Aa2 sharing only 42.9% identity (needle alignment to Vpb4Da2). We would not expect that the presence of Vpb4Da2 receptors in the cell line would be predictive of Vpb4Aa2 sensitivity.

We have included our bioassay data as we feel that these data are important to the field. It is difficult to publish results cataloguing insects (and cell lines) that have been tested without activity, but these data are central to understanding toxin specificity. As a result, we have

presented the data that we have to readers in the knowledge that the number of insects (and cells) tested is limited by availability and local quarantine regulations.

1.5 Authors also dismiss standalone toxicity of the other translocases as low level activity. At least for the CDTb toxicity is on the order of 0.5ug/ml. It would be useful to demonstrate that the Vpb4 class toxins have higher toxicity. Otherwise the conclusion perhaps should be that the Vpb4 is not a novel class of toxins that evolved to act without an enzymatic partner, but rather that it represents a simpler descendant of the ancestral translocase that has not yet evolved into the binary toxin.

We did not intend to dismiss the lower activity of CDTb alone and have tried to acknowledge it in the text. There are several publications involving these activities (10.1016/j.ygeno.2019.06.020, 10.3390/toxins13060390, 10.3390/toxins10060225) showing different levels of activity (from non-toxic to toxic) on different cell lines at the same concentration. Previously, PA has also been shown to cause cytotoxic activity against a macrophage cell line overexpressing the receptor (10.1111/j.1462-5822.2006.00708.x), as well as Iota-B from internalisation-compromised cell lines (10.1128/iai.05677-11). However, administration of purified protein has not been shown to result in lethality on animal models.

We have further investigated the independent activity of CDTb outside of cellular level. The recent study by Simpson et al. 2024 (10.1093/ofid/ofad040) has shown that CDTb-only strain resulted in increased virulence in hamsters (but not mouse model). Other than this, we are unaware of any publications where introduction of purified protein alone can kill whole organisms. In contrast, two purified Vpb4 proteins have been demonstrated to be larvicidal when fed in vivo. This is an important discussion point, and we thank the reviewer for pointing it out in the review. We have incorporated changes on activity by B components independent of A component in our manuscript.

Line 420: From a functional perspective, the most defining feature of Vpb4 is the ability to kill without having a secondary component. While cytotoxicity of canonical Bacterial Exotoxin B member in the absence of their cognate A component has been observed at a cellular level in CdtB[10,11], Iota-B[52], and PA[53], administration of purified protein has not been shown to result in lethality in animal models[54-56]. Interestingly, a recent study by Simpson et al. revealed that a *C. difficile* strain with the *cdtB* gene, but not the *cdtA* gene, increases the virulence level in comparison to strain without the two genes in hamsters model, but not in mice model[57]. Notably, administration of purified protein in this model has not been performed in hamsters model. Given that two different forms of Vpb4 have been shown to kill whole organisms as purified protein, we hypothesised that Vpb4 may function through different mechanisms of killing compared to PA, CdtB, and Iota-B.

We have also investigated another non-canonical family member, BECb, discovered in 2014 (0.1128/iai.01759-14) and recently re-visited in a 2025 study (10.1038/s42003-025-08519-5). In the 2025 study, it was reported that BECb has a serine ϕ-clamp that is strongly correlated to its function. Interestingly, BECb is also encoded with an ADP-ribosyltransferase component and is also directly involved in actin ribosylation (0.1128/iai.01759-14). Coincidentally, our database-search has captured BECb (A0A5K6V4V1) as a member with non-F ϕ-clamp with an AlphaFold-predicted charged pore lumen. Given that BECb is predicted to have charged lumen along the β-barrel, we argue that BECb shares functional property to both Vpb4 and canonical translocases. Points on BECb have been incorporated into the manuscript.

Line 379: In the broader tree topology, the Vpb4-like clade was observed to diverge early from the binary Vpb1-clade and non-Bacillus clade. Three other proteins from the genera Bacillus,

Clostridium, and Paenibacillus (A0A4U2N2X2, A0A5K6V4V1, and A0A1B2DME3) also exhibit the phenylalanine substitution of the ϕ-clamp, but each also contains negatively charged residues in its lumen (Supplementary Figure 12). Among them, A0A5K6V4V1 was designated to be BECb, a less studied non-canonical Bacterial Exotoxin B family member with a serine ϕ-clamp. BECb was shown to be functional as a translocase for the ADP-ribsoylating BECa protein in cell assay, but was also able to act independently to cause fluid accumulation in suckling mouse assay when administered independently[47,48]. In this regard, the lack of negatively charged residues and encoded enzymatic components to Vpb4 proteins could potentially define a structurally and mechanistically unique cluster of single-component, membrane-lytic toxins within the family.

Line 493: We also identified three family members that carry non-canonical ϕ-clamp and predicted negatively charged lumens. This includes the partially characterised BECb[47,48], which has a charged lumen resembling canonical translocases, but with a wider ϕ-clamp reminiscent of Vpb4 clade.

Given the number of proteins in our phylogenetic analysis, we were also unable to resolve whether the Vpb4 clade has evolved from a binary toxin or whether binary toxins evolved from Vpb4-like ancestors. We have made it clearer in the manuscript that we could not achieve this at our level of analysis

Line 499: Because our tree is constrained by a limited number of curated sequences, we are unable to resolve the nature of the last common Bacterial Exotoxin B ancestor (independent cytolytic versus binary translocating pores, or the intermediate).

1.6 It also appears to be the case that the authors use the argument of the allegedly low standalone toxicity of other translocases to bolster their hypothesis that Vpb4 class proteins insert directly into the cell membrane without the need to trigger the conformational change in mature endosomes. That may well be the case, but just like with some other conclusions offered, that would require more proof.

We thank the reviewer for the comment. We did not intend to draw associations between levels of standalone toxicity to the location where Vpb4 forms pore. At our level of analysis, we hypothesise that since we do not require endosomal level pH to trigger pore formation (based on the pore structure at pH 8 and establishment of the pore system at neutral pH in our electrophysiology work), Vpb4Aa2 may form pores without the need to travel to the mature endosomes. This contrasts with previous work (and our electrophysiology work in this study) with PA, where acidification drives pore formation and membrane perforation (10.1016/j.jmb.2024.168521).

Our intention is to demonstrate features of the pore form of a Vpb4 protein for comparison with pore structure of wider family members. It is notable that some of these features differ from archetypal family members, such as PA, which we believe is particularly noteworthy for reporting. We have not sought to use this as evidence for the location of pore formation, but rather discussed possible implications of these differences, especially when considered together, which provide significant lines for future hypothesis driven research. We have updated the manuscript to reflect this and streamlined our hypothesis to prevent over-interpretation.

Line 462: As we isolated the Vpb4Aa2 pore and demonstrate perforation of the membrane in DIB at neutral pH, we hypothesise that the protein may be able to insert into the membrane at this pH state in the physiological context. This is contrary to PA, where the low pH during

endocytosis drives pore formation[58]. Based on the calcium density that we observe at domain 4 in each subunit of our model, we also hypothesised that the pore formation of Vpb4 is not driven by a calcium depletion event in the endosome as previously reported in CdtB[12]. Given these observations, together with the loss of structural features required for translocation and lack of an encoded A component, we further hypothesised that pore formation for Vpb4Aa2 may occur at the cell surface, rather than requiring endocytosis and acidification. Whether endocytosis is involved during Vpb4Aa2 activity in vivo is currently unknown.

Main Figure 8: *Addition of maturation in endosome pathway*

1.7 Another possible approach would be to swap in the receptor binding domain from, say, CDTb and see if such a construct is toxic to Vero cells. This could bolster the argument that Vpb4's are effective toxins by simply piercing the outer cell membrane.

Yes, while we agree that a chimera would be a good idea to show activity, the proposed chimeric approach with CDTb is beyond the scope of our study due to the following reasons:

- The requirement of a specific "Dealings not involving intentional release" (DNIR) ethics approval to produce CDTb/chimeras in our institutions. CDTb is a known human pathogenicity factor that would require specific ethics approval that is currently not in place for our laboratory (this approval would take several months **prior** to commencing the work).
- The high-risk nature of an experiment in producing positive results. Even if the protein material could be successfully expressed, there is no guarantee that 1) the CDTb receptor binding portion will function as per the native protein and 2) that the Vpb4Aa2 pore-forming machinery will also fold and retain its function in such a chimera.

1.8 In essence, this is a very nice structural study augmented with some biophysical data on ion channel like properties. I do not have any concerns with that - the study itself is sound and makes some rather interesting observations regarding the beta-barrel geometry. I also find that this study provides an important contribution as it presents the first full length membrane inserted model structure of the anthrax-like bacterial translocase that has 3 receptor binding domains (the term which is rather unfortunate given that it is very likely that two of these do not bind any receptors). As a co-author of the recently published study on the role of endosomal calcium depletion in activation of the CDTb I find it particularly interesting if the authors could present some analysis on the CBM-like domain that appears to retain calcium (from what I understand, it's only detected in C1-refined structure, so it's possible that only some CBM-like domains retain the metal and not all) - perhaps to see whether calcium depletion may be effective in triggering the conformational change or at least to look into structural differences that may explain why calcium switch is not relevant for Vpb4Aa2.

We have re-analysed our cryo-EM map (see also point 2.3) and confirmed that the density for calcium ions is present in all subunits of the Vpb4Aa2 map both in C1 and C7 reconstruction (both our old and new map). In this regard, we anticipate that the removal of calcium is not required for pore formation to occur as per CDTb.

Revision to the Results of the manuscript:

Line 224: Our initial model consisted of domain 4 and 5 of the RBD. The fold of domain 4 in Vpb4Aa2 is consistent with Vpb4Da2, CdtB, and Iota-B; but not PA (Figure 4a). This includes the presence of calcium ions bound and the coordinating residues (Supplementary Figure 3a, Supplementary Figure 9a). As calcium depletion of D4 has been reported as a trigger for pore-

formation on CdtB[12], a C1 reconstruction of the pore model was initiated to confirm whether calcium was present on each subunit. The reconstruction at a global 2.3 Å resolution reveals density for calcium ions on each subunit, suggesting a different mechanism of pore-formation by Vpb4Aa2 (EMD-68793, Supplementary figure 9b).

Figure 4: *Analysis of domain 4 is now in this figure.*

Supplementary Figure 9: *Change to electron density map, which includes calcium density in D4 of the seven subunits of C1 reconstruction.*

Revision to the Discussion of the manuscript:

Line 465: Based on the calcium density that we observe at domain 4 in each subunit of our model, we also hypothesised that the pore formation of Vpb4 is not driven by a calcium depletion event in the endosome as previously reported in CdtB[12].

1.9 I am not sure if this is feasible, but it would be terrific if one could analyze the co-evolution of binding and active components of various toxins, if the goal is to provide supporting evidence that Vpb4 class of toxins evolved from binary toxins to a standalone hemolysin-like toxins.

We thank the reviewer for the suggestion. As previously mentioned regarding the evolution of related binary toxins (point 1.5), we have not and did not intend to determine the evolution of Vpb4 as the ancestor or the descendant. In this regard we do not take a position on whether Vpb4 evolved from a binary toxin to standalone or vice-versa. Our goal with the phylogenetic data was to demonstrate that the features we observe in the structural aspects of the study can reasonably inform other uncharacterised proteins with independent pore forming activity.

Reviewer #2 (Remarks to the Author):

We want to start by thanking the reviewer in highlighting those areas of the manuscript that need to be clarified or strengthened. It is excellent feedback and we hope that we can adequately address each point below.

In this manuscript, the authors determined a cryo-EM structure of Bacillus thuringiensis Vpb4Aa2, a recently discovered bacterial toxin, and performed electrophysiology experiments to show its unique characteristics compared to other toxins of the same family. The article is novel, based on well-performed experiments, and well-written.

I have only one major concern and a few minor comments before I can recommend the article for acceptance.

2.1 If I haven't missed anything, the authors couldn't show that the protein prep they are working on is toxic to any cells or organisms. While I can understand that this might be due to the high specificity to its receptor, that also can mean that the protein is non-functional due to the method of production or purification. If the latter is true, the following biochemical and structural analysis of such a sample becomes irrelevant. I find it necessary to show that the toxin is toxic at concentrations compared to those of other members of the family.

We acknowledge that we did not discover a target for our chosen Vpb4 family member. However, as detailed in the responses above to reviewer #1, we did demonstrate that it is able to form pores in artificial membranes. This demonstrates that the protein in the form in which we produced it, is able to elicit pore formation as described. Furthermore, we know that the salient features that distinguish Vpb4Aa2 from translocating members of the family, are conserved in the pore region when compared to the known toxic Vpb4Da2 and Vpb4Fa1 (Supplementary Figure 6-8). Thus, our Vpb4Aa2 structure allows us to develop a model for the mechanism of independent action of this class of proteins.

2.2 I thank the authors for sharing raw cryo-EM data and models. I find them of very good quality. Also great that you described methods and added supplementary figures about cryo-EM data collection and processing. I kindly ask to add scale bars to 2D class averages and show viewing direction distribution on Supplementary Figure 13. I kindly ask to add scale bars to 2D class averages and show viewing direction distribution on Supplementary Figure 13.

The requested information on the scale bars and viewing direction distribution has been added to the Supplementary Figure 13.

Requested information cropped from Supplementary Figure 13.

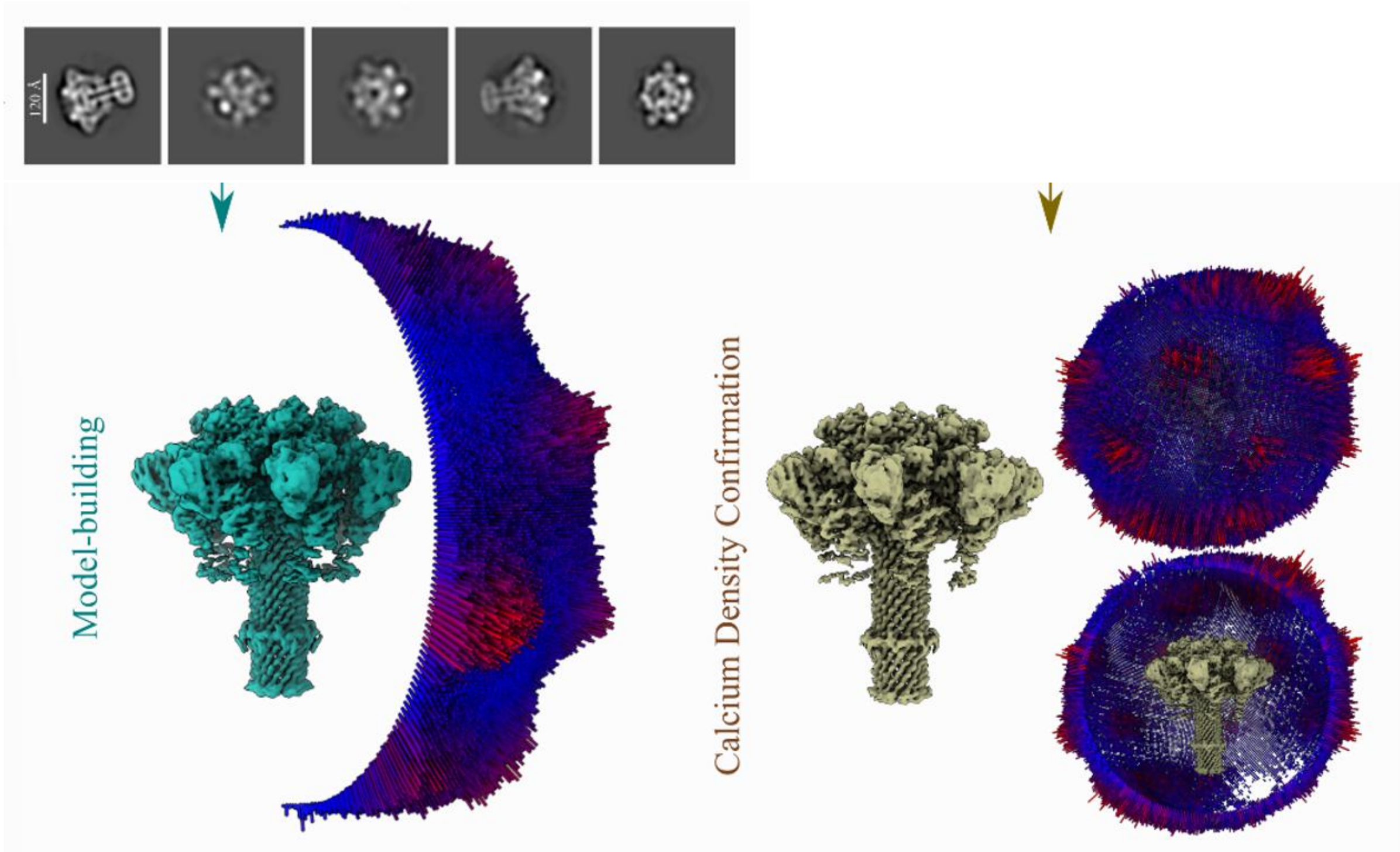

2.3 Finally, to the cryo-EM part, you show that some domains appear flexible and couldn't be resolved to high resolution. I recently had a similar experience but running refinement in Relion 5 with Blush regularization could improve it. I suggest trying it as well; it shouldn't take much time.

As per the reviewers' suggestion, we have re-analysed our refinement with Blush Regularization (and Bayesian Polishing) in Relion 5.0. Excitingly, we now have better resolved features and an improved global resolution in our map (from 3.12 Å to 2.2 Å global resolution). There is also slight improvement for domain 6. However, unfortunately it is still not enough for us to build the model of the domain confidently. In this regard, we have decided to proceed with a different model-building strategy, and the changes in the content have been incorporated in the manuscript (detailed below). We have updated our map and model for PDB/EMDB deposition standards, and we kindly thank the reviewer for helping us achieve better resolved features.

Updated results in the manuscript:

Line 120: We then performed single-particle cryo-EM analysis of the Vpb4Aa2 oligomers, revealing a heptameric pore structure at 2.2 Å with an imposed C7 symmetry in our reconstruction (Figure 1c). The structure shows higher localised resolution features within the core region of the pore, while the C-terminal extensions are comparatively poorer in resolution (Supplementary Figure 3). To gain a better understanding of the pore assembly interaction, an atomic model of the Vpb4Aa2 heptamer was determined, encompassing the well-resolved Domains 1-5 of the protein (~81% of the full-length residues: EMD-71647, PDB: 9PHF, Supplementary Table 1), while Domain 6 of the protein was too poorly resolved for model building. The dimensions of the Vpb4Aa2 pore are 155 Å and 175 Å height and width, respectively, with a 92 Å β-barrel and a 36 Å hydrophobic transmembrane region representing the proposed membrane-spanning portion of β-barrel (Figure 1c, Supplementary Figure 3d-f).

Line 224: Our initial model consisted of domain 4 and 5 of the RBD. The fold of domain 4 in Vpb4Aa2 is consistent with Vpb4Da2, CdtB, and Iota-B; but not PA (Figure 4a). This includes the presence of calcium ions bound and the coordinating residues (Supplementary Figure 3a,

Supplementary Figure 9a). As calcium depletion of D4 has been reported as a trigger for pore-formation on CdtB[12], a C1 reconstruction of the pore model was initiated to confirm whether calcium was present on each subunit. The reconstruction at a global 2.3 Å resolution reveals density for calcium ions on each subunit, suggesting a different mechanism of pore-formation by Vpb4Aa2 (EMD-68793, Supplementary figure 9b).

Main Figure 1: *New map in the figure.*

Main Figure 2*: New model in the figure.*

Main Figure 3*: New model in the figure. Figure now focuses on the pore-forming domain (domains 1-3). New Hole2 measurement.*

Main Figure 4: *New model in the figure. Figure now focuses on the receptor-binding domain (domains 4-6).*

Main Figure 5 and 6: *New model in the figure*.

Revision to the statistics and measurements of the model in the manuscript:

Line 19: Here, through single-particle cryo-electron microscopy, we elucidated the inserted pore structure of the Vpb4 subclass member, Vpb4Aa2, at 2.2 Å global resolution.

Line 146: The overall architecture and fold of the PFDs of Vpb4Aa2 remain consistent amongst the Bacterial Exotoxin B family (average RMSD = 1.188 Å).

Line 152: The average subunit-subunit buried surface area was estimated to be 3465.3 $Å^2$ with a ΔG value of -8.5 kcal/mol, consistent with other family members (Supplementary Table 3).

Line 176: The diameter analysis along the Vpb4Aa2 pore lumen revealed six clamp-like regions with luminal diameters between 9 and 21 Å (Figure 3a-c).

Line 191: The last two constrictions at the transmembrane region, composed of lysine residues (K356 and K341), tighten the lumen to ~11 Å and ~9 Å respectively.

Line 232: The fold of domain 5 is also consistent to Vpb4Da2 and CdtB (Figure 4a) with an RMSD of 0.97 and 1.08 Å respectively. Fold search using DALI revealed similarity to fungal immunomodulatory protein family members (FIP-Fve[36] and FIP-Nha[37]), sharing a carbohydrate-binding module (CBM)-34 fold (average RMSD = 0.96 Å)[38]. Domain 6 of the RBD, unfortunately, was still too poorly resolved for modelling. Instead, we made an additional model where AlphaFold was used to predict the domain fold and molecular docking was performed to highlight the possible rearrangement occurring during pore formation (Figure 4B, PDB: 22ZO, Supplementary Figure 9c).

Line 242: Here, domain 5 and 6 of the RBDs were observed to undergo a twisting motion, tightening towards the β-barrel (Supplementary Movie 1).

Supplementary Figure 3: *New map and model.*

Supplementary Figure 4: *New model to show coordination.*

Supplementary Figure 7: *New model and Hole2 calculation.*

Supplementary Figure 9: *EM map and local density.*

Supplementary Table 1-3: *Updated statistics and PDB-PISA interfaces.*

Updating methods for single particle analysis:

Line 583: Single particle cryo-EM analysis was performed using RELION (Version 5.0) through the M3 MASSIVE High-Performance Computing facility (Supplementary Figure 13). The raw movies were subjected to motion correction with MotionCor3 and Contrast Transfer Function (CTF) estimation with CTFFIND4[67]. Initial particle picking was performed using Laplacian of Gaussian picking at a maximum and minimum diameter of 325 Å and 175 Å on 347 micrographs. Particles were then extracted with a box size of 400 pixels and binned five-times prior to 2D classification to 50 2D classes. Classes with observable pore features were used for Topaz training and picking[68], followed by iterative 2D classifications. The final 2D classes were selected for 3D initial reference and 3D classifications to 5 3D classes. The final set of particles were re-extracted at 320 pixels prior to 3D auto-refine job with applied C1 or C7 symmetry. Particles then underwent CTF refinement[69] and Bayesian polishing[70] prior to the final round of 3D auto-refine. All refinement jobs were run using blush regularisation[71]. Masked refinement of the poorly resolved density was trialled, but no significant improvement was observed. The raw map is deposited to EMDB under the code EMD-71647 for the C7 reconstruction and code EMD-68793 for the C1 reconstruction.

Line 601: The pore model of Vpb4Aa2 was generated into the C7 symmetry map, encompassing domains 1-5 (N216-N853, ~81% complete chain).

Line 605: Due to limited densities, a loop in D5 (N828-D831) was omitted, while residues N346 and K347 at the tip of the β-barrel were replaced with alanine.

Line 607: The full-length subunit model of Vpb4Aa2 encompassed domains 1-6 (N216-S965, 94% complete chain), incorporating domains 1-5 from the C7 model and the auxiliary C-terminal domain 6. The C-terminal domain 6 was generated using ColabFold[72]. Due to poor resolution, domain 6 was docked (rather than built) into the density using fitmap command in ChimeraX 1.10.1[73], as highlighted in Supplementary Figure 9. Domain 6 was modelled as backbones with missing residues at S875-Y883, D921-R925, and G935-R937. The domain movement between pro-Vpb4Aa2 and full-length subunit of Vpb4Aa2 pore was visualised using the morph command in ChimeraX 1.6.1[73], as shown in Supplementary Movie 1.

Supplementary Figure 13: *Updated workflow.*

Supplementary Movie 1: *Updated map and model.*

Finally, two small textual points:

2.4 - Abstract, line 15: Bacterial exotoxin B is a vague name for me. It would also fit for streptococcal pyrogenic exotoxin B or TcdB of C. difficile. Is there an option to call it differently?

We agree that the name does sound as if it may encompass a wide range of two component toxins. However, the name “Bacterial exotoxin B” is derived from a pfam/interpro family name designation (IPR003896) so we have used this nomenclature for consistency with the field. We have ensured that we cite this family early to avoid any confusion with other AB type toxins.

As per Line 40: The Vpb proteins are members of the Bacterial Exotoxin B family (Interpro: IPR003896), being structurally related to the well-known …

2.5 - I would suggest using Iota-B instead of IB abbreviation for the C. perfringens toxin.

We have changed the nomenclature to Iota-B (and Iota-A) accordingly.

Line 40: The Vpb proteins are members of the Bacterial Exotoxin B family (Interpro: IPR003896), being structurally related to the well-known Protective Antigen (PA) of *Bacillus anthracis*, C2-II toxin of *Clostridium botulinum*, CdtB of *Clostridioides difficile*, Iota-B of *Clostridium perfringens* and other bacteria from the taxonomic phylum *Bacillota*[2,3].

Line 48: Examples of such enzymes include actin ribosylation (Iota-A, CdtA)[5,6], MAPKK cleavage (LF)[7], or increasing cyclic AMP concentrations (EF)[8].

Line 115: Indeed, further analysis by size exclusion and mass photometry (Figure 1b, Supplementary Figure 2) revealed that oligomerisation of Vpb4Aa2 could be achieved through ethanol treatment (as previously observed in Iota-B[21]), suggesting that the trypsin-activated material may be metastable and prone to spontaneous pore formation.

Line 141: The calcium-binding region at Domain 1 is conserved amongst the two Vpb4 subclasses, and other related toxins, PA, CdtB, and Iota-B (Supplementary Figure 4).

Line 145: We then analysed the structure of the Vpb4Aa2 pore with other pore structures of the Bacterial Exotoxin B family (PA, Iota-B, and CdtB) (Figure 2e).

Line 157: To date, the Bacterial Exotoxin B family members (PA, Iota-B, and CdtB) share common structural features along the pore lumen to allow translocation of a secondary proteinaceous toxin.

Line 168: In comparison to CdtA/CdtB and Iota-A/Iota-B binary toxin systems, Vpb4Aa2 shared the calcium binding region at Domain 1 important for binding of the A component.

Line 172: Thus, although Vpb4Aa2 conserves the Domain 1 calcium binding regions, the protein was hypothesised to be incapable of docking any secondary toxins of the current characterised systems (PA, CdtB, and Iota-B) due to the lack of α-clamp specific residues and the NSS/NSQ loop.

Line 179: Interestingly, the ϕ-clamp in Vpb4Aa2 is formed by a ring of alanine residues (A457), rather than the canonical 6 Å ring of phenylalanine residues as seen in PA, CdtB, and Iota-B…

Line 193: The Vpb4Aa2 pore does not contain the canonical negatively charged clamps along the pore lumen of PA, Iota-B, and CdtB (Figure 3c, Supplementary Figure 7).

Line 219: Other members of the family show variation in the number of additional domains at the C-terminus such as Iota-B and CdtB having one extra domain, …

Line 224: The fold of domain 4 in Vpb4Aa2 is consistent with Vpb4Da2, CdtB, and Iota-B; but not PA (Figure 4a).

Line 344: With the absence of ϕ-clamp, it is unlikely that Vpb4Aa2 could facilitate toxin translocation across the membrane, as characterised in other Bacterial Exotoxin B family members (PA, CdtB and Iota-B toxins).

Line 352: Searches of the PDB returned hits on the characterised family members CdtB, Iota-B, PA, and C2-II as expected. On the contrary, searches of the AFDB, on the contrary, revealed numerous uncharacterised members with varying degrees of sequence and fold similarity.

Line 363: …other structurally relevant family members (PA, CdtB, Iota-B, and C2-II), and Vpb1Ac1 as representative of the Vpb1 subclass (Figure 7b).

Line 366: Phylogenetic reconstruction of the Bacterial Exotoxin B family revealed a distinct clade of Vpb4-like proteins separate from the PA, CdtB, Iota-B, C2-II, and Vpb1Ac1 with high confidence as observed by the bootstrap value.

Line 563: Following the protocol for Iota-B oligomerisation[21], we treated Vpb4Aa2 with 10% v/v EtOH during the trypsinisation process (conditions as mentioned above).

Line 695: Initial phylogenetic analysis was performed on all Vpb4 subclass members from BPPRC[1,72] (Vpb4Aa1, Vpb4Aa2, Vpb4Ba1, Vpb4Ca1, and Vpb4Da2, and Vpb4Fa1), and other characterised Bacterial Exotoxin B members (PA, CdtB, Iota-B, C2-II, and Vpb1Ac1).

Line 708: Candidates for analysis consist of AFDB hits with Prob. > 0.8, TM-score > 0.6, and average pLDDT > 0.7 and each of the Vpb4 subclass members (Vpb4Aa1, Vpb4Aa2, Vpb1Ba1, Vpb1Ca1, Vpb4Da1/2) and characterised Bacterial Exotoxin B members (PA, CdtB, Iota-B, C2-II, and Vpb1Ac1).

Figure 2 and 7; Supplementary Figure 4-6, 8 and 12: *Changes from IB to Iota-B.*

Other changes:

Changes in authorship order.

Raymond Wirawan[1], David Jamieson[2], Hannah Baird[2], Colin Berry[3], Chris J. Lupton[1], Hari Venugopal[5], Luis E. Valentin-Alvarado[1], Hannah L. Best[3], D. Dafydd Jones[3], Lainey J. Williamson[3], Husam Sabah Auhim[3,4], **Oliver Castell[2]**, **Michelle Dunstone[1]**, **Bradley Spicer[1]**

Changes to Author Information:

These authors contributed equally: Raymond Wirawan, David Jamieson, Hannah Baird

These authors contributed equally: Oliver Castell, Michelle Dunstone, Bradley Spicer

Correspondence to Raymond Wirawan, Oliver Castell, Michelle Dunstone, or Bradley Spicer

Changes to Acknowledgements statement:

Line 732: The authors thank Prof Primitivo Caballero, Universidad Publica de Navarra, Spain for kindly providing the contig to B. thuringiensis strain V004.17. The authors thank Dr E. Diane Williamson, Dstl, Salisbury, UK for kindly providing PA protein to the Castell Laboratory. The authors thank Dr C. Bayly-Jones and Reviewer #2 for the suggestion for cryo-EM data processing. The authors thank A/ Prof Bryan Krantz for thoughtful discussion on anthrax PA.

Changes in percent identity and similarity to reflect EMBOSS needle pairwise alignment when comparing protein one-to-one.

Line 104: Vpb4Aa2 is a Vpb4 member with overall identity of 53% and 55.6% to Vpb4Da2 and Vpb4Fa1 respectively.

Line 137: …we analysed the structural rearrangement of Vpb4Aa2 in comparison to the related protein pro-Vpb4Da2 (53% overall sequence identity, same domain composition).

Line 206: Indeed, comparison to characterised family members (Vpb4Da2 and Vpb4Fa1) revealed increased identity in the functional pore-forming domains (domain 1-3), sharing 68% and 77% sequence identity, respectively (83.3% and 86.7% similarity). In comparison, the receptor binding domains (domain 4-6) of Vpb4Aa2 only shares 42.9% and 35.7% identity to Vpb4Da2 and Vpb4Fa1 respectively.

Line 603: The β-barrel pore was modelled using the pre-existing cryo-EM model of CdtB (PDB: 7YVS, 35% sequence identity), while the remaining portion of the model was generated using ColabFold.[72]

Line 622: One-to-one sequence identity and similarity was calculated on EMBOSS Needle as part of EMBL-EBI Job Dispatcher[79]. Multiple sequence alignment and group sequence identity calculation were performed on Clustal Omega as part of EMBL-EBI Job Dispatcher[79].

Additional sequences on Supplementary Table 5.

Supplementary Table 5*: Vpb4Fa1, FIP-fve, and FIP-nha; correction for codes on Vpb4Da2.*

Increased awareness on Vpb4Fa1 target.

Line 395: The two-spotted leaf beetle (M. hieroglyphica) is another important pest targeting broad host crops in East and Southeast Asia, particularly in China[50]. Given the rising resistance against currently deployed insecticides[51], there is a pressing need to develop novel pesticides (including pesticidal pore forming proteins) with distinct modes of action as an additional tool for pest management.

Line 401: … and Vpb4Fa1, a Bt toxin recently discovered active against *M. hieroglyphica.*

Changes to trimming for phylogeny:

Line 700: Gaps were then trimmed using trimAL[86] in automated mode.

Line 713: Phylogenetic analysis was performed as described for characterised family members, except trimming was performed using a gap threshold of 10% instead to prevent over-trimming.

Minimise the use of the term "novel":

Line 28: Through our findings, structure-guided database search allows identification of ~~novel~~ putative Vpb4-like proteins, which suggest a broader class of single-component proteins within the larger family.

Line 368: This Vpb4-like clade comprises all previously recognised Vpb4 subclass members, seven newly identified *Bacillus* proteins, and a single ~~novel~~ protein from *Brevibacterium* (Figure 7b).

Line 399: One of the emerging alternatives is the Vpb4Da2 protein, a ~~novel~~ Bt toxin discovered to be active against WCR with no cross-resistance with the currently deployed proteins (Cry3 and Gpp34/Tpp35)[30-32]

Line 488: Using heuristic FoldSeek[63] query searches of Vpb4Aa2 pore-forming domains in the AFDB, we were able to define a Vpb4-like clade containing all currently recorded Vpb4 subclass members and ~~novel~~ Vpb4-like candidates ~~proteins~~.

Other minor changes:

Line 51: Upon oligomerisation, the complex is then able to perforate the membrane. Several triggers for pore formation have been reported, such as acidification within the endosomal environment (PA)[9], receptor binding (CdtB)[10,11] or calcium depletion (CdtB).[12]

Line 85: Given the roles of these more divergent domains in target specificity, the difference does not explain the ability of the protein to function in a single-component fashion.

Line 89: In this study, we elucidate the structure of Vpb4Aa2, a member of the Vpb4 family, in its pore form.

Line 92: This renders the Vpb4Aa2 pores distinct from PA, instead displaying characteristics of typical cytolytic pores.

Line 108: To isolate the complex of Vpb4Aa2, we produced trypsin-activated Vpb4Aa2 in the presence of LMNG/CHS detergents.

Line 139: The majority of pore-forming domains (PFDs, Domains 1-3) remain predominantly unchanged when comparing the monomeric and pore forms of Vpb4 (Figure 2c-d).

Line 269: In this case, an aqueous droplet is brought into contact with a spin-coated hydrogel layer on a glass coverslip, in an oil environment with dissolved lipid, creating a lipid bilayer …

Line 275: Figure 5 shows the fluorescence signal by Ca^{2+} flux through three individual Vpb4Aa2 pores (Figure 5c and Supplementary Movie 2).

Line 356: To predict proteins sharing the same potential mode of action as Vpb4 proteins, we performed phylogenetic analysis of AFDB hits …

Line 370: Interestingly, sequence-level analysis of the members of the Vpb4 clade showed that 13 members had a consistent lack of the canonical phenylalanine ϕ-clamp with either missing …

Line 411: Akin to the rest of the Bacterial Exotoxin B family members, Vpb4Aa2 undergoes proteolytic removal (at K211 according to Edman Degradation) of the ~20 kDa pro-domain to form functional pores[3].

Line 431: To further understand this distinct mechanism, we looked into whether the Vpb4Aa2 pores exhibit key structural features for translocation.

Line 484: Whether pre-pore formation occurs during the pore formation pathway remains an open question.

REVIEWERS' COMMENTS

Reviewer #1 (Remarks to the Author):

Thank you again for diligently identifying points of concern that need to be addressed to avoid any incorrect interpretation of the Bacterial_Exotoxin_B family. We have tried to address these comments, and we hope that this is a better manuscript based on this feedback.

1.1 The main concern of my initial review was that the manuscript lacked experimental evidence that the protein authors selected for their structural study is indeed insecticidal (both as capability and true function). The revised version of the manuscript does not address this concern and instead argues in a clearer manner that because the target protein has ~50% identity with another B.thuringiensis protein that was shown to have some level of insecticidal activity it therefore must be an insecticide. I checked and one of the classic binary toxins of the same heptameric translocase class, CDTb, shares about 43% sequence identity with the Vpb4Da2 – but obviously one would not assume that CDTb translocase is a non-binary toxin just based on sequence identity (and obviously is not an insecticidal toxin). Especially in bacteria with its powerful horizontal gene transfer mechanisms, functional conclusions based on sequence alignments are always difficult (and yet admittedly leave a lot of room for various hypotheses, which is obviously exciting).

We appreciate that the reviewer shares our interest in the possible hypotheses this study has stimulated, and we have two exciting follow-up collaborative studies on the Vpb4 proteins based around the sequence alignment/phylogeny work and application in beetle pests.

In regard to the sequence identity and assumption of a toxin as non-binary, it is important to note that this is exactly why we performed a robust phylogenetic study rather than relying on only sequence identity percentages, as this is the most objective way to look at protein similarity. Nevertheless, we have compiled the % sequence identity (and similarity) below, both for full-length and only the pore-forming domains (D1-3). We emphasize here, we do not assume that a protein is a non-binary toxin just based on sequence identity. We also take into consideration the lack of structural features needed for a binary toxin to function. These are aspects that were raised from our pore structure, and we are excited to push this out for other researchers in the Bacterial_Exotoxin_B field.

Table 1. Full-length sequence identity and similarity between the three Vpb4 members vs. CdtB

% identity (% similarity)	Vpb4Aa2	Vpb4Da2	Vpb4Fa1	CdtB
Vpb4Aa2		53.0% (67.4%)	55.6% (70.9%)	35.4% (52.2%)
Vpb4Da2	53.0% (67.4%)		49.9% (64.9%)	**28% (43.3%)**
Vpb4Fa1	55.6% (70.9%)	49.9% (64.9%)		32% (47.2%)

CdtB	35.4% (52.2%)	**28% (43%)**	32% (47.2%)	

Table 2. Pore-forming domain (D1-D3) sequence identity and similarity between the three Vpb4 members vs. CdtB

% identity (% similarity)	Vpb4Aa2	Vpb4Da2	Vpb4Fa1	CdtB
Vpb4Aa2		69.4% (83.9%)	75.6% (86.4%)	48.8% (65.3%)
Vpb4Da2	69.4% (83.9%)		68.7% (82.3%)	45.7% (64.1%)
Vpb4Fa1	75.6% (86.4%)	68.7% (82.3%)		48.5% (63.6%)
CdtB	48.8% (65.3%)	45.7% (64.1%)	48.5% (63.6%)	

To avoid confusion about percentages, we refer the reader back to the phylogenetic relationships in the discussion through these changes:
Line 396: While the protein specificity has yet to be determined against any targets (invertebrate or otherwise), the identical fold of the N-terminal PFD (domains 1-3) between the protein and Vpb4Da2, combined with the very close phylogenetic relationship with Vpb4Da2 and Vpb4Fa1, suggests analogous function in terms of pore-formation and oligomerization.

In regard to the insecticidal function of Vpb4 proteins, the ability of Vpb4 proteins to kill whole organisms has been demonstrated (as cited in the text and previous rebuttal). For Vpb4Aa2, we agree that as we do not have sufficient evidence that the protein is indeed insecticidal, we have removed comments on Vpb4Aa2 status as insecticidal and put more emphasis on characterized members (Vpb4Da2 and Vpb4Fa1) in the introduction and results section.

Introduction:
Line 78: To summarise, two members of the Vpb4 subclass of proteins are unique as they are functional in killing whole organisms without needing to translocate a secondary component.
Line 81: There is limited structural information to explain how these Vpb4 proteins function as isolated pores. A current structure reveals that a member of the Vpb4 subclass, pro-Vpb4Da2, adopts …
Line 88: Thus, there is a knowledge gap in understanding how some of the Vpb4 proteins oligomerise and function as members of the Bacterial_Exotoxin_B family without needing to deliver a secondary component.
Line 95: These results suggest a molecular mechanism that is unique within the family, consistent with the capability of some members of the Vpb4 subclass to act alone without needing to translocate a secondary factor.

Results:

Line 104: Here, we aimed to address how Vpb4 proteins might act as independent pore-forming toxins.

Line 163: Given that two members of the Vpb4 subclass function as independent cytolytic pores, …

Line 201 (*removed*): ~~This further suggests that the b-barrel of the Vpb4Aa2 retains a net neutral charge, correlating with the fact that translocation of a toxin component is not required for insecticidal activity.~~

Line 205: Indeed, comparison to the two characterised active family members, (Vpb4Da2 and Vpb4Fa1,) revealed …

Line 212 (*removed*): ~~Thus, the structural features seen in the Vpb4Aa2 pore lumen are illustrative of other the Vpb4 subclass. members and provide valuable insights into how these proteins might function as simple lytic pore-forming proteins rather than as translocases.~~

Line 254 (*partially removed*): However, no significant effects of Vpb4Aa2 were seen with these insect cells~~, which may reflect that the source insects are refractory or that the required receptors are not present in these cell lines. However, given the low identity on the receptor binding domains, it was unsurprising to fail to find a target given that insecticidal toxins tend to have narrow target ranges. To further probe for activity, we assessed the capacity of the protein to form pores in artificial membranes~~.

Line 347 (*partially removed*): To characterise the ~~proteins sharing the same potential mode of action as~~ Vpb4 subclass further, we performed phylogenetic analysis, …

Line 365 (*partially removed*): Notably, two uncharacterised proteins (A0A193D1L2 and A0A2C9ZD13) in the Vpb4 clade also originate from *B. thuringiensis* strains, and thus are putative Vpb4 members ~~as potential insecticidal agent candidates due to sharing the structural features of Vpb4 and originating from *B. thuringiensis* strains.~~

Line 377 *(partially removed)*: In this regard, the presence of complementary charged residues in the lumen and missing encoded enzymatic components to Vpb4 proteins could potentially define the Vpb4 subclass~~ of a structurally and mechanistically unique cluster of single-component, membrane-lytic toxins within the family.~~

General comments on Vpb4Aa2 as insecticidal will be limited to the discussion section.

Line 396: While the protein specificity has yet to be determined against any targets (invertebrate or otherwise), the identical fold of the N-terminal PFD (domains 1-3) between the protein and Vpb4Da2, combined with the very close phylogenetic relationship with Vpb4Da2 and Vpb4Fa1, suggests analogous function in terms of pore-formation and oligomerisation.

Line 409: From a functional perspective, the most defining feature of the two members of Vpb4 subclass (Vpb4Fa1 and Vpb4Da2) is the ability to kill without having a secondary component.

Line 415: …without the two genes in a hamster model, but not in a mouse model. Notably, administration of purified CdtB protein was not performed in the hamster model. Given that two different forms of Vpb4 have been shown to kill whole organisms as purified proteins, we hypothesised that the Vpb4 subclass may function …

Line 439: Thus, the Vpb4Aa2 pore lumen is illustrative of other Vpb4 members and provides insights into how these proteins might function as simple lytic pore-forming proteins rather than translocases.

Line 442: The Vpb4Aa2 cell-based assays performed against WCR cells did not produce evidence of activity, which may reflect that the source insects are refractory or that the required receptors are not present in these cell lines. Given the low identity of the receptor binding domains, this is not surprising, given that insecticidal toxins tend to have narrow target ranges. To further our understanding of the Vpb4Aa2 function as a pore, electrophysiology experiments were undertaken. Electrophysiology experiments in our electrophysiology study …
Line 456: Here, we hypothesise that both the loss of the bottleneck and the charge profile of the pore lumen are critical in the independent function of the Vpb4Aa2 subclass.
Line 471: … we propose that at high concentration, each Vpb4 subunit is more likely to interact with each other and oligomerise.
Line 499: Here, we propose that the two identified Vpb4-like proteins originated from *B. thuringiensis*, A0A193D1L2 and A0A2C9ZD13, may exhibit insecticidal activity, are members of the Vpb4 subclass and may exhibit activity against invertebrates in a similar manner to Vpb4Fa1 and Vpb4Da2.
Line 503: Collectively, our study explains the ability of members of the Vpb4 subclass to cause membrane damage directly …

1.2 As I said previously, authors present excellent structural and biophysical data. Yes, the protein forms a channel (not surprising given its homology to bacterial protein translocases). Yes, related proteins appear toxic to insects. What is a leap for me is the proposal that this means that this protein has evolved to be an insect toxin without the need for an enzyme payload. I understand that this could be my reading of what the authors propose, and perhaps I have gone too far.

Indeed, it might feel like a leap that the protein has evolved to function without a secondary factor when sharing similar protein folds to binary toxins. However, to be objective with the current available data, it would also be a leap to conclude the opposite, (i.e., that the only way this protein functions is via a secondary factor) given the combined data listed:

- no genome associated factor,
- lack of the ϕ-clamp,
- overall neutral lumen
- different electrophysiological properties
- close phylogenetic relationship to Vpb4Da2 and Vpb4Fa1 (demonstrated to be active alone in their ability to kill whole organisms)

It may also be interesting to note that bacterial toxins (and insecticidal toxins in particular) can show a lot of flexibility regarding their use of partner proteins to exert activity. For example, in the α-helical App family of insecticidal toxins, there are examples of proteins causing insect toxicity in an independent manner (e.g., App6Aa2[1]) and others in a two-component manner (e.g., App1Da1/App2Ca1 also known as Plu1961/Plu1962[2]).These proteins are all related to the tripartite enterotoxins Hbl and Nhe from *Bacillus cereus*[3] and this structural group of related proteins can act through one, two or three component systems. Other examples include the Tpp class where there are single component toxins (Tpp80[4]), two components with same class member (Tpp1/Tpp2 also known as BinAB[5]), and two components with different class members

(Tpp35/Gpp34 also known as Cry35/Cry34[6]; and Tpp49/Cry48[7]). Thus, protein family members evolving to act with/without partners certainly has precedents in the field.

1.3 But if that is indeed the suggestion, then in my mind it would require a bit more evidence. I still see two problems here. Firstly, the protein itself does not show cell toxicity. I understand that the authors believe that this is simply because they didn't stumble across proper target yet. I understand the assumption that all the proteins of this class feature interchangeable receptor binding domains which allows them to switch targets while maintaining core architecture and ability to perforate membranes. Still, what authors show is that the protein can form pores spontaneously without the need for environmental changes or presence of receptor. So why then it is not just toxic across the board to all insects? Isn't it also plausible that it has evolved a different function? In the end it comes down to the fact that structure is there to explain function and cannot be used as proof by itself.

We agree that structure informs function but is not proof of function. The point about general toxicity is a great point and one that people working across many different pore-forming protein (PFP) families might take for granted.

Regarding spontaneous pore formation, it is quite common for some PFPs to spontaneously form pores due to high protein concentration or elevated physio-chemical parameters utilised in single-molecule biophysical experiments. Possibly the most relevant example to this study is PA63 forming pores in solution[8] and on membrane[9] without the presence of its cognate receptors, ANTXR1/TEM8 or ANTXR2/CMG2[10]. CdtB has also been found to form pores or di-heptamers in solution without receptors[11,12]. However, one could not argue that these proteins are toxic across the board to all cells as some cell lines have also been demonstrated not susceptible against these proteins[13,14].

We agree that the structure cannot be used as a proof that the protein is insecticidal through assays, and we have made changes in the manuscript associated with this (as described in 1.1).

1.4 Secondly - perhaps I misunderstand the entire point here, and the focus is on how these proteins might function when used as agricultural tools. Let me explain. When I think of bacterial protein toxins I assume that they are pathogenic to host at concentrations produced by bacteria upon normal colonization. For example, CDT kills humans not only because the translocase component perforates mature endosomes, but because the enzyme destroys cytoskeleton. CDTb by itself is toxic to Vero cells, but in concentrations that are much higher and not practical for the bacteria to produce (but could be useful if one chooses such scaffold as a poison protein). Is it possible that Vpb4 class protein toxicity is not inherent to its evolved function? I understand that Vpb4Da2 was shown to kill WCR, but it's not obvious how effective protein concentration in those experiments compares to in vivo values and background levels of other translocase toxicity.

It is true that we do not know exactly whether *B. thuringiensis* (Bt) produced Vpb4 in a level sufficient to kill an insect in vivo, or the true motive behind the production of Vpb4 toxin by Bt. However, we know that transgenic plants expressing Vpb4Da2 have been demonstrated to be

effective against WCR to both susceptible wild-type colonies, and, importantly, colonies resistant against current available treatment[15]. Therefore, the proof-of-concept experiments have already been performed for at least one member of the Vpb4 subclass, with potential as agricultural tools.

1.5 I also expect that binary toxins evolved from some channel forming protein by acquiring ability to bind enzyme payload, not the other way around. It is fairly obvious that binary toxin system is much more efficient and it's unclear why organism would evolve towards lower toxicity (well, on the other hand, killing the host is not always the bests option). Thus the argument could be that Vpb4 class of proteins might serve as a model of ancestral protein that might have had some limited toxicity, perhaps in addition to its true function. It's an interesting proposition, and given reasonable homology levels across all classes of these translocases, it might be just as likely as reverse evolution.

We agree with the reviewer's perspective on both scenarios of evolution. As we previously mentioned in both our rebuttal and manuscript, we were trying to present both scenarios and did not decide on whether the binary or the independent toxins are the ancestor. We simply reported that the Vpb4 subclass members and other uncharacterised Vpb4-like proteins cluster together, and this can be used as a practical filter to explore more independent family members. The evolutionary direction of the translocating and independent clades in the family would benefit from a separate study and is currently under way.

1.6 Also, and correct me here if I am wrong, but B.thuringiensis uses cry proteins as the major toxin against its insect hosts. Unless yet unknown insect target is resistant to cry proteins, it's unclear what would be the evolutionary pressure for evolving new hemolysin style toxins.

This is incorrect but raises an interesting point about the perception of Bt toxins, which we thank the reviewer for raising. With respect to Bt, there are literally hundreds of different characterised Bt strains that contain a mixture of PFPs from different structural families, which is the result of a relatively high level of gene plasticity. This allows Bt to target many different insects and survive in different ecological niches. Most individual Bt strains produce multiple toxins (either multiple Cry toxins or a range of toxins from different classes). It is generally speculated in the field that this is to provide sufficient redundancy to help to avoid an insect easily evolving resistance. For example, *Bt israelensis* strains may produce 5 (or more) individual toxins (in Cry and Cyt classes) active against mosquito targets[16].

It is also possible that the reviewer is referring to Cry toxins under naming systems assigned prior to the 2021 revision of the nomenclature[17]. In the old system, proteins produced as crystals by Bt (and other organisms) during sporulation are simply referred to as Cry proteins. After the nomenclature revision, those proteins were reclassified into 5 characterised and distinct structural classes: App, Gpp, Mpp, Tpp, and Cry (a name that now only applies to a single structural class). Some previously named Cry toxins have also been reclassified into the Xpp class of toxins with uncharacterised structure, and the class is predicted to encompass at least 4 unique structural families.

In reality, while Bt strains are known to produce crystal proteins during sporulation, they also have a further arsenal of toxins present in its vegetative state. Protein classes expressed in the vegetative states also varies widely in structure and represent 4 further structural classes: Vpb/Vpa classes (which include our protein of interest Vpb4 and the Vpb1/Vpa2 binary toxin pair), Vip3 class and also a protein from Mpp class. Specific strains may also produce Hbl and Nhe tripartite toxins (well-known from Bacillus cereus) and a non-protein nucleotide analogue beta exotoxin. Therefore, production of a wide range of toxins in a variety of different structural groups is well established and is the norm for Bt. Thus, the presence of a Vpb toxin in a strain that may produce other active toxins is not in the least surprising. The evolution of an arsenal of toxins may broaden the target range of the host bacteria and are likely to be important in creating a barrier for the evolution of resistance in targets.

1.7 I am also surprised that authors could not express the Vpb4Da2. The monomeric form of that protein was determined by crystallography, which often indicates that the protein does not present significant complications when it comes to expression. Could you at least swap in Vpb4Da2 receptor binding domains and test the chimeric protein on WCR?

Yes, we are surprised as well and wish this was not the case. Nevertheless, the chimera is a great idea and something that we are considering pursuing depending on grant funding and time. A potential problem is that a negative result for pore formation (i.e. we can't induce it to form pores) might be due to either not having the right chemical conditions or the effect of the change in receptor binding regions on pore formation particularly as they are predicted to interact with the lumen (Figure 4).

1.8 In summary, the impression one gets from a superficial look at the manuscript is that it discovers example of binary toxin evolution towards non-binary membrane perforating version. I suspect that the reality might be much more complex (but also unfortunately more mundane) than this exciting proposition. It would be great if the author's proposition is correct, but it might require more experimental evidence (starting with demonstrating that the protein they chose is indeed toxic to insects).

Just by way of correction, we cannot currently determine the order of evolution of the "non-binary" function (as mentioned in 1.5). Instead, we are putting together all of the key data that characterises this unique and different Vpb4 subclass (clade). This is the first study describing the properties and will undoubtedly be useful for anyone who encounters not only Vpb4 proteins, but any other novel Bacterial_Exotoxin_B family members in the future.

Changes in result section associated with this comment:
Line 368: In the broader tree topology, the Vpb4-like clade was observed to diverge early from a common ancestor with the binary Vpb1-clade and non-Bacillus clade.

Reviewer #2 (Remarks to the Author):

The authors addressed my concerns and majorly improved cryo-EM reconstructions. I recommend the article for publication.

Thank you for the suggestions. We are glad that we have satisfied your concerns.

Other changes:
Author name and formatting changes.

Changing Abstract to be under word limit.

Changing Bacterial Exotoxin B to Bacterial_Exotoxin_B

Italicising gene name
Line 71: …immediate vicinity of the *vpb4* gene.
Line 108: Genomically, the *vpb4Aa2* gene is located flanked by two transposases and without a secondary component encoded in its vicinity (Supplementary Figure 1a).
Line 514: The *vpb4Aa2* gene sequence (accession MZ766551) was amplified from *Bacillus thuringiensis* strain V004.17. To generate the coding sequence (CDS) map, the contig sequence containing the *vpb4Aa2* gene was annotated using…
Supplementary Figure 1

Minimising exaggerating words
Line 71: The earliest documented example of toxicity…
Line 97: Through FoldSeek heuristic search and phylogenetic analysis, we highlight a clade of uncharacterised proteins with the distinguishing features of the Vpb4 subclass.
Line 358: This Vpb4-like clade comprises all previously recognised Vpb4 subclass members, seven uncharacterised *Bacillus* proteins, and a single protein from *Brevibacterium* (Figure 7b).
Line 365: Notably, two uncharacterised proteins (A0A193D1L2 and A0A2C9ZD13) in the Vpb4 clade also originate from *B. thuringiensis* strains and thus are putative Vpb4 members.
Line 386: …there is a pressing need to develop more pesticides (including pesticidal pore forming proteins) with distinct modes of action …
Line 498: Nevertheless, the pore hallmarks identified offer a practical filter for mining AFDB and related resources for uncharacterised single-component cytolytic members of the family.
Line 505: The structure and the biophysical characterisation of the Vpb4 subclass also lays the foundation for identification of previously uncharacterised cytolytic pores from the Bacterial_Exotoxin_B family.
Line 707: Database Search for Uncharacterised Bacterial_Exotoxin_B Members

Details on software versions
Line 516: … gene was annotated using Prodigal[64] v2.6.3 to predict CDS of putative protein, followed by protein prediction via NCBI blastx[65] web interface (https://blast.ncbi.nlm.nih.gov/Blast.cgi), and IGV-Web[66] (https://igv.org/app) for gene visualisation.
Line 543: SEC was performed in the Unicorn v5.31 (Cytiva) system.
Line 565: Data collection was performed on the AcquireMP v2024R1 (Refeyn) software. Mass calibration was performed on DiscoverMP v2024R1 (Refeyn) software using three standards: BSA (66 kDa), …
Line 578: Data collection was performed using the EPU Software v.2.12.1.2782 (Thermo Fisher) on Titan Krios

Line 581: Single particle cryo-EM analysis was performed using RELION-5.0 through the M3 MASSIVE High-Performance Computing facility (Supplementary Figure 13).
Line 623: Analysis of the pore lumen diameter was generated in Hole2[80] v2.3.1 and graphed in GraphPad Prism v10.4.1.
Line 639: The current was passed through a digital analog converter (National Instruments BNC-2110) to a PC and was recorded using WinEDR v4.0.4 (University of Strathclyde).
Line 670: The resulting photons were captured by a charge-coupled device (Andor iXon Ultra EMCCD, Oxford Instruments, UK), which was controlled through the Andor Solis I Software v4.29.300012.0 (Oxford Instruments, UK). Post acquisition processing was performed in the Fiji[82] distribution of ImageJ v1.54p and MatLab R2023b (MathWorks).
Line 702: Gaps were then trimmed using trimAL[86] v1.5 in automated mode. The phylogenetic tree was constructed in IQ-TREE[87] v3.0 based on a model generated

Method description on Resazurin assay:
Line 691: Briefly, the culture medium of the cells was replaced with culture medium containing 10 µg/mL resazurin diluted in DPBS (10% v/v), and the cells were incubated at 37 °C for 4 hours. Fluorescence was measured using a Spectramax Gemini EM plate reader (Molecular Devices) at excitation and emission wavelength of 445 nm and 585 nm respectively.

Changes on calcium-binding region:
Line 166: In terms of secondary toxin binding at the funnel region, the Vpb4Aa2 pore was observed to retain the dual-calcium-binding region at Domain 1 deemed important for binding toxins for translocation amongst the family members (Supplementary Figure 4).
Line 168: In comparison to CdtA/CdtB and Iota-A/Iota-B binary toxin systems~~, Vpb4Aa2 shared the calcium binding region at Domain 1 important for binding of the A component. However,~~ the NSS/NSQ residues in the loop previously characterised to clamp the enzymatic partners were not conserved in Vpb4Aa2 (Supplementary Figure 5).
Line 173: Thus, although Vpb4Aa2 conserves the Domain 1 calcium binding regions, the capability of the protein to dock any secondary toxins of the current characterised systems (PA, CdtB, and Iota-B) remains a question due to the lack of α-clamp specific residues and the NSS/NSQ loop.

Other word changes:
Line 194: The Vpb4Aa2 pore does not contain the canonical negatively charged clamps that have been observed in the pore lumen of PA, Iota-B, and CdtB (Figure 3c, Supplementary Figure 7).
Line 351 (partially removed): A phylogenetic tree was then constructed using these AFDB hits, together with 6 members of the Vpb4 subclass taken from the Bacterial Pesticidal Protein Resource Center (BPPRC)~~, filtered at the secondary ranking,~~ (Vpb4Aa1, Vpb4Aa2, Vpb4Ba1, Vpb4Ca1, Vpb4Da2, and Vpb4Fa1), other structurally relevant family members (PA, CdtB, Iota-B, and C2-II), and Vpb1Ac1 as representative of the Vpb1 subclass (Figure 7b).
Line 509: While Vpb4Aa2 targets and its mechanism of interaction with receptors remain to be elucidated, our findings provide a framework on deep database searches for identification and screening strategies for potential pesticidal proteins within the agricultural industry.

Completed Data availability statement, acknowledgements, author contributions, and competing interest.

References:

1 Guo, Y. *et al.* Bacillus thuringiensis toxins with nematocidal activity against the pinewood nematode Bursaphelenchus xylophilus. *Journal of Invertebrate Pathology* **189**, 107726 (2022). https://doi.org/https://doi.org/10.1016/j.jip.2022.107726

2 Zhang, X. *et al.* XaxAB-like binary toxin from Photorhabdus luminescens exhibits both insecticidal activity and cytotoxicity. *FEMS Microbiology Letters* **350**, 48-56 (2014). https://doi.org/10.1111/1574-6968.12321

3 Sastalla, I. *et al.* The Bacillus cereus Hbl and Nhe Tripartite Enterotoxin Components Assemble Sequentially on the Surface of Target Cells and Are Not Interchangeable. *PLOS ONE* **8**, e76955 (2013). https://doi.org/10.1371/journal.pone.0076955

4 Best, H. L. *et al.* The Crystal Structure of Bacillus thuringiensis Tpp80Aa1 and Its Interaction with Galactose-Containing Glycolipids. *Toxins (Basel)* **14** (2022). https://doi.org/10.3390/toxins14120863

5 Colletier, J.-P. *et al.* De novo phasing with X-ray laser reveals mosquito larvicide BinAB structure. *Nature* **539**, 43-47 (2016). https://doi.org/10.1038/nature19825

6 Schnepf, H. E. *et al.* Characterization of Cry34/Cry35 Binary Insecticidal Proteins from Diverse <i>Bacillus thuringiensis</i> Strain Collections. *Applied and Environmental Microbiology* **71**, 1765-1774 (2005). https://doi.org/doi:10.1128/AEM.71.4.1765-1774.2005

7 Williamson, L. J. *et al.* Structure of the <i>Lysinibacillus sphaericus</i> Tpp49Aa1 pesticidal protein elucidated from natural crystals using MHz-SFX. *Proceedings of the National Academy of Sciences* **120**, e2203241120 (2023). https://doi.org/doi:10.1073/pnas.2203241120

8 Hardenbrook, N. J. *et al.* Atomic structures of anthrax toxin protective antigen channels bound to partially unfolded lethal and edema factors. *Nature Communications* **11**, 840 (2020). https://doi.org/10.1038/s41467-020-14658-6

9 Blaustein, R. O., Koehler, T. M., Collier, R. J. & Finkelstein, A. Anthrax toxin: channel-forming activity of protective antigen in planar phospholipid bilayers. *Proceedings of the National Academy of Sciences* **86**, 2209-2213 (1989). https://doi.org/doi:10.1073/pnas.86.7.2209

10 Krantz, B. A. Anthrax Toxin: Model System for Studying Protein Translocation. *Journal of Molecular Biology* **436**, 168521 (2024). https://doi.org/https://doi.org/10.1016/j.jmb.2024.168521

11 Papatheodorou, P. *et al.* Lipolysis-stimulated lipoprotein receptor (LSR) is the host receptor for the binary toxin <i>Clostridium difficile</i> transferase (CDT). *Proceedings of the National Academy of Sciences* **108**, 16422-16427 (2011). https://doi.org/doi:10.1073/pnas.1109772108

12 Kawamoto, A. *et al.* Cryo-EM structures of the translocational binary toxin complex CDTa-bound CDTb-pore from Clostridioides difficile. *Nature Communications* **13**, 6119 (2022). https://doi.org/10.1038/s41467-022-33888-4

13 Landenberger, M. *et al.* The cytotoxic effect of Clostridioides difficile pore-forming toxin CDTb. *Biochimica et Biophysica Acta (BBA) - Biomembranes* **1863**, 183603 (2021). https://doi.org/https://doi.org/10.1016/j.bbamem.2021.183603

14 Salles, I. I. *et al.* Cytotoxic activity of Bacillus anthracis protective antigen observed in a macrophage cell line overexpressing ANTXR1. *Cellular Microbiology* **8**, 1272-1281 (2006). https://doi.org/https://doi.org/10.1111/j.1462-5822.2006.00708.x

15 Yin, Y. *et al.* A new Bacillus thuringiensis protein for Western corn rootworm control. *PLOS ONE* **15**, e0242791 (2020). https://doi.org/10.1371/journal.pone.0242791

16 Alves, G. B. *et al.* Comparative genomic analysis and mosquito larvicidal activity of four Bacillus thuringiensis serovar israelensis strains. *Scientific Reports* **10**, 5518 (2020). https://doi.org/10.1038/s41598-020-60670-7

17 Crickmore, N. *et al.* A structure-based nomenclature for Bacillus thuringiensis and other bacteria-derived pesticidal proteins. *Journal of Invertebrate Pathology* **186**, 107438 (2021). https://doi.org/https://doi.org/10.1016/j.jip.2020.107438
